# Supplementary material for: Improved survival of non-small cell lung cancer patients after introducing patient navigation: A retrospective cohort study with propensity score weighted historic control
Source: PLoS One. 2022 Oct 25;17(10):e0276719. doi: 10.1371/journal.pone.0276719 (PMC9595513; doi:10.1371/journal.pone.0276719)
Supplement: S1 Table — MS Word file, including Table S4a (Overview of diagnostic and treatment procedures, by study cohorts) and Table S4b (Intermediate outcomes at last assessment before treatment initiation, by study cohorts). (DOCX) [file pone.0276719.s001.docx]

**S4 Supplementary tables**

**Table S4a. Overview of diagnostic and treatment procedures, by study cohorts**

|  | | **Control cohort (weighted) (N=173, weights = 119.54207)** | **Intervention cohort (N=123)** | **p*** |
| --- | --- | --- | --- | --- |
| **Completed investigations before treatment initiation** | | | | |
| Chest CT | | 100% | 99.2% | 0.324 |
| Bronchoscopy | | 94.9% | 95.1% | 0.946 |
| PETCT | | 37.6% | 47.2% | 0.144 |
| Scintigraphy | | 4.2% | 2.4% | 0.497 |
| Brain imaging | | 84.3% | 87.8% | 0.421 |
| Cytology confirmation^$^ | | 93.1% | 86.2% | 0.148 |
| AJCC stage or TNM documented | | 69.9% | 70.7% | 0.896 |
| ECOG documented | | 94.8% | 95.9% | 0.713 |
| multidisciplinary Tumour Board treatment recommendation | | 94.5% | 91.9% | 0.468 |
| multidisciplinary Tumour Board treatment recommendation within 30 days from first hospital code | | 43.1% | 32.5% | 0.071 |
| **Treatment procedures** | | | | |
| Surgery | All types | 23.6% | 29.3% | 0.359 |
|  | Segment resection | 3.0% | 5.7% | 0.302 |
|  | Lobectomy | 17.1% | 19.5% | 0.628 |
|  | Pneumonectomy | 2.5% | 1.6% | 0.626 |
|  | Explorative surgery | 1.0% | 2.4% | 0.396 |
| Chemotherapy | | 49.0% | 44.7% | 0.509 |
| Radiotherapy | | 54.0% | 55.3% | 0.847 |
| Any oncology treatment | | 82.4% | 83.7% | 0.790 |
| Any oncology treatment initiated in ≤44 days after first cancer code | | 36.5% | 34.0% | 0.709 |
| Treatment delay from first code, in days: median (quartiles) | | 67 (37 – 112) | 58 (36 – 96) | 0.245 |

$, not including intraoperative evaluation; *Chi-squared test and Kruskal-Wallis rank sum test for categorical and non-normal continuous variables, respectively.

**Table S4b. Intermediate outcomes at last assessment before treatment initiation, by study cohorts**

|  | | **Control cohort (weighted) (N=173, weights = 119.54207)** | **Intervention cohort (N=120)** | **p*** |
| --- | --- | --- | --- | --- |
| Largest tumour diameter in mm, mean (SD) | | 42 (24) | 45 (27) | 0.481 |
| T score | T1 | 8.4% | 17.1% | 0.208 |
|  | T2 | 22.5% | 16.3% |  |
|  | T3 | 19.7% | 14.6% |  |
|  | T4 | 28.9% | 32.5% |  |
|  | not specified | 20.4% | 19.5% |  |
| N score | N0 | 19.3% | 20.3% | 0.184 |
|  | N1 | 12.4% | 5.7% |  |
|  | N2 | 29.5% | 26.8% |  |
|  | N3 | 15.2% | 25.2% |  |
|  | not specified | 23.6% | 22.0% |  |
| M score | M0 | 30.1% | 37.4% | 0.476 |
|  | M1 | 41.1% | 35.0% |  |
|  | not specified | 28.8% | 27.6% |  |
| AJCC stage TNM7 | stage I | 7.0% | 10.6% | 0.705 |
|  | stage II | 4.8% | 4.1% |  |
|  | stage III | 17.0% | 21.1% |  |
|  | stage IV | 41.1% | 35.0% |  |
|  | not specified | 30.1% | 29.3% |  |
| ECOG performance | ECOG 0 | 30.2% | 37.4% | 0.841 |
|  | ECOG 1 | 48.2% | 43.1% |  |
|  | ECOG 2 | 11.2% | 10.6% |  |
|  | ECOG 3-4 | 5.2% | 4.9% |  |
|  | not specified | 5.2% | 4.1% |  |

*Chi-squared test and two-sample t-test for categorical and for normal continuous variables, respectively.
