# Supplementary material for: Improved survival of non-small cell lung cancer patients after introducing patient navigation: A retrospective cohort study with propensity score weighted historic control
Source: PLoS One. 2022 Oct 25;17(10):e0276719. doi: 10.1371/journal.pone.0276719 (PMC9595513; doi:10.1371/journal.pone.0276719)
Supplement: S2 File — Statistical analysis codes and code outputs in R, in a html file. (HTML) [file pone.0276719.s003.html]

OnkoNetwork retrospective study, subgroup analysis of non-small cell lung cancer patients


# OnkoNetwork retrospective study, subgroup analysis of non-small cell lung cancer patients

#### JGP

#### Revised analysis, 08 March 2022

#### Step 1. Data read, propensity score weighting, descriptive analyses

```
## 1. Read data 
library(readxl)
MDS2 <- as.data.frame(read_excel("S1_Datafile.xlsx")) #Main dataset
PS2 <- glm(Intervention ~ Age + Sex + Residence_type + JKM_score + Initial_symptom_group + Days_onset_to_1stcode_cat + Chest.CT.before.1stcode + Brain.imaging.before.1stcode + Bronchoscopy.before.1stcode + PETCT.before.1stcode + Hist_final_groups, data = MDS2, family=binomial)
summary(PS2)
```

```
## 
## Call:
## glm(formula = Intervention ~ Age + Sex + Residence_type + JKM_score + 
##     Initial_symptom_group + Days_onset_to_1stcode_cat + Chest.CT.before.1stcode + 
##     Brain.imaging.before.1stcode + Bronchoscopy.before.1stcode + 
##     PETCT.before.1stcode + Hist_final_groups, family = binomial, 
##     data = MDS2)
## 
## Deviance Residuals: 
##     Min       1Q   Median       3Q      Max  
## -1.7753  -0.9788  -0.7418   1.1909   2.1657  
## 
## Coefficients:
##                                             Estimate Std. Error z value
## (Intercept)                                 0.029172   1.266468   0.023
## Age                                         0.002253   0.014743   0.153
## Sexmale                                    -0.490650   0.262390  -1.870
## Residence_typeurban                         0.370890   0.261424   1.419
## JKM_score                                  -0.000378   0.017501  -0.022
## Initial_symptom_groupmissing               -0.564690   0.813112  -0.694
## Initial_symptom_groupsymptomatic            0.177672   0.277485   0.640
## Days_onset_to_1stcode_cat(36,76.8]          0.174457   0.381333   0.457
## Days_onset_to_1stcode_cat(76.8,778]        -0.387350   0.383048  -1.011
## Days_onset_to_1stcode_cat[0,13]            -0.486104   0.383628  -1.267
## Days_onset_to_1stcode_catmissing           -0.109706   0.496132  -0.221
## Chest.CT.before.1stcodeyes                 -0.640012   0.299100  -2.140
## Brain.imaging.before.1stcodeyes            -0.357540   0.353992  -1.010
## Bronchoscopy.before.1stcodeyes              2.196699   0.674174   3.258
## PETCT.before.1stcodeyes                     0.264465   0.789915   0.335
## Hist_final_groupsneuroendocrine, other_nos -0.072953   0.544965  -0.134
## Hist_final_groupsNSCLC other_nos           -0.599330   0.535856  -1.118
## Hist_final_groupssquamous cell carcinoma    0.154505   0.283124   0.546
##                                            Pr(>|z|)   
## (Intercept)                                 0.98162   
## Age                                         0.87854   
## Sexmale                                     0.06149 . 
## Residence_typeurban                         0.15598   
## JKM_score                                   0.98277   
## Initial_symptom_groupmissing                0.48738   
## Initial_symptom_groupsymptomatic            0.52198   
## Days_onset_to_1stcode_cat(36,76.8]          0.64732   
## Days_onset_to_1stcode_cat(76.8,778]         0.31191   
## Days_onset_to_1stcode_cat[0,13]             0.20511   
## Days_onset_to_1stcode_catmissing            0.82500   
## Chest.CT.before.1stcodeyes                  0.03237 * 
## Brain.imaging.before.1stcodeyes             0.31248   
## Bronchoscopy.before.1stcodeyes              0.00112 **
## PETCT.before.1stcodeyes                     0.73777   
## Hist_final_groupsneuroendocrine, other_nos  0.89351   
## Hist_final_groupsNSCLC other_nos            0.26337   
## Hist_final_groupssquamous cell carcinoma    0.58526   
## ---
## Signif. codes:  0 '***' 0.001 '**' 0.01 '*' 0.05 '.' 0.1 ' ' 1
## 
## (Dispersion parameter for binomial family taken to be 1)
## 
##     Null deviance: 401.86  on 295  degrees of freedom
## Residual deviance: 370.70  on 278  degrees of freedom
## AIC: 406.7
## 
## Number of Fisher Scoring iterations: 4
```

```
MDS2$PS <- PS2$fitted
MDS2$IPTW <- ifelse(MDS2$Intervention ==1,  1, MDS2$PS_all/(1-MDS2$PS_all))

# DESCRIPTIVE ANALYSES ON UNWEIGHTED AND WEIGHTED DATA
demographics <- c("Age", "Sex", "Residence_type", "JKM_score", "Initial_symptom_group", "Hist_final_groups", "Days_onset_to_1stcode_cat", "Chest.CT.before.1stcode", "Bronchoscopy.before.1stcode", "PETCT.before.1stcode", "Scintigraphy.before..1stcode", "Brain.imaging.before.1stcode", "ON", "PS_all")
diagnostics <- c("Chest.CT.before.TX2", "Bronchoscopy.before.TX2", "PETCT.before.TX2",  "Scintigraphy.before.TX2", "Positive.sample.before.TX2", "Brain.imaging.before.TX2", "TB.beforeTX2", "OT_in_30days", "Stagedocumented","ECOG_preTX_documented")
treatment_allpts <- c("Surg_pulm_resection_incl_explorative", "TX_chemo", "TX_radio", "OnkoTXstarted", "TX_in_44days", "Surg_type2")
treatment_TBonly <- c("OnkoTXstarted", "TX_after_OT_14days")
treatment_TXonly <- c("TX_in_44days", "Days_1stcode_to_TX")                 
surgery <- c("Surg_type2", "R_surg")
outcomes <- c("largest.diameter.in.mm","T_preTX2" ,"N_preTX2" ,"M_preTX2", "Stage_AJCCTNM7_short_preTX", 
              "ECOG_preTX_groupped1") 

str(MDS2)
```

```
## 'data.frame':    296 obs. of  43 variables:
##  $ Intervention                : logi  FALSE TRUE TRUE FALSE FALSE TRUE ...
##  $ ON                          : logi  FALSE TRUE TRUE FALSE FALSE TRUE ...
##  $ Age                         : num  83 84 83 81 81 81 79 78 79 79 ...
##  $ Sex                         : chr  "male" "male" "male" "male" ...
##  $ Residence_type              : chr  "rural" "urban" "urban" "urban" ...
##  $ JKM_score                   : num  45.5 45.5 30.6 41.7 57.6 ...
##  $ Initial_symptom_group       : chr  "asymptomatic finding" "symptomatic" "symptomatic" "symptomatic" ...
##  $ Hist_final_groups           : chr  "squamous cell carcinoma" "squamous cell carcinoma" "adenocarcinoma" "squamous cell carcinoma" ...
##  $ Days_onset_to_1stcode_cat   : chr  "(76.8,778]" "(76.8,778]" "(76.8,778]" "[0,13]" ...
##  $ Chest.CT.before.1stcode     : chr  "yes" "no" "yes" "yes" ...
##  $ Brain.imaging.before.1stcode: chr  "no" "no" "no" "yes" ...
##  $ Bronchoscopy.before.1stcode : chr  "no" "no" "no" "no" ...
##  $ PETCT.before.1stcode        : chr  "no" "no" "no" "no" ...
##  $ Scintigraphy.before..1stcode: chr  "no" "no" "no" "no" ...
##  $ PS_all                      : num  0.238 0.507 0.318 0.254 0.402 ...
##  $ IPTW_ATT                    : num  0.312 1 1 0.341 0.673 ...
##  $ Chest.CT.before.TX2         : logi  TRUE TRUE TRUE TRUE TRUE TRUE ...
##  $ Brain.imaging.before.TX2    : logi  FALSE TRUE FALSE TRUE FALSE TRUE ...
##  $ Bronchoscopy.before.TX2     : logi  TRUE TRUE TRUE TRUE TRUE TRUE ...
##  $ PETCT.before.TX2            : logi  FALSE FALSE FALSE FALSE FALSE FALSE ...
##  $ Scintigraphy.before.TX2     : logi  FALSE FALSE FALSE FALSE FALSE FALSE ...
##  $ Positive.sample.before.TX2  : logi  TRUE TRUE TRUE TRUE TRUE TRUE ...
##  $ TB.beforeTX2                : logi  TRUE TRUE TRUE TRUE TRUE TRUE ...
##  $ OT_in_30days                : chr  "yes" "no" "no" "no" ...
##  $ Stagedocumented             : chr  "no" "yes" "yes" "yes" ...
##  $ ECOG_preTX_documented       : logi  FALSE TRUE FALSE TRUE TRUE TRUE ...
##  $ Stage_AJCCTNM7_short_preTX  : chr  "unknown" "IV" "IV" "IV" ...
##  $ T_preTX2                    : chr  "T1" "T4" "T1" "T1" ...
##  $ N_preTX2                    : chr  "range or missing" "N3" "range or missing" "N1" ...
##  $ M_preTX2                    : chr  "M0" "M1" "M1" "M1" ...
##  $ largest.diameter.in.mm      : chr  "23" "60" "10" "16" ...
##  $ ECOG_preTX_groupped1        : chr  "missing" "ECOG3_4" "missing" "ECOG1" ...
##  $ Surg_pulm_resection         : chr  "no" "no" "no" "no" ...
##  $ Surg_type2                  : chr  "none" "none" "none" "none" ...
##  $ TX_chemo                    : chr  "no" "no" "no" "no" ...
##  $ TX_radio                    : chr  "yes" "no" "no" "yes" ...
##  $ OnkoTXstarted               : logi  TRUE FALSE FALSE TRUE TRUE FALSE ...
##  $ First.treatment             : chr  "radio" "untreated" "untreated" "radio" ...
##  $ Days_1stcode_to_TX          : chr  "40" "NA" "NA" "83" ...
##  $ Surv_from_1stcode           : num  812 76 35 84 232 470 438 418 38 303 ...
##  $ VS_event                    : num  0 1 0 0 0 1 1 1 1 0 ...
##  $ PS                          : num  0.238 0.507 0.318 0.254 0.402 ...
##  $ IPTW                        : num  0.312 1 1 0.341 0.673 ...
```

```
MDS2$largest.diameter.in.mm <- as.numeric(MDS2$largest.diameter.in.mm)
```

```
## Warning: NAs introduced by coercion
```

```
MDS2$Days_1stcode_to_TX <- as.numeric(MDS2$Days_1stcode_to_TX)
```

```
## Warning: NAs introduced by coercion
```

```
MDS2$TX_in_44days <- MDS2$Days_1stcode_to_TX <= 44
MDS2$Surg_pulm_resection_incl_explorative <- ifelse(MDS2$Surg_type2 == "none", "no", "yes")

library("tableone")
```

```
## Warning: package 'tableone' was built under R version 4.0.5
```

```
library(survey)
```

```
## Warning: package 'survey' was built under R version 4.0.5
```

```
## Loading required package: grid
```

```
## Loading required package: Matrix
```

```
## Loading required package: survival
```

```
## 
## Attaching package: 'survey'
```

```
## The following object is masked from 'package:graphics':
## 
##     dotchart
```

```
#Demographics:
Model_u_MDS2 <- svydesign(ids = ~ 1, data = MDS2)
```

```
## Warning in svydesign.default(ids = ~1, data = MDS2): No weights or probabilities
## supplied, assuming equal probability
```

```
Model_IPTW_MDS2 <- svydesign(ids = ~ 1, data = MDS2, weights = ~ IPTW)
Tab_u_demographics <- svyCreateTableOne(vars = demographics, strata = "Intervention", data = Model_u_MDS2)
Tab_w_demographics <- svyCreateTableOne(vars = demographics, strata = "Intervention", data = Model_IPTW_MDS2)
print(Tab_u_demographics, test = TRUE, testApprox = chisq.test, smd = FALSE, contDigits = 5, catDigits = 1, pDigits = 8)
```

```
##                                         Stratified by Intervention
##                                          FALSE              
##   n                                      173.00000          
##   Age (mean (SD))                         63.84393 (8.89956)
##   Sex = male (%)                             109.0 (63.0)   
##   Residence_type = urban (%)                  89.0 (51.4)   
##   JKM_score (mean (SD))                   44.15746 (7.36744)
##   Initial_symptom_group (%)                                 
##      asymptomatic finding                     74.0 (42.8)   
##      missing                                   7.0 ( 4.0)   
##      symptomatic                              92.0 (53.2)   
##   Hist_final_groups (%)                                     
##      adenocarcinoma                           77.0 (44.5)   
##      neuroendocrine, other_nos                10.0 ( 5.8)   
##      NSCLC other_nos                          14.0 ( 8.1)   
##      squamous cell carcinoma                  72.0 (41.6)   
##   Days_onset_to_1stcode_cat (%)                             
##      (13,36]                                  37.0 (21.4)   
##      (36,76.8]                                34.0 (19.7)   
##      (76.8,778]                               41.0 (23.7)   
##      [0,13]                                   43.0 (24.9)   
##      missing                                  18.0 (10.4)   
##   Chest.CT.before.1stcode = yes (%)          119.0 (68.8)   
##   Bronchoscopy.before.1stcode = yes (%)        3.0 ( 1.7)   
##   PETCT.before.1stcode = yes (%)               5.0 ( 2.9)   
##   Scintigraphy.before..1stcode = yes (%)       0.0 ( 0.0)   
##   Brain.imaging.before.1stcode = yes (%)      42.0 (24.3)   
##   ON = TRUE (%)                                0.0 ( 0.0)   
##   PS_all (mean (SD))                       0.37325 (0.12738)
##                                         Stratified by Intervention
##                                          TRUE                p           test
##   n                                      123.00000                           
##   Age (mean (SD))                         64.21951 (8.28245)  0.70912673     
##   Sex = male (%)                              64.0 (52.0)     0.06045066     
##   Residence_type = urban (%)                  70.0 (56.9)     0.35426770     
##   JKM_score (mean (SD))                   44.21813 (7.65111)  0.94550754     
##   Initial_symptom_group (%)                                   0.72817729     
##      asymptomatic finding                     48.0 (39.0)                    
##      missing                                   4.0 ( 3.3)                    
##      symptomatic                              71.0 (57.7)                    
##   Hist_final_groups (%)                                       0.87752316     
##      adenocarcinoma                           55.0 (44.7)                    
##      neuroendocrine, other_nos                 8.0 ( 6.5)                    
##      NSCLC other_nos                           7.0 ( 5.7)                    
##      squamous cell carcinoma                  53.0 (43.1)                    
##   Days_onset_to_1stcode_cat (%)                               0.66959622     
##      (13,36]                                  30.0 (24.4)                    
##      (36,76.8]                                30.0 (24.4)                    
##      (76.8,778]                               25.0 (20.3)                    
##      [0,13]                                   24.0 (19.5)                    
##      missing                                  14.0 (11.4)                    
##   Chest.CT.before.1stcode = yes (%)           70.0 (56.9)     0.03726884     
##   Bronchoscopy.before.1stcode = yes (%)       14.0 (11.4)     0.00051823     
##   PETCT.before.1stcode = yes (%)               4.0 ( 3.3)     0.85854127     
##   Scintigraphy.before..1stcode = yes (%)       1.0 ( 0.8)     0.23581185     
##   Brain.imaging.before.1stcode = yes (%)      21.0 (17.1)     0.13734396     
##   ON = TRUE (%)                              119.0 (96.7)    <0.00000001     
##   PS_all (mean (SD))                       0.47502 (0.17485)  0.00000008
```

```
print(Tab_w_demographics, test = TRUE, testApprox = chisq.test, smd = FALSE, contDigits = 5, catDigits = 1, pDigits = 8)
```

```
##                                         Stratified by Intervention
##                                          FALSE              
##   n                                      119.54207          
##   Age (mean (SD))                         64.87370 (8.60086)
##   Sex = male (%)                              66.0 (55.2)   
##   Residence_type = urban (%)                  67.1 (56.1)   
##   JKM_score (mean (SD))                   44.21294 (6.88550)
##   Initial_symptom_group (%)                                 
##      asymptomatic finding                     47.4 (39.6)   
##      missing                                   2.2 ( 1.9)   
##      symptomatic                              69.9 (58.5)   
##   Hist_final_groups (%)                                     
##      adenocarcinoma                           52.4 (43.9)   
##      neuroendocrine, other_nos                 7.8 ( 6.5)   
##      NSCLC other_nos                           7.3 ( 6.1)   
##      squamous cell carcinoma                  52.0 (43.5)   
##   Days_onset_to_1stcode_cat (%)                             
##      (13,36]                                  29.3 (24.5)   
##      (36,76.8]                                27.3 (22.8)   
##      (76.8,778]                               24.2 (20.2)   
##      [0,13]                                   23.0 (19.3)   
##      missing                                  15.7 (13.2)   
##   Chest.CT.before.1stcode = yes (%)           67.4 (56.3)   
##   Bronchoscopy.before.1stcode = yes (%)       10.3 ( 8.6)   
##   PETCT.before.1stcode = yes (%)               4.9 ( 4.1)   
##   Scintigraphy.before..1stcode = yes (%)       0.0 ( 0.0)   
##   Brain.imaging.before.1stcode = yes (%)      25.5 (21.3)   
##   ON = TRUE (%)                                0.0 ( 0.0)   
##   PS_all (mean (SD))                       0.45983 (0.15348)
##                                         Stratified by Intervention
##                                          TRUE                p           test
##   n                                      123.00000                           
##   Age (mean (SD))                         64.21951 (8.28245)  0.53563818     
##   Sex = male (%)                              64.0 (52.0)     0.63211753     
##   Residence_type = urban (%)                  70.0 (56.9)     0.90693822     
##   JKM_score (mean (SD))                   44.21813 (7.65111)  0.99546717     
##   Initial_symptom_group (%)                                   0.74726707     
##      asymptomatic finding                     48.0 (39.0)                    
##      missing                                   4.0 ( 3.3)                    
##      symptomatic                              71.0 (57.7)                    
##   Hist_final_groups (%)                                       0.99858570     
##      adenocarcinoma                           55.0 (44.7)                    
##      neuroendocrine, other_nos                 8.0 ( 6.5)                    
##      NSCLC other_nos                           7.0 ( 5.7)                    
##      squamous cell carcinoma                  53.0 (43.1)                    
##   Days_onset_to_1stcode_cat (%)                               0.99442503     
##      (13,36]                                  30.0 (24.4)                    
##      (36,76.8]                                30.0 (24.4)                    
##      (76.8,778]                               25.0 (20.3)                    
##      [0,13]                                   24.0 (19.5)                    
##      missing                                  14.0 (11.4)                    
##   Chest.CT.before.1stcode = yes (%)           70.0 (56.9)     0.93049031     
##   Bronchoscopy.before.1stcode = yes (%)       14.0 (11.4)     0.63415073     
##   PETCT.before.1stcode = yes (%)               4.0 ( 3.3)     0.77314966     
##   Scintigraphy.before..1stcode = yes (%)       1.0 ( 0.8)     0.32389517     
##   Brain.imaging.before.1stcode = yes (%)      21.0 (17.1)     0.44790947     
##   ON = TRUE (%)                              119.0 (96.7)    <0.00000001     
##   PS_all (mean (SD))                       0.47502 (0.17485)  0.53662674
```

```
#Rubins's B: 
RubinB_unweighted_NSCLC <- round(abs(mean(MDS2$PS[MDS2$Intervention == T]) - mean(MDS2$PS[MDS2$Intervention == F]))*100 / (sqrt((var(MDS2$PS[MDS2$Intervention == T]) + var(MDS2$PS[MDS2$Intervention == F]))/2)), digits = 3)
library(Hmisc);
```

```
## Warning: package 'Hmisc' was built under R version 4.0.5
```

```
## Loading required package: lattice
```

```
## Loading required package: Formula
```

```
## Loading required package: ggplot2
```

```
## 
## Attaching package: 'Hmisc'
```

```
## The following object is masked from 'package:survey':
## 
##     deff
```

```
## The following objects are masked from 'package:base':
## 
##     format.pval, units
```

```
mi0_w_wod <- wtd.mean(MDS2$PS[MDS2$Intervention == F], MDS2$IPTW[MDS2$Intervention == F]); 
vi0_w_wod <- wtd.var(MDS2$PS[MDS2$Intervention == F], MDS2$IPTW[MDS2$Intervention == F])
RubinB_weighted_NSCLC <- round(abs(mean(MDS2$PS[MDS2$Intervention == T]) - mi0_w_wod)*100 / (sqrt((var(MDS2$PS[MDS2$Intervention == T]) + vi0_w_wod)/2)), digits = 3); 
RubinB_unweighted_NSCLC
```

```
## [1] 66.526
```

```
RubinB_weighted_NSCLC
```

```
## [1] 9.226
```

```
#Rubin's R: 
RubinR_unweighted_NSCLC <- round(ifelse(var(MDS2$PS[MDS2$Intervention == T]) > var(MDS2$PS[MDS2$Intervention == F]), var(MDS2$PS[MDS2$Intervention == T])/var(MDS2$PS[MDS2$Intervention == F]),var(MDS2$PS[MDS2$Intervention == F])/var(MDS2$PS[MDS2$Intervention == T])), digits = 3)
RubinR_weighted_NSCLC <- round(ifelse(var(MDS2$PS[MDS2$Intervention == T]) > vi0_w_wod, var(MDS2$PS[MDS2$Intervention == T])/vi0_w_wod, vi0_w_wod/var(MDS2$PS[MDS2$Intervention == T])), digits = 3)
unloadNamespace("Hmisc") #remove, since it blocks the "Quantile" command from DescTools
RubinR_unweighted_NSCLC
```

```
## [1] 1.884
```

```
RubinR_weighted_NSCLC
```

```
## [1] 1.294
```

```
#Diagnostics in the weighted sample
Tab_w_diagnostics <- svyCreateTableOne(vars = diagnostics, strata = "Intervention", data = Model_IPTW_MDS2)
print(Tab_w_diagnostics, test = TRUE, smd = FALSE, contDigits = 5, catDigits = 1, pDigits = 8)
```

```
##                                        Stratified by Intervention
##                                         FALSE          TRUE         
##   n                                     119.5          123.0        
##   Chest.CT.before.TX2 = TRUE (%)        119.5 (100.0)  122.0 (99.2) 
##   Bronchoscopy.before.TX2 = TRUE (%)    113.5 ( 94.9)  117.0 (95.1) 
##   PETCT.before.TX2 = TRUE (%)            44.9 ( 37.6)   58.0 (47.2) 
##   Scintigraphy.before.TX2 = TRUE (%)      5.0 (  4.2)    3.0 ( 2.4) 
##   Positive.sample.before.TX2 = TRUE (%) 111.3 ( 93.1)  106.0 (86.2) 
##   Brain.imaging.before.TX2 = TRUE (%)   100.8 ( 84.3)  108.0 (87.8) 
##   TB.beforeTX2 = TRUE (%)               112.9 ( 94.5)  113.0 (91.9) 
##   OT_in_30days = yes (%)                 51.6 ( 43.1)   40.0 (32.5) 
##   Stagedocumented = yes (%)              83.6 ( 69.9)   87.0 (70.7) 
##   ECOG_preTX_documented = TRUE (%)      113.4 ( 94.8)  118.0 (95.9) 
##                                        Stratified by Intervention
##                                         p           test
##   n                                                     
##   Chest.CT.before.TX2 = TRUE (%)         0.32389517     
##   Bronchoscopy.before.TX2 = TRUE (%)     0.94617417     
##   PETCT.before.TX2 = TRUE (%)            0.14373402     
##   Scintigraphy.before.TX2 = TRUE (%)     0.49720580     
##   Positive.sample.before.TX2 = TRUE (%)  0.14816933     
##   Brain.imaging.before.TX2 = TRUE (%)    0.42053605     
##   TB.beforeTX2 = TRUE (%)                0.46817510     
##   OT_in_30days = yes (%)                 0.09413197     
##   Stagedocumented = yes (%)              0.89643572     
##   ECOG_preTX_documented = TRUE (%)       0.71339360
```

```
#Diagnostics, non-surgery pts subgroup
Model_IPTW_nonsurg <- svydesign(ids = ~ 1, data = MDS2[MDS2$Surg_type2 == "none",], weights = ~ IPTW_ATT)
Tab_w_diagnostics_nonsurg <- svyCreateTableOne(vars = "Positive.sample.before.TX2", strata = "Intervention", data = Model_IPTW_nonsurg)
print(Tab_w_diagnostics_nonsurg, test = TRUE, smd = FALSE, contDigits = 5, catDigits = 1, pDigits = 8)
```

```
##                                        Stratified by Intervention
##                                         FALSE                      
##   n                                     3303666788978880.5         
##   Positive.sample.before.TX2 = TRUE (%) 3303666788978880.0 (100.0) 
##                                        Stratified by Intervention
##                                         TRUE         p           test
##   n                                     87.0                         
##   Positive.sample.before.TX2 = TRUE (%) 85.0 (97.7)  <0.00000001
```

```
#treatment modalities in the weighted sample
Tab_w_treatment_allpts <- svyCreateTableOne(vars = treatment_allpts, strata = "Intervention", data = Model_IPTW_MDS2)
print(Tab_w_treatment_allpts, test = TRUE, testApprox = chisq.test, smd = FALSE, contDigits = 5, catDigits = 1, pDigits = 8)
```

```
##                                                 Stratified by Intervention
##                                                  FALSE         TRUE         
##   n                                              119.5         123.0        
##   Surg_pulm_resection_incl_explorative = yes (%)  28.2 (23.6)   36.0 (29.3) 
##   TX_chemo = yes (%)                              58.6 (49.0)   55.0 (44.7) 
##   TX_radio = yes (%)                              64.6 (54.0)   68.0 (55.3) 
##   OnkoTXstarted = TRUE (%)                        98.5 (82.4)  103.0 (83.7) 
##   TX_in_44days = TRUE (%)                         35.9 (36.5)   35.0 (34.0) 
##   Surg_type2 (%)                                                            
##      explorative                                   1.2 ( 1.0)    3.0 ( 2.4) 
##      lobectomy                                    20.4 (17.1)   24.0 (19.5) 
##      none                                         91.3 (76.4)   87.0 (70.7) 
##      pneumonectomy                                 3.0 ( 2.5)    2.0 ( 1.6) 
##      segment resection                             3.6 ( 3.0)    7.0 ( 5.7) 
##                                                 Stratified by Intervention
##                                                  p           test
##   n                                                              
##   Surg_pulm_resection_incl_explorative = yes (%)  0.35932798     
##   TX_chemo = yes (%)                              0.50876387     
##   TX_radio = yes (%)                              0.84745983     
##   OnkoTXstarted = TRUE (%)                        0.79020902     
##   TX_in_44days = TRUE (%)                         0.70948335     
##   Surg_type2 (%)                                  0.62686844     
##      explorative                                                 
##      lobectomy                                                   
##      none                                                        
##      pneumonectomy                                               
##      segment resection
```

```
#weighted Chi-sqared tests on specific surgery types

library(weights)
```

```
## Warning: package 'weights' was built under R version 4.0.5
```

```
## Loading required package: Hmisc
```

```
## Warning: package 'Hmisc' was built under R version 4.0.5
```

```
## 
## Attaching package: 'Hmisc'
```

```
## The following object is masked from 'package:survey':
## 
##     deff
```

```
## The following objects are masked from 'package:base':
## 
##     format.pval, units
```

```
wtd.chi.sq(MDS2$Intervention, MDS2$Surg_type2 == "segment resection", weight=MDS2$IPTW, na.rm=TRUE, drop.missing.levels=TRUE, mean1=FALSE)
```

```
##     Chisq        df   p.value 
## 1.0644004 1.0000000 0.3022137
```

```
wtd.chi.sq(MDS2$Intervention, MDS2$Surg_type2 == "lobectomy", weight=MDS2$IPTW, na.rm=TRUE, drop.missing.levels=TRUE, mean1=FALSE)
```

```
##     Chisq        df   p.value 
## 0.2350678 1.0000000 0.6277913
```

```
wtd.chi.sq(MDS2$Intervention, MDS2$Surg_type2 == "pneumonectomy", weight=MDS2$IPTW, na.rm=TRUE, drop.missing.levels=TRUE, mean1=FALSE)
```

```
##     Chisq        df   p.value 
## 0.2375218 1.0000000 0.6260018
```

```
wtd.chi.sq(MDS2$Intervention, MDS2$Surg_type2 == "explorative", weight=MDS2$IPTW, na.rm=TRUE, drop.missing.levels=TRUE, mean1=FALSE)
```

```
##     Chisq        df   p.value 
## 0.7203610 1.0000000 0.3960255
```

```
unloadNamespace("weights") #remove, since it blocks the "Quantile" command from DescTools
unloadNamespace("Hmisc") #remove, since it blocks the "Quantile" command from DescTools

#weighted medians, IQRs, and Kruskal-Wallis rank sum test results for treatment delay:
library(DescTools)
```

```
## Warning: package 'DescTools' was built under R version 4.0.5
```

```
## Registered S3 method overwritten by 'DescTools':
##   method         from 
##   reorder.factor gdata
```

```
Tab_w_treatment_txonly <- svyCreateTableOne(vars = treatment_TXonly, strata = "Intervention", data = Model_IPTW_MDS2[is.na(MDS2$Days_1stcode_to_TX) == F,])
print(Tab_w_treatment_txonly, test = TRUE, testApprox = chisq.test, nonnormal = c("Days_1stcode_to_TX"), smd = FALSE, contDigits = 5, catDigits = 1, pDigits = 8)
```

```
##                                    Stratified by Intervention
##                                     FALSE                         
##   n                                     98.5                      
##   TX_in_44days = TRUE (%)               35.9 (36.5)               
##   Days_1stcode_to_TX (median [IQR]) 67.29982 [37.00000, 112.32133]
##                                    Stratified by Intervention
##                                     TRUE                           p          
##   n                                     103.0                                 
##   TX_in_44days = TRUE (%)                35.0 (34.0)                0.70953536
##   Days_1stcode_to_TX (median [IQR])  58.00000 [35.75000, 95.75000]  0.24492906
##                                    Stratified by Intervention
##                                     test   
##   n                                        
##   TX_in_44days = TRUE (%)                  
##   Days_1stcode_to_TX (median [IQR]) nonnorm
```

```
#Outcomes data in the weighted sample
Model_IPTW_MDS2_o <- svydesign(ids = ~ 1, data = MDS2, weights = ~ IPTW)
Tab_w_outcomes <- svyCreateTableOne(vars = outcomes, strata = "Intervention", data = Model_IPTW_MDS2_o)
print(Tab_w_outcomes, test = TRUE, smd = FALSE, contDigits = 5, catDigits = 1, pDigits = 8)
```

```
##                                     Stratified by Intervention
##                                      FALSE                TRUE                
##   n                                  119.54207            123.00000           
##   largest.diameter.in.mm (mean (SD))  42.45695 (24.40621)  44.85981 (26.65794)
##   T_preTX2 (%)                                                                
##      range or missing                     24.4 (20.4)          24.0 (19.5)    
##      T1                                   10.1 ( 8.4)          21.0 (17.1)    
##      T2                                   26.8 (22.5)          20.0 (16.3)    
##      T3                                   23.6 (19.7)          18.0 (14.6)    
##      T4                                   34.6 (28.9)          40.0 (32.5)    
##   N_preTX2 (%)                                                                
##      N0                                   23.1 (19.3)          25.0 (20.3)    
##      N1                                   14.8 (12.4)           7.0 ( 5.7)    
##      N2                                   35.2 (29.5)          33.0 (26.8)    
##      N3                                   18.2 (15.2)          31.0 (25.2)    
##      range or missing                     28.3 (23.6)          27.0 (22.0)    
##   M_preTX2 (%)                                                                
##      M0                                   36.0 (30.1)          46.0 (37.4)    
##      M1                                   49.1 (41.1)          43.0 (35.0)    
##      Mx                                   34.5 (28.8)          34.0 (27.6)    
##   Stage_AJCCTNM7_short_preTX (%)                                              
##      I                                     8.4 ( 7.0)          13.0 (10.6)    
##      II                                    5.7 ( 4.8)           5.0 ( 4.1)    
##      III                                  20.4 (17.0)          26.0 (21.1)    
##      IV                                   49.1 (41.1)          43.0 (35.0)    
##      unknown                              36.0 (30.1)          36.0 (29.3)    
##   ECOG_preTX_groupped1 (%)                                                    
##      ECOG0                                36.1 (30.2)          46.0 (37.4)    
##      ECOG1                                57.7 (48.2)          53.0 (43.1)    
##      ECOG2                                13.4 (11.2)          13.0 (10.6)    
##      ECOG3_4                               6.2 ( 5.2)           6.0 ( 4.9)    
##      missing                               6.2 ( 5.2)           5.0 ( 4.1)    
##                                     Stratified by Intervention
##                                      p           test
##   n                                                  
##   largest.diameter.in.mm (mean (SD))  0.48082108     
##   T_preTX2 (%)                        0.20846663     
##      range or missing                                
##      T1                                              
##      T2                                              
##      T3                                              
##      T4                                              
##   N_preTX2 (%)                        0.18438390     
##      N0                                              
##      N1                                              
##      N2                                              
##      N3                                              
##      range or missing                                
##   M_preTX2 (%)                        0.47553699     
##      M0                                              
##      M1                                              
##      Mx                                              
##   Stage_AJCCTNM7_short_preTX (%)      0.70484413     
##      I                                               
##      II                                              
##      III                                             
##      IV                                              
##      unknown                                         
##   ECOG_preTX_groupped1 (%)            0.84157381     
##      ECOG0                                           
##      ECOG1                                           
##      ECOG2                                           
##      ECOG3_4                                         
##      missing
```

```
##### kaplan Meier plots
library(survival)
library(survminer)
```

```
## Loading required package: ggpubr
```

```
## Registered S3 methods overwritten by 'car':
##   method                          from
##   influence.merMod                lme4
##   cooks.distance.influence.merMod lme4
##   dfbeta.influence.merMod         lme4
##   dfbetas.influence.merMod        lme4
```

```
#unweighted
SF_VS_u <- survfit(Surv(Surv_from_1stcode/365, VS_event) ~ Intervention, data = MDS2)
KM_VS_u <- ggsurvplot(SF_VS_u, pval = F, conf.int = F, risk.table = T, risk.table.col = "strata",  risk.table.y.text = FALSE,  linetype = "strata", surv.median.line = "none", ggtheme = theme_bw(), palette = c("darkgrey", "black"), break.time.by = 0.5, xlim = c(0, 4),legend.labs = c("Control", "Intervention")) + xlab("Years")
```

```
## Warning: Vectorized input to `element_text()` is not officially supported.
## Results may be unexpected or may change in future versions of ggplot2.
```

```
print(KM_VS_u)
```

```
#weighted
SF_VS_w <- survfit(Surv(Surv_from_1stcode/365, VS_event) ~ Intervention, data = MDS2, weights = IPTW)
KM_VS_w <- ggsurvplot(SF_VS_w, pval = F, conf.int = F, risk.table = TRUE, risk.table.col = "strata", risk.table.y.text = FALSE,  linetype = "strata", surv.median.line = "none", ggtheme = theme_bw(), palette = c("darkgrey", "black"), break.time.by = 0.5, xlim = c(0, 4), legend.labs = c("Control", "Intervention")) + xlab("Years")
```

```
## Warning: Vectorized input to `element_text()` is not officially supported.
## Results may be unexpected or may change in future versions of ggplot2.
```

```
print(KM_VS_w)
```

#### Step 2. Weighted Cox regression analysis of overall survival

```
MDS2$PTID.x <- rownames(MDS2)

summary(coxph(Surv(Surv_from_1stcode, VS_event) ~ Intervention,  weights = IPTW, data = MDS2, cluster = PTID.x))
```

```
## Call:
## coxph(formula = Surv(Surv_from_1stcode, VS_event) ~ Intervention, 
##     data = MDS2, weights = IPTW, cluster = PTID.x)
## 
##   n= 296, number of events= 123 
## 
##                     coef exp(coef) se(coef) robust se      z Pr(>|z|)  
## InterventionTRUE -0.4487    0.6385   0.2007    0.2044 -2.195   0.0282 *
## ---
## Signif. codes:  0 '***' 0.001 '**' 0.01 '*' 0.05 '.' 0.1 ' ' 1
## 
##                  exp(coef) exp(-coef) lower .95 upper .95
## InterventionTRUE    0.6385      1.566    0.4277    0.9531
## 
## Concordance= 0.542  (se = 0.027 )
## Likelihood ratio test= 5.1  on 1 df,   p=0.02
## Wald test            = 4.82  on 1 df,   p=0.03
## Score (logrank) test = 5.08  on 1 df,   p=0.02,   Robust = 4.84  p=0.03
## 
##   (Note: the likelihood ratio and score tests assume independence of
##      observations within a cluster, the Wald and robust score tests do not).
```

```
summary(coxph(Surv(Surv_from_1stcode, VS_event) ~ Age,  weights = IPTW, data = MDS2, cluster = PTID.x))
```

```
## Call:
## coxph(formula = Surv(Surv_from_1stcode, VS_event) ~ Age, data = MDS2, 
##     weights = IPTW, cluster = PTID.x)
## 
##   n= 296, number of events= 123 
## 
##        coef exp(coef) se(coef) robust se     z Pr(>|z|)  
## Age 0.02841   1.02881  0.01250   0.01281 2.217   0.0266 *
## ---
## Signif. codes:  0 '***' 0.001 '**' 0.01 '*' 0.05 '.' 0.1 ' ' 1
## 
##     exp(coef) exp(-coef) lower .95 upper .95
## Age     1.029      0.972     1.003     1.055
## 
## Concordance= 0.556  (se = 0.031 )
## Likelihood ratio test= 5.18  on 1 df,   p=0.02
## Wald test            = 4.91  on 1 df,   p=0.03
## Score (logrank) test = 5.16  on 1 df,   p=0.02,   Robust = 4.66  p=0.03
## 
##   (Note: the likelihood ratio and score tests assume independence of
##      observations within a cluster, the Wald and robust score tests do not).
```

```
summary(coxph(Surv(Surv_from_1stcode, VS_event) ~ Sex,  weights = IPTW, data = MDS2, cluster = PTID.x))
```

```
## Call:
## coxph(formula = Surv(Surv_from_1stcode, VS_event) ~ Sex, data = MDS2, 
##     weights = IPTW, cluster = PTID.x)
## 
##   n= 296, number of events= 123 
## 
##            coef exp(coef) se(coef) robust se     z Pr(>|z|)
## Sexmale 0.08272   1.08623  0.19766   0.20637 0.401    0.689
## 
##         exp(coef) exp(-coef) lower .95 upper .95
## Sexmale     1.086     0.9206    0.7249     1.628
## 
## Concordance= 0.495  (se = 0.027 )
## Likelihood ratio test= 0.18  on 1 df,   p=0.7
## Wald test            = 0.16  on 1 df,   p=0.7
## Score (logrank) test = 0.18  on 1 df,   p=0.7,   Robust = 0.16  p=0.7
## 
##   (Note: the likelihood ratio and score tests assume independence of
##      observations within a cluster, the Wald and robust score tests do not).
```

```
summary(coxph(Surv(Surv_from_1stcode, VS_event) ~ Residence_type,  weights = IPTW, data = MDS2, cluster = PTID.x))
```

```
## Call:
## coxph(formula = Surv(Surv_from_1stcode, VS_event) ~ Residence_type, 
##     data = MDS2, weights = IPTW, cluster = PTID.x)
## 
##   n= 296, number of events= 123 
## 
##                       coef exp(coef) se(coef) robust se     z Pr(>|z|)
## Residence_typeurban 0.3385    1.4029   0.2035    0.2165 1.564    0.118
## 
##                     exp(coef) exp(-coef) lower .95 upper .95
## Residence_typeurban     1.403     0.7128    0.9179     2.144
## 
## Concordance= 0.528  (se = 0.028 )
## Likelihood ratio test= 2.84  on 1 df,   p=0.09
## Wald test            = 2.45  on 1 df,   p=0.1
## Score (logrank) test = 2.79  on 1 df,   p=0.09,   Robust = 2.59  p=0.1
## 
##   (Note: the likelihood ratio and score tests assume independence of
##      observations within a cluster, the Wald and robust score tests do not).
```

```
summary(coxph(Surv(Surv_from_1stcode, VS_event) ~ JKM_score,  weights = IPTW, data = MDS2, cluster = PTID.x))
```

```
## Call:
## coxph(formula = Surv(Surv_from_1stcode, VS_event) ~ JKM_score, 
##     data = MDS2, weights = IPTW, cluster = PTID.x)
## 
##   n= 296, number of events= 123 
## 
##              coef exp(coef) se(coef) robust se     z Pr(>|z|)
## JKM_score 0.01157   1.01163  0.01416   0.01294 0.894    0.371
## 
##           exp(coef) exp(-coef) lower .95 upper .95
## JKM_score     1.012     0.9885    0.9863     1.038
## 
## Concordance= 0.507  (se = 0.029 )
## Likelihood ratio test= 0.67  on 1 df,   p=0.4
## Wald test            = 0.8  on 1 df,   p=0.4
## Score (logrank) test = 0.67  on 1 df,   p=0.4,   Robust = 0.8  p=0.4
## 
##   (Note: the likelihood ratio and score tests assume independence of
##      observations within a cluster, the Wald and robust score tests do not).
```

```
summary(coxph(Surv(Surv_from_1stcode, VS_event) ~ Hist_final_groups,  weights = IPTW, data = MDS2, cluster = PTID.x))
```

```
## Call:
## coxph(formula = Surv(Surv_from_1stcode, VS_event) ~ Hist_final_groups, 
##     data = MDS2, weights = IPTW, cluster = PTID.x)
## 
##   n= 296, number of events= 123 
## 
##                                                coef exp(coef) se(coef)
## Hist_final_groupsneuroendocrine, other_nos -0.13863   0.87055  0.42287
## Hist_final_groupsNSCLC other_nos            0.09549   1.10020  0.44167
## Hist_final_groupssquamous cell carcinoma    0.06548   1.06767  0.20910
##                                            robust se      z Pr(>|z|)
## Hist_final_groupsneuroendocrine, other_nos   0.42466 -0.326    0.744
## Hist_final_groupsNSCLC other_nos             0.46947  0.203    0.839
## Hist_final_groupssquamous cell carcinoma     0.21425  0.306    0.760
## 
##                                            exp(coef) exp(-coef) lower .95
## Hist_final_groupsneuroendocrine, other_nos    0.8705     1.1487    0.3787
## Hist_final_groupsNSCLC other_nos              1.1002     0.9089    0.4384
## Hist_final_groupssquamous cell carcinoma      1.0677     0.9366    0.7016
##                                            upper .95
## Hist_final_groupsneuroendocrine, other_nos     2.001
## Hist_final_groupsNSCLC other_nos               2.761
## Hist_final_groupssquamous cell carcinoma       1.625
## 
## Concordance= 0.516  (se = 0.028 )
## Likelihood ratio test= 0.31  on 3 df,   p=1
## Wald test            = 0.31  on 3 df,   p=1
## Score (logrank) test = 0.31  on 3 df,   p=1,   Robust = 0.32  p=1
## 
##   (Note: the likelihood ratio and score tests assume independence of
##      observations within a cluster, the Wald and robust score tests do not).
```

```
summary(coxph(Surv(Surv_from_1stcode, VS_event) ~ Initial_symptom_group,  weights = IPTW, data = MDS2, cluster = PTID.x))
```

```
## Call:
## coxph(formula = Surv(Surv_from_1stcode, VS_event) ~ Initial_symptom_group, 
##     data = MDS2, weights = IPTW, cluster = PTID.x)
## 
##   n= 296, number of events= 123 
## 
##                                    coef exp(coef) se(coef) robust se     z
## Initial_symptom_groupmissing     0.6847    1.9831   0.5545    0.5646 1.213
## Initial_symptom_groupsymptomatic 0.6865    1.9868   0.2159    0.2317 2.963
##                                  Pr(>|z|)   
## Initial_symptom_groupmissing      0.22526   
## Initial_symptom_groupsymptomatic  0.00305 **
## ---
## Signif. codes:  0 '***' 0.001 '**' 0.01 '*' 0.05 '.' 0.1 ' ' 1
## 
##                                  exp(coef) exp(-coef) lower .95 upper .95
## Initial_symptom_groupmissing         1.983     0.5043    0.6558     5.997
## Initial_symptom_groupsymptomatic     1.987     0.5033    1.2616     3.129
## 
## Concordance= 0.577  (se = 0.026 )
## Likelihood ratio test= 11.03  on 2 df,   p=0.004
## Wald test            = 8.9  on 2 df,   p=0.01
## Score (logrank) test = 10.68  on 2 df,   p=0.005,   Robust = 10.28  p=0.006
## 
##   (Note: the likelihood ratio and score tests assume independence of
##      observations within a cluster, the Wald and robust score tests do not).
```

```
summary(coxph(Surv(Surv_from_1stcode, VS_event) ~ Days_onset_to_1stcode_cat,  weights = IPTW, data = MDS2, cluster = PTID.x))
```

```
## Call:
## coxph(formula = Surv(Surv_from_1stcode, VS_event) ~ Days_onset_to_1stcode_cat, 
##     data = MDS2, weights = IPTW, cluster = PTID.x)
## 
##   n= 296, number of events= 123 
## 
##                                         coef exp(coef) se(coef) robust se
## Days_onset_to_1stcode_cat(36,76.8]  -0.04257   0.95832  0.27798   0.28521
## Days_onset_to_1stcode_cat(76.8,778] -0.26817   0.76478  0.30126   0.30671
## Days_onset_to_1stcode_cat[0,13]      0.02443   1.02473  0.28908   0.28602
## Days_onset_to_1stcode_catmissing    -0.39898   0.67100  0.33769   0.35343
##                                          z Pr(>|z|)
## Days_onset_to_1stcode_cat(36,76.8]  -0.149    0.881
## Days_onset_to_1stcode_cat(76.8,778] -0.874    0.382
## Days_onset_to_1stcode_cat[0,13]      0.085    0.932
## Days_onset_to_1stcode_catmissing    -1.129    0.259
## 
##                                     exp(coef) exp(-coef) lower .95 upper .95
## Days_onset_to_1stcode_cat(36,76.8]     0.9583     1.0435    0.5479     1.676
## Days_onset_to_1stcode_cat(76.8,778]    0.7648     1.3076    0.4192     1.395
## Days_onset_to_1stcode_cat[0,13]        1.0247     0.9759    0.5850     1.795
## Days_onset_to_1stcode_catmissing       0.6710     1.4903    0.3357     1.341
## 
## Concordance= 0.552  (se = 0.029 )
## Likelihood ratio test= 2.45  on 4 df,   p=0.7
## Wald test            = 2.31  on 4 df,   p=0.7
## Score (logrank) test = 2.36  on 4 df,   p=0.7,   Robust = 2.31  p=0.7
## 
##   (Note: the likelihood ratio and score tests assume independence of
##      observations within a cluster, the Wald and robust score tests do not).
```

```
summary(coxph(Surv(Surv_from_1stcode, VS_event) ~ Chest.CT.before.1stcode,  weights = IPTW, data = MDS2, cluster = PTID.x))
```

```
## Call:
## coxph(formula = Surv(Surv_from_1stcode, VS_event) ~ Chest.CT.before.1stcode, 
##     data = MDS2, weights = IPTW, cluster = PTID.x)
## 
##   n= 296, number of events= 123 
## 
##                               coef exp(coef) se(coef) robust se      z Pr(>|z|)
## Chest.CT.before.1stcodeyes -0.6149    0.5407   0.1973    0.2007 -3.064  0.00218
##                              
## Chest.CT.before.1stcodeyes **
## ---
## Signif. codes:  0 '***' 0.001 '**' 0.01 '*' 0.05 '.' 0.1 ' ' 1
## 
##                            exp(coef) exp(-coef) lower .95 upper .95
## Chest.CT.before.1stcodeyes    0.5407       1.85    0.3648    0.8013
## 
## Concordance= 0.579  (se = 0.026 )
## Likelihood ratio test= 9.75  on 1 df,   p=0.002
## Wald test            = 9.39  on 1 df,   p=0.002
## Score (logrank) test = 10.02  on 1 df,   p=0.002,   Robust = 9.21  p=0.002
## 
##   (Note: the likelihood ratio and score tests assume independence of
##      observations within a cluster, the Wald and robust score tests do not).
```

```
summary(coxph(Surv(Surv_from_1stcode, VS_event) ~ Bronchoscopy.before.1stcode,  weights = IPTW, data = MDS2, cluster = PTID.x))
```

```
## Call:
## coxph(formula = Surv(Surv_from_1stcode, VS_event) ~ Bronchoscopy.before.1stcode, 
##     data = MDS2, weights = IPTW, cluster = PTID.x)
## 
##   n= 296, number of events= 123 
## 
##                                   coef exp(coef) se(coef) robust se      z
## Bronchoscopy.before.1stcodeyes -0.5732    0.5637   0.3958    0.6348 -0.903
##                                Pr(>|z|)
## Bronchoscopy.before.1stcodeyes    0.367
## 
##                                exp(coef) exp(-coef) lower .95 upper .95
## Bronchoscopy.before.1stcodeyes    0.5637      1.774    0.1624     1.956
## 
## Concordance= 0.518  (se = 0.023 )
## Likelihood ratio test= 2.48  on 1 df,   p=0.1
## Wald test            = 0.82  on 1 df,   p=0.4
## Score (logrank) test = 2.15  on 1 df,   p=0.1,   Robust = 1.07  p=0.3
## 
##   (Note: the likelihood ratio and score tests assume independence of
##      observations within a cluster, the Wald and robust score tests do not).
```

```
summary(coxph(Surv(Surv_from_1stcode, VS_event) ~ PETCT.before.1stcode,  weights = IPTW, data = MDS2, cluster = PTID.x))
```

```
## Call:
## coxph(formula = Surv(Surv_from_1stcode, VS_event) ~ PETCT.before.1stcode, 
##     data = MDS2, weights = IPTW, cluster = PTID.x)
## 
##   n= 296, number of events= 123 
## 
##                            coef exp(coef) se(coef) robust se      z Pr(>|z|)
## PETCT.before.1stcodeyes -1.2226    0.2945   0.8724    0.8712 -1.403    0.161
## 
##                         exp(coef) exp(-coef) lower .95 upper .95
## PETCT.before.1stcodeyes    0.2945      3.396   0.05339     1.624
## 
## Concordance= 0.514  (se = 0.011 )
## Likelihood ratio test= 3.03  on 1 df,   p=0.08
## Wald test            = 1.97  on 1 df,   p=0.2
## Score (logrank) test = 2.22  on 1 df,   p=0.1,   Robust = 2.22  p=0.1
## 
##   (Note: the likelihood ratio and score tests assume independence of
##      observations within a cluster, the Wald and robust score tests do not).
```

```
summary(coxph(Surv(Surv_from_1stcode, VS_event) ~ Brain.imaging.before.1stcode,  weights = IPTW, data = MDS2, cluster = PTID.x))
```

```
## Call:
## coxph(formula = Surv(Surv_from_1stcode, VS_event) ~ Brain.imaging.before.1stcode, 
##     data = MDS2, weights = IPTW, cluster = PTID.x)
## 
##   n= 296, number of events= 123 
## 
##                                    coef exp(coef) se(coef) robust se     z
## Brain.imaging.before.1stcodeyes -0.1429    0.8669   0.2505    0.2915 -0.49
##                                 Pr(>|z|)
## Brain.imaging.before.1stcodeyes    0.624
## 
##                                 exp(coef) exp(-coef) lower .95 upper .95
## Brain.imaging.before.1stcodeyes    0.8669      1.154    0.4896     1.535
## 
## Concordance= 0.515  (se = 0.023 )
## Likelihood ratio test= 0.33  on 1 df,   p=0.6
## Wald test            = 0.24  on 1 df,   p=0.6
## Score (logrank) test = 0.33  on 1 df,   p=0.6,   Robust = 0.25  p=0.6
## 
##   (Note: the likelihood ratio and score tests assume independence of
##      observations within a cluster, the Wald and robust score tests do not).
```

```
summary(coxph(Surv(Surv_from_1stcode, VS_event) ~ Chest.CT.before.TX2,  weights = IPTW, data = MDS2, cluster = PTID.x))
```

```
## Warning in fitter(X, Y, istrat, offset, init, control, weights = weights, :
## Loglik converged before variable 1 ; coefficient may be infinite.
```

```
## Call:
## coxph(formula = Surv(Surv_from_1stcode, VS_event) ~ Chest.CT.before.TX2, 
##     data = MDS2, weights = IPTW, cluster = PTID.x)
## 
##   n= 296, number of events= 123 
## 
##                              coef exp(coef)  se(coef) robust se     z Pr(>|z|)
## Chest.CT.before.TX2TRUE 1.501e+01 3.304e+06 2.511e+03 1.007e+00 14.91   <2e-16
##                            
## Chest.CT.before.TX2TRUE ***
## ---
## Signif. codes:  0 '***' 0.001 '**' 0.01 '*' 0.05 '.' 0.1 ' ' 1
## 
##                         exp(coef) exp(-coef) lower .95 upper .95
## Chest.CT.before.TX2TRUE   3304187  3.026e-07    459184  23776171
## 
## Concordance= 0.503  (se = 0.003 )
## Likelihood ratio test= 1.04  on 1 df,   p=0.3
## Wald test            = 222.2  on 1 df,   p=<2e-16
## Score (logrank) test = 0.52  on 1 df,   p=0.5,   Robust = 1  p=0.3
## 
##   (Note: the likelihood ratio and score tests assume independence of
##      observations within a cluster, the Wald and robust score tests do not).
```

```
summary(coxph(Surv(Surv_from_1stcode, VS_event) ~ Bronchoscopy.before.TX2,  weights = IPTW, data = MDS2, cluster = PTID.x))
```

```
## Call:
## coxph(formula = Surv(Surv_from_1stcode, VS_event) ~ Bronchoscopy.before.TX2, 
##     data = MDS2, weights = IPTW, cluster = PTID.x)
## 
##   n= 296, number of events= 123 
## 
##                               coef exp(coef) se(coef) robust se     z Pr(>|z|)
## Bronchoscopy.before.TX2TRUE 0.1201    1.1276   0.5050    0.4905 0.245    0.807
## 
##                             exp(coef) exp(-coef) lower .95 upper .95
## Bronchoscopy.before.TX2TRUE     1.128     0.8869    0.4311     2.949
## 
## Concordance= 0.503  (se = 0.01 )
## Likelihood ratio test= 0.06  on 1 df,   p=0.8
## Wald test            = 0.06  on 1 df,   p=0.8
## Score (logrank) test = 0.06  on 1 df,   p=0.8,   Robust = 0.06  p=0.8
## 
##   (Note: the likelihood ratio and score tests assume independence of
##      observations within a cluster, the Wald and robust score tests do not).
```

```
summary(coxph(Surv(Surv_from_1stcode, VS_event) ~ PETCT.before.TX2,  weights = IPTW, data = MDS2, cluster = PTID.x))
```

```
## Call:
## coxph(formula = Surv(Surv_from_1stcode, VS_event) ~ PETCT.before.TX2, 
##     data = MDS2, weights = IPTW, cluster = PTID.x)
## 
##   n= 296, number of events= 123 
## 
##                         coef exp(coef) se(coef) robust se      z Pr(>|z|)    
## PETCT.before.TX2TRUE -1.1108    0.3293   0.2149    0.2076 -5.349 8.82e-08 ***
## ---
## Signif. codes:  0 '***' 0.001 '**' 0.01 '*' 0.05 '.' 0.1 ' ' 1
## 
##                      exp(coef) exp(-coef) lower .95 upper .95
## PETCT.before.TX2TRUE    0.3293      3.037    0.2192    0.4947
## 
## Concordance= 0.646  (se = 0.021 )
## Likelihood ratio test= 29.32  on 1 df,   p=6e-08
## Wald test            = 28.62  on 1 df,   p=9e-08
## Score (logrank) test = 29.36  on 1 df,   p=6e-08,   Robust = 27.57  p=2e-07
## 
##   (Note: the likelihood ratio and score tests assume independence of
##      observations within a cluster, the Wald and robust score tests do not).
```

```
summary(coxph(Surv(Surv_from_1stcode, VS_event) ~ Brain.imaging.before.TX2,  weights = IPTW, data = MDS2, cluster = PTID.x))
```

```
## Call:
## coxph(formula = Surv(Surv_from_1stcode, VS_event) ~ Brain.imaging.before.TX2, 
##     data = MDS2, weights = IPTW, cluster = PTID.x)
## 
##   n= 296, number of events= 123 
## 
##                                coef exp(coef) se(coef) robust se     z Pr(>|z|)
## Brain.imaging.before.TX2TRUE 0.1159    1.1229   0.3037    0.3190 0.363    0.716
## 
##                              exp(coef) exp(-coef) lower .95 upper .95
## Brain.imaging.before.TX2TRUE     1.123     0.8906    0.6009     2.098
## 
## Concordance= 0.497  (se = 0.02 )
## Likelihood ratio test= 0.15  on 1 df,   p=0.7
## Wald test            = 0.13  on 1 df,   p=0.7
## Score (logrank) test = 0.15  on 1 df,   p=0.7,   Robust = 0.14  p=0.7
## 
##   (Note: the likelihood ratio and score tests assume independence of
##      observations within a cluster, the Wald and robust score tests do not).
```

```
summary(coxph(Surv(Surv_from_1stcode, VS_event) ~ Positive.sample.before.TX2,  weights = IPTW, data = MDS2, cluster = PTID.x))
```

```
## Call:
## coxph(formula = Surv(Surv_from_1stcode, VS_event) ~ Positive.sample.before.TX2, 
##     data = MDS2, weights = IPTW, cluster = PTID.x)
## 
##   n= 296, number of events= 123 
## 
##                                  coef exp(coef) se(coef) robust se     z
## Positive.sample.before.TX2TRUE 1.1129    3.0432   0.4179    0.4114 2.705
##                                Pr(>|z|)   
## Positive.sample.before.TX2TRUE  0.00682 **
## ---
## Signif. codes:  0 '***' 0.001 '**' 0.01 '*' 0.05 '.' 0.1 ' ' 1
## 
##                                exp(coef) exp(-coef) lower .95 upper .95
## Positive.sample.before.TX2TRUE     3.043     0.3286     1.359     6.815
## 
## Concordance= 0.55  (se = 0.016 )
## Likelihood ratio test= 9.89  on 1 df,   p=0.002
## Wald test            = 7.32  on 1 df,   p=0.007
## Score (logrank) test = 7.85  on 1 df,   p=0.005,   Robust = 7.57  p=0.006
## 
##   (Note: the likelihood ratio and score tests assume independence of
##      observations within a cluster, the Wald and robust score tests do not).
```

```
summary(coxph(Surv(Surv_from_1stcode, VS_event) ~ Stagedocumented,  weights = IPTW, data = MDS2, cluster = PTID.x))
```

```
## Call:
## coxph(formula = Surv(Surv_from_1stcode, VS_event) ~ Stagedocumented, 
##     data = MDS2, weights = IPTW, cluster = PTID.x)
## 
##   n= 296, number of events= 123 
## 
##                      coef exp(coef) se(coef) robust se     z Pr(>|z|)
## Stagedocumentedyes 0.2835    1.3278   0.2221    0.2533 1.119    0.263
## 
##                    exp(coef) exp(-coef) lower .95 upper .95
## Stagedocumentedyes     1.328     0.7531    0.8082     2.181
## 
## Concordance= 0.52  (se = 0.027 )
## Likelihood ratio test= 1.7  on 1 df,   p=0.2
## Wald test            = 1.25  on 1 df,   p=0.3
## Score (logrank) test = 1.64  on 1 df,   p=0.2,   Robust = 1.37  p=0.2
## 
##   (Note: the likelihood ratio and score tests assume independence of
##      observations within a cluster, the Wald and robust score tests do not).
```

```
summary(coxph(Surv(Surv_from_1stcode, VS_event) ~ TB.beforeTX2,  weights = IPTW, data = MDS2, cluster = PTID.x))
```

```
## Call:
## coxph(formula = Surv(Surv_from_1stcode, VS_event) ~ TB.beforeTX2, 
##     data = MDS2, weights = IPTW, cluster = PTID.x)
## 
##   n= 296, number of events= 123 
## 
##                     coef exp(coef) se(coef) robust se      z Pr(>|z|)
## TB.beforeTX2TRUE -0.2524    0.7769   0.4579    0.4849 -0.521    0.603
## 
##                  exp(coef) exp(-coef) lower .95 upper .95
## TB.beforeTX2TRUE    0.7769      1.287    0.3004      2.01
## 
## Concordance= 0.508  (se = 0.013 )
## Likelihood ratio test= 0.28  on 1 df,   p=0.6
## Wald test            = 0.27  on 1 df,   p=0.6
## Score (logrank) test = 0.31  on 1 df,   p=0.6,   Robust = 0.24  p=0.6
## 
##   (Note: the likelihood ratio and score tests assume independence of
##      observations within a cluster, the Wald and robust score tests do not).
```

```
summary(coxph(Surv(Surv_from_1stcode, VS_event) ~ Stage_AJCCTNM7_short_preTX,  weights = IPTW, data = MDS2, cluster = PTID.x))
```

```
## Call:
## coxph(formula = Surv(Surv_from_1stcode, VS_event) ~ Stage_AJCCTNM7_short_preTX, 
##     data = MDS2, weights = IPTW, cluster = PTID.x)
## 
##   n= 296, number of events= 123 
## 
##                                     coef exp(coef) se(coef) robust se     z
## Stage_AJCCTNM7_short_preTXII      0.5777    1.7820   0.6945    0.6272 0.921
## Stage_AJCCTNM7_short_preTXIII     1.0073    2.7383   0.5251    0.4697 2.145
## Stage_AJCCTNM7_short_preTXIV      1.9445    6.9900   0.4911    0.4347 4.473
## Stage_AJCCTNM7_short_preTXunknown 1.0243    2.7852   0.5030    0.4732 2.165
##                                   Pr(>|z|)    
## Stage_AJCCTNM7_short_preTXII        0.3570    
## Stage_AJCCTNM7_short_preTXIII       0.0320 *  
## Stage_AJCCTNM7_short_preTXIV       7.7e-06 ***
## Stage_AJCCTNM7_short_preTXunknown   0.0304 *  
## ---
## Signif. codes:  0 '***' 0.001 '**' 0.01 '*' 0.05 '.' 0.1 ' ' 1
## 
##                                   exp(coef) exp(-coef) lower .95 upper .95
## Stage_AJCCTNM7_short_preTXII          1.782     0.5612    0.5213     6.092
## Stage_AJCCTNM7_short_preTXIII         2.738     0.3652    1.0906     6.875
## Stage_AJCCTNM7_short_preTXIV          6.990     0.1431    2.9818    16.386
## Stage_AJCCTNM7_short_preTXunknown     2.785     0.3590    1.1018     7.041
## 
## Concordance= 0.655  (se = 0.023 )
## Likelihood ratio test= 33.83  on 4 df,   p=8e-07
## Wald test            = 35.98  on 4 df,   p=3e-07
## Score (logrank) test = 35.43  on 4 df,   p=4e-07,   Robust = 34.44  p=6e-07
## 
##   (Note: the likelihood ratio and score tests assume independence of
##      observations within a cluster, the Wald and robust score tests do not).
```

```
summary(coxph(Surv(Surv_from_1stcode, VS_event) ~ ECOG_preTX_groupped1,  weights = IPTW, data = MDS2, cluster = PTID.x))
```

```
## Call:
## coxph(formula = Surv(Surv_from_1stcode, VS_event) ~ ECOG_preTX_groupped1, 
##     data = MDS2, weights = IPTW, cluster = PTID.x)
## 
##   n= 296, number of events= 123 
## 
##                               coef exp(coef) se(coef) robust se     z Pr(>|z|)
## ECOG_preTX_groupped1ECOG1   0.8913    2.4383   0.2429    0.2690 3.314 0.000921
## ECOG_preTX_groupped1ECOG2   1.3831    3.9872   0.3317    0.3621 3.819 0.000134
## ECOG_preTX_groupped1ECOG3_4 2.2538    9.5237   0.3718    0.3408 6.614 3.75e-11
## ECOG_preTX_groupped1missing 0.1969    1.2176   0.6522    0.6898 0.285 0.775322
##                                
## ECOG_preTX_groupped1ECOG1   ***
## ECOG_preTX_groupped1ECOG2   ***
## ECOG_preTX_groupped1ECOG3_4 ***
## ECOG_preTX_groupped1missing    
## ---
## Signif. codes:  0 '***' 0.001 '**' 0.01 '*' 0.05 '.' 0.1 ' ' 1
## 
##                             exp(coef) exp(-coef) lower .95 upper .95
## ECOG_preTX_groupped1ECOG1       2.438     0.4101     1.439     4.131
## ECOG_preTX_groupped1ECOG2       3.987     0.2508     1.961     8.108
## ECOG_preTX_groupped1ECOG3_4     9.524     0.1050     4.883    18.573
## ECOG_preTX_groupped1missing     1.218     0.8213     0.315     4.706
## 
## Concordance= 0.67  (se = 0.028 )
## Likelihood ratio test= 39.04  on 4 df,   p=7e-08
## Wald test            = 47.76  on 4 df,   p=1e-09
## Score (logrank) test = 52.42  on 4 df,   p=1e-10,   Robust = 30.51  p=4e-06
## 
##   (Note: the likelihood ratio and score tests assume independence of
##      observations within a cluster, the Wald and robust score tests do not).
```

```
summary(coxph(Surv(Surv_from_1stcode, VS_event) ~ Surg_pulm_resection,  weights = IPTW, data = MDS2, cluster = PTID.x))
```

```
## Call:
## coxph(formula = Surv(Surv_from_1stcode, VS_event) ~ Surg_pulm_resection, 
##     data = MDS2, weights = IPTW, cluster = PTID.x)
## 
##   n= 296, number of events= 123 
## 
##                           coef exp(coef) se(coef) robust se      z Pr(>|z|)    
## Surg_pulm_resectionyes -1.6824    0.1859   0.3099    0.4071 -4.132 3.59e-05 ***
## ---
## Signif. codes:  0 '***' 0.001 '**' 0.01 '*' 0.05 '.' 0.1 ' ' 1
## 
##                        exp(coef) exp(-coef) lower .95 upper .95
## Surg_pulm_resectionyes    0.1859      5.378   0.08371     0.413
## 
## Concordance= 0.632  (se = 0.024 )
## Likelihood ratio test= 42.76  on 1 df,   p=6e-11
## Wald test            = 17.08  on 1 df,   p=4e-05
## Score (logrank) test = 36.33  on 1 df,   p=2e-09,   Robust = 34.45  p=4e-09
## 
##   (Note: the likelihood ratio and score tests assume independence of
##      observations within a cluster, the Wald and robust score tests do not).
```

```
summary(coxph(Surv(Surv_from_1stcode, VS_event) ~ TX_chemo,  weights = IPTW, data = MDS2, cluster = PTID.x))
```

```
## Call:
## coxph(formula = Surv(Surv_from_1stcode, VS_event) ~ TX_chemo, 
##     data = MDS2, weights = IPTW, cluster = PTID.x)
## 
##   n= 296, number of events= 123 
## 
##                coef exp(coef) se(coef) robust se      z Pr(>|z|)  
## TX_chemoyes -0.4550    0.6345   0.1980    0.1973 -2.306   0.0211 *
## ---
## Signif. codes:  0 '***' 0.001 '**' 0.01 '*' 0.05 '.' 0.1 ' ' 1
## 
##             exp(coef) exp(-coef) lower .95 upper .95
## TX_chemoyes    0.6345      1.576     0.431     0.934
## 
## Concordance= 0.59  (se = 0.024 )
## Likelihood ratio test= 5.33  on 1 df,   p=0.02
## Wald test            = 5.32  on 1 df,   p=0.02
## Score (logrank) test = 5.37  on 1 df,   p=0.02,   Robust = 4.97  p=0.03
## 
##   (Note: the likelihood ratio and score tests assume independence of
##      observations within a cluster, the Wald and robust score tests do not).
```

```
summary(coxph(Surv(Surv_from_1stcode, VS_event) ~ TX_radio,  weights = IPTW, data = MDS2, cluster = PTID.x))
```

```
## Call:
## coxph(formula = Surv(Surv_from_1stcode, VS_event) ~ TX_radio, 
##     data = MDS2, weights = IPTW, cluster = PTID.x)
## 
##   n= 296, number of events= 123 
## 
##                coef exp(coef) se(coef) robust se     z Pr(>|z|)
## TX_radioyes 0.01647   1.01661  0.19911   0.21070 0.078    0.938
## 
##             exp(coef) exp(-coef) lower .95 upper .95
## TX_radioyes     1.017     0.9837    0.6727     1.536
## 
## Concordance= 0.473  (se = 0.027 )
## Likelihood ratio test= 0.01  on 1 df,   p=0.9
## Wald test            = 0.01  on 1 df,   p=0.9
## Score (logrank) test = 0.01  on 1 df,   p=0.9,   Robust = 0.01  p=0.9
## 
##   (Note: the likelihood ratio and score tests assume independence of
##      observations within a cluster, the Wald and robust score tests do not).
```

```
Surv_multi_baseline <- coxph(Surv(Surv_from_1stcode, VS_event) ~ Intervention + Age + Sex + Residence_type + JKM_score + Initial_symptom_group + Days_onset_to_1stcode_cat + Chest.CT.before.1stcode + Brain.imaging.before.1stcode + Bronchoscopy.before.1stcode + PETCT.before.1stcode + Hist_final_groups,  weights = IPTW, data = MDS2, cluster = PTID.x)
summary(Surv_multi_baseline)
```

```
## Call:
## coxph(formula = Surv(Surv_from_1stcode, VS_event) ~ Intervention + 
##     Age + Sex + Residence_type + JKM_score + Initial_symptom_group + 
##     Days_onset_to_1stcode_cat + Chest.CT.before.1stcode + Brain.imaging.before.1stcode + 
##     Bronchoscopy.before.1stcode + PETCT.before.1stcode + Hist_final_groups, 
##     data = MDS2, weights = IPTW, cluster = PTID.x)
## 
##   n= 296, number of events= 123 
## 
##                                                coef exp(coef) se(coef)
## InterventionTRUE                           -0.45657   0.63345  0.20547
## Age                                         0.02163   1.02186  0.01299
## Sexmale                                    -0.02238   0.97786  0.21599
## Residence_typeurban                         0.30473   1.35626  0.21584
## JKM_score                                   0.01178   1.01185  0.01752
## Initial_symptom_groupmissing                0.91429   2.49500  0.62282
## Initial_symptom_groupsymptomatic            0.59735   1.81729  0.24951
## Days_onset_to_1stcode_cat(36,76.8]         -0.05257   0.94879  0.28293
## Days_onset_to_1stcode_cat(76.8,778]        -0.30414   0.73776  0.31142
## Days_onset_to_1stcode_cat[0,13]            -0.01745   0.98270  0.31335
## Days_onset_to_1stcode_catmissing           -0.41212   0.66225  0.38942
## Chest.CT.before.1stcodeyes                 -0.55772   0.57251  0.24142
## Brain.imaging.before.1stcodeyes             0.32726   1.38716  0.30030
## Bronchoscopy.before.1stcodeyes              0.17144   1.18702  0.46619
## PETCT.before.1stcodeyes                    -0.92236   0.39758  0.95320
## Hist_final_groupsneuroendocrine, other_nos -0.21404   0.80732  0.44453
## Hist_final_groupsNSCLC other_nos            0.09767   1.10260  0.46293
## Hist_final_groupssquamous cell carcinoma    0.03250   1.03304  0.23962
##                                            robust se      z Pr(>|z|)  
## InterventionTRUE                             0.22065 -2.069   0.0385 *
## Age                                          0.01249  1.731   0.0834 .
## Sexmale                                      0.21248 -0.105   0.9161  
## Residence_typeurban                          0.19857  1.535   0.1249  
## JKM_score                                    0.01577  0.747   0.4550  
## Initial_symptom_groupmissing                 0.60179  1.519   0.1287  
## Initial_symptom_groupsymptomatic             0.24817  2.407   0.0161 *
## Days_onset_to_1stcode_cat(36,76.8]           0.27171 -0.193   0.8466  
## Days_onset_to_1stcode_cat(76.8,778]          0.31750 -0.958   0.3381  
## Days_onset_to_1stcode_cat[0,13]              0.31157 -0.056   0.9553  
## Days_onset_to_1stcode_catmissing             0.39857 -1.034   0.3011  
## Chest.CT.before.1stcodeyes                   0.22252 -2.506   0.0122 *
## Brain.imaging.before.1stcodeyes              0.30028  1.090   0.2758  
## Bronchoscopy.before.1stcodeyes               0.69157  0.248   0.8042  
## PETCT.before.1stcodeyes                      1.19376 -0.773   0.4397  
## Hist_final_groupsneuroendocrine, other_nos   0.42160 -0.508   0.6117  
## Hist_final_groupsNSCLC other_nos             0.42681  0.229   0.8190  
## Hist_final_groupssquamous cell carcinoma     0.23124  0.141   0.8882  
## ---
## Signif. codes:  0 '***' 0.001 '**' 0.01 '*' 0.05 '.' 0.1 ' ' 1
## 
##                                            exp(coef) exp(-coef) lower .95
## InterventionTRUE                              0.6335     1.5786   0.41105
## Age                                           1.0219     0.9786   0.99715
## Sexmale                                       0.9779     1.0226   0.64478
## Residence_typeurban                           1.3563     0.7373   0.91901
## JKM_score                                     1.0119     0.9883   0.98105
## Initial_symptom_groupmissing                  2.4950     0.4008   0.76706
## Initial_symptom_groupsymptomatic              1.8173     0.5503   1.11734
## Days_onset_to_1stcode_cat(36,76.8]            0.9488     1.0540   0.55705
## Days_onset_to_1stcode_cat(76.8,778]           0.7378     1.3555   0.39596
## Days_onset_to_1stcode_cat[0,13]               0.9827     1.0176   0.53359
## Days_onset_to_1stcode_catmissing              0.6622     1.5100   0.30322
## Chest.CT.before.1stcodeyes                    0.5725     1.7467   0.37015
## Brain.imaging.before.1stcodeyes               1.3872     0.7209   0.77008
## Bronchoscopy.before.1stcodeyes                1.1870     0.8424   0.30605
## PETCT.before.1stcodeyes                       0.3976     2.5152   0.03831
## Hist_final_groupsneuroendocrine, other_nos    0.8073     1.2387   0.35333
## Hist_final_groupsNSCLC other_nos              1.1026     0.9069   0.47766
## Hist_final_groupssquamous cell carcinoma      1.0330     0.9680   0.65658
##                                            upper .95
## InterventionTRUE                              0.9762
## Age                                           1.0472
## Sexmale                                       1.4830
## Residence_typeurban                           2.0016
## JKM_score                                     1.0436
## Initial_symptom_groupmissing                  8.1154
## Initial_symptom_groupsymptomatic              2.9557
## Days_onset_to_1stcode_cat(36,76.8]            1.6160
## Days_onset_to_1stcode_cat(76.8,778]           1.3746
## Days_onset_to_1stcode_cat[0,13]               1.8098
## Days_onset_to_1stcode_catmissing              1.4464
## Chest.CT.before.1stcodeyes                    0.8855
## Brain.imaging.before.1stcodeyes               2.4988
## Bronchoscopy.before.1stcodeyes                4.6039
## PETCT.before.1stcodeyes                       4.1263
## Hist_final_groupsneuroendocrine, other_nos    1.8446
## Hist_final_groupsNSCLC other_nos              2.5452
## Hist_final_groupssquamous cell carcinoma      1.6253
## 
## Concordance= 0.65  (se = 0.027 )
## Likelihood ratio test= 34.17  on 18 df,   p=0.01
## Wald test            = 46.01  on 18 df,   p=3e-04
## Score (logrank) test = 33.02  on 18 df,   p=0.02,   Robust = 30.76  p=0.03
## 
##   (Note: the likelihood ratio and score tests assume independence of
##      observations within a cluster, the Wald and robust score tests do not).
```

```
 #the intervention coefficient remained unchanged, since the sample was well balanced by the weights for baseline parameters. In the next model including post-baseline variables, baseline vars will not be included.
```

```
Model_postbaselinevars_full <- coxph(Surv(Surv_from_1stcode, VS_event) ~ Intervention + Bronchoscopy.before.TX2 + PETCT.before.TX2 + Brain.imaging.before.TX2 + Positive.sample.before.TX2 + Stagedocumented + TB.beforeTX2 + Stage_AJCCTNM7_short_preTX + ECOG_preTX_groupped1 + Surg_pulm_resection + TX_chemo + TX_radio, data = MDS2, weights = IPTW, cluster = PTID.x)
summary(Model_postbaselinevars_full)
```

```
## Call:
## coxph(formula = Surv(Surv_from_1stcode, VS_event) ~ Intervention + 
##     Bronchoscopy.before.TX2 + PETCT.before.TX2 + Brain.imaging.before.TX2 + 
##     Positive.sample.before.TX2 + Stagedocumented + TB.beforeTX2 + 
##     Stage_AJCCTNM7_short_preTX + ECOG_preTX_groupped1 + Surg_pulm_resection + 
##     TX_chemo + TX_radio, data = MDS2, weights = IPTW, cluster = PTID.x)
## 
##   n= 296, number of events= 123 
## 
##                                       coef exp(coef) se(coef) robust se      z
## InterventionTRUE                  -0.20891   0.81147  0.21118   0.22561 -0.926
## Bronchoscopy.before.TX2TRUE        0.52848   1.69635  0.54932   0.44341  1.192
## PETCT.before.TX2TRUE              -0.47280   0.62326  0.24592   0.28544 -1.656
## Brain.imaging.before.TX2TRUE      -0.06325   0.93871  0.31949   0.34522 -0.183
## Positive.sample.before.TX2TRUE    -0.03934   0.96142  0.52558   0.56749 -0.069
## Stagedocumentedyes                -0.91488   0.40056  0.51429   0.40657 -2.250
## TB.beforeTX2TRUE                  -0.40328   0.66812  0.49678   0.45165 -0.893
## Stage_AJCCTNM7_short_preTXII       0.70933   2.03263  0.71425   0.63202  1.122
## Stage_AJCCTNM7_short_preTXIII      0.57576   1.77848  0.55576   0.42113  1.367
## Stage_AJCCTNM7_short_preTXIV       1.25537   3.50912  0.52379   0.37291  3.366
## Stage_AJCCTNM7_short_preTXunknown       NA        NA  0.00000   0.00000     NA
## ECOG_preTX_groupped1ECOG1          0.50366   1.65477  0.25916   0.25269  1.993
## ECOG_preTX_groupped1ECOG2          0.54220   1.71979  0.38543   0.45160  1.201
## ECOG_preTX_groupped1ECOG3_4        1.28725   3.62282  0.46307   0.47125  2.732
## ECOG_preTX_groupped1missing        0.30816   1.36092  0.71267   0.72562  0.425
## Surg_pulm_resectionyes            -1.08502   0.33789  0.44710   0.61718 -1.758
## TX_chemoyes                       -0.53302   0.58683  0.23917   0.23648 -2.254
## TX_radioyes                       -0.16368   0.84901  0.23285   0.24954 -0.656
##                                   Pr(>|z|)    
## InterventionTRUE                  0.354477    
## Bronchoscopy.before.TX2TRUE       0.233315    
## PETCT.before.TX2TRUE              0.097639 .  
## Brain.imaging.before.TX2TRUE      0.854627    
## Positive.sample.before.TX2TRUE    0.944731    
## Stagedocumentedyes                0.024433 *  
## TB.beforeTX2TRUE                  0.371910    
## Stage_AJCCTNM7_short_preTXII      0.261724    
## Stage_AJCCTNM7_short_preTXIII     0.171570    
## Stage_AJCCTNM7_short_preTXIV      0.000762 ***
## Stage_AJCCTNM7_short_preTXunknown       NA    
## ECOG_preTX_groupped1ECOG1         0.046236 *  
## ECOG_preTX_groupped1ECOG2         0.229899    
## ECOG_preTX_groupped1ECOG3_4       0.006304 ** 
## ECOG_preTX_groupped1missing       0.671066    
## Surg_pulm_resectionyes            0.078741 .  
## TX_chemoyes                       0.024198 *  
## TX_radioyes                       0.511863    
## ---
## Signif. codes:  0 '***' 0.001 '**' 0.01 '*' 0.05 '.' 0.1 ' ' 1
## 
##                                   exp(coef) exp(-coef) lower .95 upper .95
## InterventionTRUE                     0.8115     1.2323    0.5215    1.2627
## Bronchoscopy.before.TX2TRUE          1.6964     0.5895    0.7114    4.0453
## PETCT.before.TX2TRUE                 0.6233     1.6045    0.3562    1.0905
## Brain.imaging.before.TX2TRUE         0.9387     1.0653    0.4772    1.8467
## Positive.sample.before.TX2TRUE       0.9614     1.0401    0.3161    2.9239
## Stagedocumentedyes                   0.4006     2.4965    0.1806    0.8887
## TB.beforeTX2TRUE                     0.6681     1.4967    0.2757    1.6192
## Stage_AJCCTNM7_short_preTXII         2.0326     0.4920    0.5890    7.0151
## Stage_AJCCTNM7_short_preTXIII        1.7785     0.5623    0.7791    4.0599
## Stage_AJCCTNM7_short_preTXIV         3.5091     0.2850    1.6896    7.2883
## Stage_AJCCTNM7_short_preTXunknown        NA         NA        NA        NA
## ECOG_preTX_groupped1ECOG1            1.6548     0.6043    1.0084    2.7154
## ECOG_preTX_groupped1ECOG2            1.7198     0.5815    0.7097    4.1676
## ECOG_preTX_groupped1ECOG3_4          3.6228     0.2760    1.4385    9.1239
## ECOG_preTX_groupped1missing          1.3609     0.7348    0.3282    5.6426
## Surg_pulm_resectionyes               0.3379     2.9595    0.1008    1.1327
## TX_chemoyes                          0.5868     1.7041    0.3692    0.9328
## TX_radioyes                          0.8490     1.1778    0.5206    1.3846
## 
## Concordance= 0.768  (se = 0.024 )
## Likelihood ratio test= 85.6  on 17 df,   p=4e-11
## Wald test            = 90.14  on 17 df,   p=6e-12
## Score (logrank) test = 90.99  on 17 df,   p=4e-12,   Robust = 67.03  p=7e-08
## 
##   (Note: the likelihood ratio and score tests assume independence of
##      observations within a cluster, the Wald and robust score tests do not).
```

```
# OnkoNetwork survival benefits may be partly explained by post-baseline interim outcomes: broader use of PET-CT in diagnostics, stage shift towards stages I-III versus stage IV at the end of diagnostic period, higher pulmonary resection rate, and/or use of chemotherapy (significant predictors of survival).
```
