# Supplementary material for: Improved survival of non-small cell lung cancer patients after introducing patient navigation: A retrospective cohort study with propensity score weighted historic control
Source: PLoS One. 2022 Oct 25;17(10):e0276719. doi: 10.1371/journal.pone.0276719 (PMC9595513; doi:10.1371/journal.pone.0276719)
Supplement: S3 File — MS Word file including Stata log and do files. (DOCX) [file pone.0276719.s004.docx]

S3 Statistical methods and results in Stata

-------------------------------------------------------------------------------------

Stata DO file:

log using "c:\DATA\OneDrive - SYREON Kft\Safe Syreon\syreon\horizon2020\selfie\elemzes\retro_tudo_plusz201910\selfie_retro_pulmo_plus20200924.log", replace

*selfie_retro_pulmo_plus20200924.do

set more off

clear

import excel "c:\DATA\OneDrive - SYREON Kft\Safe Syreon\syreon\horizon2020\selfie\elemzes\retro_tudo_plusz201910\data\pulmo_retro20200904.xlsx" , sheet("maiadat") firstrow

encode PTID, generate (PTID2)

rename VS_event death

rename Surv_from_1stcode fuptime

insp death

insp fuptime

*SURVIVAL

*Caculating PScore

xi: logit Intervention Age i.Sex i.Residence_type JKM_score i.Initial_symptom_group i.Days_onset_to_1stcode_cat i.ChestCTbefore1stcode i.Bronchoscopybefore1stcode i.Brainimagingbefore1stcode i.PETCTbefore1stcode i.Hist_final_groups

predict prob , pr

sum prob, d

*Generating atet weight

gen weight=1 if Intervention==1 & prob~=.

replace weight=prob/(1-prob) if Intervention==0

save "C:\DATA\OneDrive - SYREON Kft\Safe Syreon\syreon\horizon2020\selfie\elemzes\retro_tudo_plusz201910\data\retro_tudo_plus20200904_surv_weights_edited.dta", replace

*Rubins' B & R

qui sum prob if Intervention==1

scalar mi1 = r(mean)

scalar vi1 = r(Var)

qui sum prob if Intervention==0

scalar mi0 = r(mean)

scalar vi0 = r(Var)

qui sum prob [iweight=weight] if Intervention==1

scalar mi1_w = r(mean)

scalar vi1_w = r(Var)

qui sum prob [iweight=weight] if Intervention==0

scalar mi0_w = r(mean)

scalar vi0_w = r(Var)

*Rubins' B < 25

*B unmatched

display 100*(mi1 - mi0)/sqrt((vi1 + vi0)/2)

*B matched

di 100*(mi1_w - mi0_w)/sqrt((vi1_w + vi0_w)/2)

*Rubins' R > 0.5 & < 2

di "R unmatched is " vi1/vi0

di "R matched is "vi1_w/vi0_w

*Weighted Cox-regression

stset fuptime [pweight = weight], failure(death)

xi: stcox i.Intervention Age i.Sex i.Residence_type JKM_score i.Initial_symptom_group i.Days_onset_to_1stcode_cat i.ChestCTbefore1stcode i.Bronchoscopybefore1stcode i.Brainimagingbefore1stcode i.PETCTbefore1stcode i.Hist_final_groups

xi: stcox i.Intervention

predict basesurv, basesurv

scalar def coef_interven=_b[_IIntervent_1]

*Generate effect at year 1

egen timepointindex=max(fuptime) if fuptime<=365

egen timepoint=min(timepointindex)

generate index=basesurv if fuptime==timepoint

egen basesurv2=min(index)

replace basesurv2=. if Intervention==0

gen isurv=basesurv2^exp(coef_interven) if Intervention==1 & weight~=.

gen cfsurv=basesurv2 if Intervention==1 & weight~=.

gen effect=isurv-cfsurv

sum isurv cfsurv effect, d

scalar drop coef_interven

preserve

use "c:\DATA\OneDrive - SYREON Kft\Safe Syreon\syreon\horizon2020\selfie\elemzes\retro_tudo_plusz201910\data\survival_bootstrap20200904.dta", clear

drop _all

save "c:\DATA\OneDrive - SYREON Kft\Safe Syreon\syreon\horizon2020\selfie\elemzes\retro_tudo_plusz201910\data\survival_bootstrap20200921_year1.dta", replace emptyok

restore

*Bootstraping

forvalues boot = 1/1000 {

preserve

quietly drop basesurv basesurv2 index timepointindex timepoint isurv cfsurv effect prob weight

set seed `boot'068

bsample

quietly xi: logit Intervention Age i.Sex i.Residence_type JKM_score i.Initial_symptom_group i.Days_onset_to_1stcode_cat i.ChestCTbefore1stcode i.Bronchoscopybefore1stcode i.Brainimagingbefore1stcode i.PETCTbefore1stcode i.Hist_final_groups

quietly predict prob , pr

*generating atet weight

quietly gen weight=1 if Intervention==1 & prob~=.

quietly replace weight=prob/(1-prob) if Intervention==0

*Cox-regression

quietly xi: stcox i.Intervention

quietly predict basesurv, basesurv

scalar def coef_interven=_b[_IIntervent_1]

quietly egen timepointindex=max(fuptime) if fuptime<=365

quietly egen timepoint=min(timepointindex)

quietly generate index=basesurv if fuptime==timepoint

quietly egen basesurv2=min(index)

quietly replace basesurv2=. if Intervention==0

quietly gen isurv=basesurv2^exp(coef_interven) if Intervention==1 & weight~=.

quietly gen cfsurv=basesurv2 if Intervention==1 & weight~=.

quietly gen effect=isurv-cfsurv

quietly sum isurv ,d

quietly gen isurv_mean = r(mean)

quietly sum cfsurv ,d

quietly gen cfsurv_mean = r(mean)

quietly sum effect ,d

quietly gen effect_mean = r(mean)

scalar drop coef_interven

quietly keep in 1

quietly keep isurv_mean cfsurv_mean effect_mean

quietly append using "c:\DATA\OneDrive - SYREON Kft\Safe Syreon\syreon\horizon2020\selfie\elemzes\retro_tudo_plusz201910\data\survival_bootstrap20200921_year1.dta"

quietly save "c:\DATA\OneDrive - SYREON Kft\Safe Syreon\syreon\horizon2020\selfie\elemzes\retro_tudo_plusz201910\data\survival_bootstrap20200921_year1.dta", replace

restore

}

use "c:\DATA\OneDrive - SYREON Kft\Safe Syreon\syreon\horizon2020\selfie\elemzes\retro_tudo_plusz201910\data\survival_bootstrap20200921_year1.dta", clear

save "c:\DATA\OneDrive - SYREON Kft\Safe Syreon\syreon\horizon2020\selfie\elemzes\retro_tudo_plusz201910\data\survival_bootstrap20200921_year1_arch.dta", replace

sum isurv_mean cfsurv_mean effect_mean, d

centile isurv_mean cfsurv_mean effect_mean, centile(2.5 97.5)

*Generate effect at year 2

use "C:\DATA\OneDrive - SYREON Kft\Safe Syreon\syreon\horizon2020\selfie\elemzes\retro_tudo_plusz201910\data\retro_tudo_plus20200904_surv_weights_edited.dta", clear

stset fuptime [pweight = weight], failure(death)

xi: stcox i.Intervention Age i.Sex i.Residence_type JKM_score i.Initial_symptom_group i.Days_onset_to_1stcode_cat i.ChestCTbefore1stcode i.Bronchoscopybefore1stcode i.Brainimagingbefore1stcode i.PETCTbefore1stcode i.Hist_final_groups

xi: stcox i.Intervention

predict basesurv, basesurv

scalar def coef_interven=_b[_IIntervent_1]

egen timepointindex=max(fuptime) if fuptime<=730

egen timepoint=min(timepointindex)

generate index=basesurv if fuptime==timepoint

egen basesurv2=min(index)

replace basesurv2=. if Intervention==0

gen isurv=basesurv2^exp(coef_interven) if Intervention==1 & weight~=.

gen cfsurv=basesurv2 if Intervention==1 & weight~=.

gen effect=isurv-cfsurv

sum isurv cfsurv effect, d

scalar drop coef_interven

preserve

use "c:\DATA\OneDrive - SYREON Kft\Safe Syreon\syreon\horizon2020\selfie\elemzes\retro_tudo_plusz201910\data\survival_bootstrap20200904.dta", clear

drop _all

save "c:\DATA\OneDrive - SYREON Kft\Safe Syreon\syreon\horizon2020\selfie\elemzes\retro_tudo_plusz201910\data\survival_bootstrap20200921_year2.dta", replace emptyok

restore

*Bootstraping

forvalues boot = 1/1000 {

preserve

quietly drop basesurv basesurv2 index timepointindex timepoint isurv cfsurv effect prob weight

set seed `boot'078

bsample

quietly xi: logit Intervention Age i.Sex i.Residence_type JKM_score i.Initial_symptom_group i.Days_onset_to_1stcode_cat i.ChestCTbefore1stcode i.Bronchoscopybefore1stcode i.Brainimagingbefore1stcode i.PETCTbefore1stcode i.Hist_final_groups

quietly predict prob , pr

*generating atet weight

quietly gen weight=1 if Intervention==1 & prob~=.

quietly replace weight=prob/(1-prob) if Intervention==0

*Cox-regression

quietly xi: stcox i.Intervention

quietly predict basesurv, basesurv

scalar def coef_interven=_b[_IIntervent_1]

quietly egen timepointindex=max(fuptime) if fuptime<=730

quietly egen timepoint=min(timepointindex)

quietly generate index=basesurv if fuptime==timepoint

quietly egen basesurv2=min(index)

quietly replace basesurv2=. if Intervention==0

quietly gen isurv=basesurv2^exp(coef_interven) if Intervention==1 & weight~=.

quietly gen cfsurv=basesurv2 if Intervention==1 & weight~=.

quietly gen effect=isurv-cfsurv

quietly sum isurv ,d

quietly gen isurv_mean = r(mean)

quietly sum cfsurv ,d

quietly gen cfsurv_mean = r(mean)

quietly sum effect ,d

quietly gen effect_mean = r(mean)

scalar drop coef_interven

quietly keep in 1

quietly keep isurv_mean cfsurv_mean effect_mean

quietly append using "c:\DATA\OneDrive - SYREON Kft\Safe Syreon\syreon\horizon2020\selfie\elemzes\retro_tudo_plusz201910\data\survival_bootstrap20200921_year2.dta"

quietly save "c:\DATA\OneDrive - SYREON Kft\Safe Syreon\syreon\horizon2020\selfie\elemzes\retro_tudo_plusz201910\data\survival_bootstrap20200921_year2.dta", replace

restore

}

use "c:\DATA\OneDrive - SYREON Kft\Safe Syreon\syreon\horizon2020\selfie\elemzes\retro_tudo_plusz201910\data\survival_bootstrap20200921_year2.dta", clear

save "c:\DATA\OneDrive - SYREON Kft\Safe Syreon\syreon\horizon2020\selfie\elemzes\retro_tudo_plusz201910\data\survival_bootstrap20200921_year2_arch.dta", replace

sum isurv_mean cfsurv_mean effect_mean, d

centile isurv_mean cfsurv_mean effect_mean, centile(2.5 97.5)

*Generate effect at year 3

use "C:\DATA\OneDrive - SYREON Kft\Safe Syreon\syreon\horizon2020\selfie\elemzes\retro_tudo_plusz201910\data\retro_tudo_plus20200904_surv_weights_edited.dta", clear

stset fuptime [pweight = weight], failure(death)

xi: stcox i.Intervention Age i.Sex i.Residence_type JKM_score i.Initial_symptom_group i.Days_onset_to_1stcode_cat i.ChestCTbefore1stcode i.Bronchoscopybefore1stcode i.Brainimagingbefore1stcode i.PETCTbefore1stcode i.Hist_final_groups

xi: stcox i.Intervention

predict basesurv, basesurv

scalar def coef_interven=_b[_IIntervent_1]

egen timepointindex=max(fuptime) if fuptime<=1095

egen timepoint=min(timepointindex)

generate index=basesurv if fuptime==timepoint

egen basesurv2=min(index)

replace basesurv2=. if Intervention==0

gen isurv=basesurv2^exp(coef_interven) if Intervention==1 & weight~=.

gen cfsurv=basesurv2 if Intervention==1 & weight~=.

gen effect=isurv-cfsurv

sum isurv cfsurv effect, d

scalar drop coef_interven

preserve

use "c:\DATA\OneDrive - SYREON Kft\Safe Syreon\syreon\horizon2020\selfie\elemzes\retro_tudo_plusz201910\data\survival_bootstrap20200904.dta", clear

drop _all

save "c:\DATA\OneDrive - SYREON Kft\Safe Syreon\syreon\horizon2020\selfie\elemzes\retro_tudo_plusz201910\data\survival_bootstrap20200921_year3.dta", replace emptyok

restore

*Bootstraping

forvalues boot = 1/1000 {

preserve

quietly drop basesurv basesurv2 index timepointindex timepoint isurv cfsurv effect prob weight

set seed `boot'112

bsample

quietly xi: logit Intervention Age i.Sex i.Residence_type JKM_score i.Initial_symptom_group i.Days_onset_to_1stcode_cat i.ChestCTbefore1stcode i.Bronchoscopybefore1stcode i.Brainimagingbefore1stcode i.PETCTbefore1stcode i.Hist_final_groups

quietly predict prob , pr

*generating atet weight

quietly gen weight=1 if Intervention==1 & prob~=.

quietly replace weight=prob/(1-prob) if Intervention==0

*Cox-regression

quietly xi: stcox i.Intervention

quietly predict basesurv, basesurv

scalar def coef_interven=_b[_IIntervent_1]

quietly egen timepointindex=max(fuptime) if fuptime<=1095

quietly egen timepoint=min(timepointindex)

quietly generate index=basesurv if fuptime==timepoint

quietly egen basesurv2=min(index)

quietly replace basesurv2=. if Intervention==0

quietly gen isurv=basesurv2^exp(coef_interven) if Intervention==1 & weight~=.

quietly gen cfsurv=basesurv2 if Intervention==1 & weight~=.

quietly gen effect=isurv-cfsurv

quietly sum isurv ,d

quietly gen isurv_mean = r(mean)

quietly sum cfsurv ,d

quietly gen cfsurv_mean = r(mean)

quietly sum effect ,d

quietly gen effect_mean = r(mean)

scalar drop coef_interven

quietly keep in 1

quietly keep isurv_mean cfsurv_mean effect_mean

quietly append using "c:\DATA\OneDrive - SYREON Kft\Safe Syreon\syreon\horizon2020\selfie\elemzes\retro_tudo_plusz201910\data\survival_bootstrap20200921_year3.dta"

quietly save "c:\DATA\OneDrive - SYREON Kft\Safe Syreon\syreon\horizon2020\selfie\elemzes\retro_tudo_plusz201910\data\survival_bootstrap20200921_year3.dta", replace

restore

}

use "c:\DATA\OneDrive - SYREON Kft\Safe Syreon\syreon\horizon2020\selfie\elemzes\retro_tudo_plusz201910\data\survival_bootstrap20200921_year3.dta", clear

save "c:\DATA\OneDrive - SYREON Kft\Safe Syreon\syreon\horizon2020\selfie\elemzes\retro_tudo_plusz201910\data\survival_bootstrap20200921_year3_arch.dta", replace

sum isurv_mean cfsurv_mean effect_mean, d

centile isurv_mean cfsurv_mean effect_mean, centile(2.5 97.5)

*nulla feletti bootstrap minták

use "c:\DATA\OneDrive - SYREON Kft\Safe Syreon\syreon\horizon2020\selfie\elemzes\retro_tudo_plusz201910\data\survival_bootstrap20200921_year1_arch.dta", clear

count if effect_mean>0 & effect_mean~=.

use "c:\DATA\OneDrive - SYREON Kft\Safe Syreon\syreon\horizon2020\selfie\elemzes\retro_tudo_plusz201910\data\survival_bootstrap20200921_year2_arch.dta", clear

count if effect_mean>0 & effect_mean~=.

use "c:\DATA\OneDrive - SYREON Kft\Safe Syreon\syreon\horizon2020\selfie\elemzes\retro_tudo_plusz201910\data\survival_bootstrap20200921_year3_arch.dta", clear

count if effect_mean>0 & effect_mean~=.

clear

log close

-------------------------------------------------------------------------------------

Stata LOG file:

name: <unnamed>

log: c:\DATA\OneDrive - SYREON Kft\Safe Syreon\syreon\horizon2020\selfie\elemzes\retro_tudo_plusz20191

> 0\selfie_retro_pulmo_plus20200924.log

log type: text

opened on: 24 Sep 2020, 15:34:42

. *selfie_retro_pulmo_plus20200924.do

. set more off

. clear

. import excel "c:\DATA\OneDrive - SYREON Kft\Safe Syreon\syreon\horizon2020\selfie\elemzes\retro_tudo_plusz2

> 01910\data\pulmo_retro20200904.xlsx" , sheet("maiadat") firstrow

(149 vars, 296 obs)

. encode PTID, generate (PTID2)

. rename VS_event death

. rename Surv_from_1stcode fuptime

. insp death

death: VS_event Number of Observations

---------------- ---------------------------------------

Total Integers Nonintegers

| # Negative - - -

| # Zero 173 173 -

| # # Positive 123 123 -

| # # ----------- ----------- -----------

| # # Total 296 296 -

| # # Missing -

+---------------------- -----------

0 1 296

(2 unique values)

. insp fuptime

fuptime: Surv_from_1stcode Number of Observations

--------------------------- ---------------------------------------

Total Integers Nonintegers

| # Negative - - -

| # Zero - - -

| # Positive 296 296 -

| # ----------- ----------- -----------

| # Total 296 296 -

| # # # # # Missing -

+---------------------- -----------

33 1797 296

(More than 99 unique values)

.

.

. *SURVIVAL

.

. *Caculating PScore

. xi: logit Intervention Age i.Sex i.Residence_type JKM_score i.Initial_symptom_group i.Days_onset_to_1stcode_

> cat i.ChestCTbefore1stcode i.Bronchoscopybefore1stcode i.Brainimagingbefore1stcode i.PETCTbefore1stcode i.H

> ist_final_groups

i.Sex _ISex_1-2 (_ISex_1 for Sex==female omitted)

i.Residence_t~e _IResidence_1-2 (_IResidence_1 for Resi~e==rural omitted)

i.Initia~_group _IInitial_s_1-3 (_IInitial_s_1 for Ini..==asymptomatic finding omitted)

i.Days_onset~at _IDays_onse_1-5 (_IDays_onse_1 for Da~at==(13,36] omitted)

i.ChestCTbefo~e _IChestCTbe_1-2 (_IChestCTbe_1 for ChestCT~e==no omitted)

i.Bronchosco~de _IBronchosc_1-2 (_IBronchosc_1 for Bronch~de==no omitted)

i.Brainimagin~e _IBrainimag_1-2 (_IBrainimag_1 for Brainim~e==no omitted)

i.PETCTbefore~e _IPETCTbefo_1-2 (_IPETCTbefo_1 for PETCTbe~e==no omitted)

i.Hist_final_~s _IHist_fina_1-4 (_IHist_fina_1 for His~s==NSCLC other_nos omitted)

Iteration 0: log likelihood = -200.92828

Iteration 1: log likelihood = -185.38238

Iteration 2: log likelihood = -185.34912

Iteration 3: log likelihood = -185.34912

Logistic regression Number of obs = 296

LR chi2(17) = 31.16

Prob > chi2 = 0.0191

Log likelihood = -185.34912 Pseudo R2 = 0.0775

-------------------------------------------------------------------------------

Intervention | Coef. Std. Err. z P>|z| [95% Conf. Interval]

--------------+----------------------------------------------------------------

Age | .0022531 .014743 0.15 0.879 -.0266427 .0311489

_ISex_2 | -.4906499 .2623901 -1.87 0.061 -1.004925 .0236252

_IResidence_2 | .3708897 .2614238 1.42 0.156 -.1414916 .883271

JKM_score | -.000378 .0175006 -0.02 0.983 -.0346786 .0339227

_IInitial_s_2 | -.5646903 .8131122 -0.69 0.487 -2.158361 1.02898

_IInitial_s_3 | .177672 .2774854 0.64 0.522 -.3661894 .7215334

_IDays_onse_2 | .1744565 .3813333 0.46 0.647 -.572943 .9218561

_IDays_onse_3 | -.3873502 .383048 -1.01 0.312 -1.13811 .3634101

_IDays_onse_4 | -.4861041 .3836281 -1.27 0.205 -1.238001 .2657931

_IDays_onse_5 | -.1097056 .4961322 -0.22 0.825 -1.082107 .8626956

_IChestCTbe_2 | -.6400125 .2990996 -2.14 0.032 -1.226237 -.0537881

_IBronchosc_2 | 2.196699 .6741736 3.26 0.001 .8753432 3.518055

_IBrainimag_2 | -.35754 .3539922 -1.01 0.312 -1.051352 .3362719

_IPETCTbefo_2 | .2644651 .7899148 0.33 0.738 -1.283739 1.81267

_IHist_fina_2 | .5993299 .5358556 1.12 0.263 -.4509279 1.649588

_IHist_fina_3 | .526377 .7029041 0.75 0.454 -.8512898 1.904044

_IHist_fina_4 | .7538351 .5321758 1.42 0.157 -.2892104 1.796881

_cons | -.5701575 1.293747 -0.44 0.659 -3.105855 1.96554

-------------------------------------------------------------------------------

. predict prob , pr

. sum prob, d

Pr(Intervention)

-------------------------------------------------------------

Percentiles Smallest

1% .1599491 .0958442

5% .2018572 .1383123

10% .2379439 .1599491 Obs 296

25% .3055528 .171632 Sum of Wgt. 296

50% .3940491 Mean .4155405

Largest Std. Dev. .156931

75% .50159 .8754097

90% .6227319 .8756036 Variance .0246273

95% .7502356 .8978103 Skewness .9126804

99% .8756036 .9181801 Kurtosis 3.858998

. *Generating atet weight

. gen weight=1 if Intervention==1 & prob~=.

(173 missing values generated)

. replace weight=prob/(1-prob) if Intervention==0

(173 real changes made)

. save "C:\DATA\OneDrive - SYREON Kft\Safe Syreon\syreon\horizon2020\selfie\elemzes\retro_tudo_plusz201910\da

> ta\retro_tudo_plus20200904_surv_weights_edited.dta", replace

file C:\DATA\OneDrive - SYREON Kft\Safe Syreon\syreon\horizon2020\selfie\elemzes\retro_tudo_plusz201910\data\r

> etro_tudo_plus20200904_surv_weights_edited.dta saved

.

. *Rubins' B & R

. qui sum prob if Intervention==1

. scalar mi1 = r(mean)

. scalar vi1 = r(Var)

. qui sum prob if Intervention==0

. scalar mi0 = r(mean)

. scalar vi0 = r(Var)

. qui sum prob [iweight=weight] if Intervention==1

. scalar mi1_w = r(mean)

. scalar vi1_w = r(Var)

. qui sum prob [iweight=weight] if Intervention==0

. scalar mi0_w = r(mean)

. scalar vi0_w = r(Var)

. *Rubins' B < 25

. *B unmatched

. display 100*(mi1 - mi0)/sqrt((vi1 + vi0)/2)

66.526005

. *B matched

. di 100*(mi1_w - mi0_w)/sqrt((vi1_w + vi0_w)/2)

9.2258406

. *Rubins' R > 0.5 & < 2

. di "R unmatched is " vi1/vi0

R unmatched is 1.8840382

. di "R matched is "vi1_w/vi0_w

R matched is 1.2944733

.

. *Weighted Cox-regression

. stset fuptime [pweight = weight], failure(death)

failure event: death != 0 & death < .

obs. time interval: (0, fuptime]

exit on or before: failure

weight: [pweight=weight]

------------------------------------------------------------------------------

296 total observations

0 exclusions

------------------------------------------------------------------------------

296 observations remaining, representing

123 failures in single-record/single-failure data

170,621 total analysis time at risk and under observation

at risk from t = 0

earliest observed entry t = 0

last observed exit t = 1,797

. xi: stcox i.Intervention Age i.Sex i.Residence_type JKM_score i.Initial_symptom_group i.Days_onset_to_1stcod

> e_cat i.ChestCTbefore1stcode i.Bronchoscopybefore1stcode i.Brainimagingbefore1stcode i.PETCTbefore1stcode i

> .Hist_final_groups

i.Intervention _IIntervent_0-1 (naturally coded; _IIntervent_0 omitted)

i.Sex _ISex_1-2 (_ISex_1 for Sex==female omitted)

i.Residence_t~e _IResidence_1-2 (_IResidence_1 for Resi~e==rural omitted)

i.Initia~_group _IInitial_s_1-3 (_IInitial_s_1 for Ini..==asymptomatic finding omitted)

i.Days_onset~at _IDays_onse_1-5 (_IDays_onse_1 for Da~at==(13,36] omitted)

i.ChestCTbefo~e _IChestCTbe_1-2 (_IChestCTbe_1 for ChestCT~e==no omitted)

i.Bronchosco~de _IBronchosc_1-2 (_IBronchosc_1 for Bronch~de==no omitted)

i.Brainimagin~e _IBrainimag_1-2 (_IBrainimag_1 for Brainim~e==no omitted)

i.PETCTbefore~e _IPETCTbefo_1-2 (_IPETCTbefo_1 for PETCTbe~e==no omitted)

i.Hist_final_~s _IHist_fina_1-4 (_IHist_fina_1 for His~s==NSCLC other_nos omitted)

failure _d: death

analysis time _t: fuptime

weight: [pweight=weight]

(sum of wgt is 242.5420639514923)

Iteration 0: log pseudolikelihood = -652.47329

Iteration 1: log pseudolikelihood = -632.15311

Iteration 2: log pseudolikelihood = -631.66715

Iteration 3: log pseudolikelihood = -631.65968

Iteration 4: log pseudolikelihood = -631.65967

Refining estimates:

Iteration 0: log pseudolikelihood = -631.65967

Cox regression -- Breslow method for ties

No. of subjects = 243 Number of obs = 296

No. of failures = 104

Time at risk = 133177.0343

Wald chi2(18) = 46.04

Log pseudolikelihood = -631.65967 Prob > chi2 = 0.0003

-------------------------------------------------------------------------------

| Robust

_t | Haz. Ratio Std. Err. z P>|z| [95% Conf. Interval]

--------------+----------------------------------------------------------------

_IIntervent_1 | .6343281 .1398126 -2.07 0.039 .4118132 .9770745

Age | 1.021776 .0127613 1.72 0.085 .9970675 1.047096

_ISex_2 | .9781508 .2080114 -0.10 0.917 .6447497 1.483954

_IResidence_2 | 1.355715 .2692507 1.53 0.125 .9185786 2.000878

JKM_score | 1.011873 .0159777 0.75 0.455 .9810367 1.043678

_IInitial_s_2 | 2.488784 1.497845 1.52 0.130 .7650709 8.096042

_IInitial_s_3 | 1.817659 .4512903 2.41 0.016 1.117314 2.956988

_IDays_onse_2 | .9490133 .2571432 -0.19 0.847 .5579972 1.614034

_IDays_onse_3 | .7379581 .234036 -0.96 0.338 .3963526 1.373984

_IDays_onse_4 | .983338 .3057757 -0.05 0.957 .5345839 1.808797

_IDays_onse_5 | .6634424 .2633534 -1.03 0.301 .3047325 1.4444

_IChestCTbe_2 | .57278 .1275294 -2.50 0.012 .3702269 .8861509

_IBronchosc_2 | 1.182799 .8134578 0.24 0.807 .3072598 4.553193

_IBrainimag_2 | 1.386593 .415896 1.09 0.276 .7702624 2.496084

_IPETCTbefo_2 | .3989819 .4754258 -0.77 0.441 .0386068 4.12328

_IHist_fina_2 | .9087432 .3876759 -0.22 0.823 .3938337 2.09686

_IHist_fina_3 | .7342714 .3922844 -0.58 0.563 .2576928 2.092237

_IHist_fina_4 | .9382123 .3726806 -0.16 0.872 .4307084 2.043709

-------------------------------------------------------------------------------

. xi: stcox i.Intervention

i.Intervention _IIntervent_0-1 (naturally coded; _IIntervent_0 omitted)

failure _d: death

analysis time _t: fuptime

weight: [pweight=weight]

(sum of wgt is 242.5420639514923)

Iteration 0: log pseudolikelihood = -652.47329

Iteration 1: log pseudolikelihood = -649.37479

Iteration 2: log pseudolikelihood = -649.37456

Refining estimates:

Iteration 0: log pseudolikelihood = -649.37456

Cox regression -- Breslow method for ties

No. of subjects = 243 Number of obs = 296

No. of failures = 104

Time at risk = 133177.0343

Wald chi2(1) = 4.80

Log pseudolikelihood = -649.37456 Prob > chi2 = 0.0285

-------------------------------------------------------------------------------

| Robust

_t | Haz. Ratio Std. Err. z P>|z| [95% Conf. Interval]

--------------+----------------------------------------------------------------

_IIntervent_1 | .6390191 .1306293 -2.19 0.028 .4280652 .9539327

-------------------------------------------------------------------------------

. predict basesurv, basesurv

. scalar def coef_interven=_b[_IIntervent_1]

.

. *Generate effect at year 1

. egen timepointindex=max(fuptime) if fuptime<=365

(142 missing values generated)

. egen timepoint=min(timepointindex)

. generate index=basesurv if fuptime==timepoint

(295 missing values generated)

. egen basesurv2=min(index)

. replace basesurv2=. if Intervention==0

(173 real changes made, 173 to missing)

. gen isurv=basesurv2^exp(coef_interven) if Intervention==1 & weight~=.

(173 missing values generated)

. gen cfsurv=basesurv2 if Intervention==1 & weight~=.

(173 missing values generated)

. gen effect=isurv-cfsurv

(173 missing values generated)

. sum isurv cfsurv effect, d

isurv

-------------------------------------------------------------

Percentiles Smallest

1% .7161011 .7161011

5% .7161011 .7161011

10% .7161011 .7161011 Obs 123

25% .7161011 .7161011 Sum of Wgt. 123

50% .7161011 Mean .7161011

Largest Std. Dev. 0

75% .7161011 .7161011

90% .7161011 .7161011 Variance 0

95% .7161011 .7161011 Skewness .

99% .7161011 .7161011 Kurtosis .

cfsurv

-------------------------------------------------------------

Percentiles Smallest

1% .592993 .592993

5% .592993 .592993

10% .592993 .592993 Obs 123

25% .592993 .592993 Sum of Wgt. 123

50% .592993 Mean .592993

Largest Std. Dev. 0

75% .592993 .592993

90% .592993 .592993 Variance 0

95% .592993 .592993 Skewness .

99% .592993 .592993 Kurtosis .

effect

-------------------------------------------------------------

Percentiles Smallest

1% .1231081 .1231081

5% .1231081 .1231081

10% .1231081 .1231081 Obs 123

25% .1231081 .1231081 Sum of Wgt. 123

50% .1231081 Mean .1231081

Largest Std. Dev. 0

75% .1231081 .1231081

90% .1231081 .1231081 Variance 0

95% .1231081 .1231081 Skewness .

99% .1231081 .1231081 Kurtosis .

. scalar drop coef_interven

. preserve

. use "c:\DATA\OneDrive - SYREON Kft\Safe Syreon\syreon\horizon2020\selfie\elemzes\retro_tudo_plusz201910\data

> \survival_bootstrap20200904.dta", clear

. drop _all

. save "c:\DATA\OneDrive - SYREON Kft\Safe Syreon\syreon\horizon2020\selfie\elemzes\retro_tudo_plusz201910\dat

> a\survival_bootstrap20200921_year1.dta", replace emptyok

(note: dataset contains 0 observations)

file c:\DATA\OneDrive - SYREON Kft\Safe Syreon\syreon\horizon2020\selfie\elemzes\retro_tudo_plusz201910\data\s

> urvival_bootstrap20200921_year1.dta saved

. restore

.

. *Bootstraping

. forvalues boot = 1/1000 {

2. preserve

3. quietly drop basesurv basesurv2 index timepointindex timepoint isurv cfsurv effect prob weight

4. set seed `boot'068

5. bsample

6. quietly xi: logit Intervention Age i.Sex i.Residence_type JKM_score i.Initial_symptom_group i.Days_onset_

> to_1stcode_cat i.ChestCTbefore1stcode i.Bronchoscopybefore1stcode i.Brainimagingbefore1stcode i.PETCTbefore1

> stcode i.Hist_final_groups

7. quietly predict prob , pr

8. *generating atet weight

. quietly gen weight=1 if Intervention==1 & prob~=.

9. quietly replace weight=prob/(1-prob) if Intervention==0

10. *Cox-regression

. quietly xi: stcox i.Intervention

11. quietly predict basesurv, basesurv

12. scalar def coef_interven=_b[_IIntervent_1]

13. quietly egen timepointindex=max(fuptime) if fuptime<=365

14. quietly egen timepoint=min(timepointindex)

15. quietly generate index=basesurv if fuptime==timepoint

16. quietly egen basesurv2=min(index)

17. quietly replace basesurv2=. if Intervention==0

18. quietly gen isurv=basesurv2^exp(coef_interven) if Intervention==1 & weight~=.

19. quietly gen cfsurv=basesurv2 if Intervention==1 & weight~=.

20. quietly gen effect=isurv-cfsurv

21. quietly sum isurv ,d

22. quietly gen isurv_mean = r(mean)

23. quietly sum cfsurv ,d

24. quietly gen cfsurv_mean = r(mean)

25. quietly sum effect ,d

26. quietly gen effect_mean = r(mean)

27. scalar drop coef_interven

28. quietly keep in 1

29. quietly keep isurv_mean cfsurv_mean effect_mean

30. quietly append using "c:\DATA\OneDrive - SYREON Kft\Safe Syreon\syreon\horizon2020\selfie\elemzes\retro_t

> udo_plusz201910\data\survival_bootstrap20200921_year1.dta"

31. quietly save "c:\DATA\OneDrive - SYREON Kft\Safe Syreon\syreon\horizon2020\selfie\elemzes\retro_tudo_plus

> z201910\data\survival_bootstrap20200921_year1.dta", replace

32. restore

33. }

.

. use "c:\DATA\OneDrive - SYREON Kft\Safe Syreon\syreon\horizon2020\selfie\elemzes\retro_tudo_plusz201910\data

> \survival_bootstrap20200921_year1.dta", clear

. save "c:\DATA\OneDrive - SYREON Kft\Safe Syreon\syreon\horizon2020\selfie\elemzes\retro_tudo_plusz201910\dat

> a\survival_bootstrap20200921_year1_arch.dta", replace

file c:\DATA\OneDrive - SYREON Kft\Safe Syreon\syreon\horizon2020\selfie\elemzes\retro_tudo_plusz201910\data\s

> urvival_bootstrap20200921_year1_arch.dta saved

. sum isurv_mean cfsurv_mean effect_mean, d

isurv_mean

-------------------------------------------------------------

Percentiles Smallest

1% .6149908 .5624175

5% .6459591 .5949938

10% .6630927 .6013806 Obs 1,000

25% .6866821 .6038116 Sum of Wgt. 1,000

50% .7153435 Mean .7165597

Largest Std. Dev. .0433292

75% .7468561 .8286299

90% .7725652 .8399889 Variance .0018774

95% .7891522 .8432882 Skewness .0098553

99% .8160582 .8606527 Kurtosis 2.935997

cfsurv_mean

-------------------------------------------------------------

Percentiles Smallest

1% .4643497 .3529993

5% .494968 .4266556

10% .5181392 .4287484 Obs 1,000

25% .5535074 .4349349 Sum of Wgt. 1,000

50% .5929554 Mean .5924816

Largest Std. Dev. .0578495

75% .6317378 .7622067

90% .6639193 .7666117 Variance .0033466

95% .6856521 .7669444 Skewness -.0482522

99% .7283325 .7797163 Kurtosis 3.159648

effect_mean

-------------------------------------------------------------

Percentiles Smallest

1% -.0165299 -.0712861

5% .0193139 -.0482835

10% .0440223 -.0426698 Obs 1,000

25% .0813312 -.0396714 Sum of Wgt. 1,000

50% .1249889 Mean .1240781

Largest Std. Dev. .0629108

75% .1651433 .2930715

90% .2040321 .2931517 Variance .0039578

95% .2269116 .2939163 Skewness .0747571

99% .2808682 .3136367 Kurtosis 2.950639

. centile isurv_mean cfsurv_mean effect_mean, centile(2.5 97.5)

-- Binom. Interp. --

Variable | Obs Percentile Centile [95% Conf. Interval]

-------------+-------------------------------------------------------------

isurv_mean | 1,000 2.5 .6329914 .6253761 .6375885

| 97.5 .7991924 .7948658 .8077945

cfsurv_mean | 1,000 2.5 .4750066 .469559 .484599

| 97.5 .7064722 .6973467 .7179021

effect_mean | 1,000 2.5 .0016093 -.006931 .0102472

| 97.5 .249068 .2433161 .2675653

.

.

.

. *Generate effect at year 2

. use "C:\DATA\OneDrive - SYREON Kft\Safe Syreon\syreon\horizon2020\selfie\elemzes\retro_tudo_plusz201910\data

> \retro_tudo_plus20200904_surv_weights_edited.dta", clear

. stset fuptime [pweight = weight], failure(death)

failure event: death != 0 & death < .

obs. time interval: (0, fuptime]

exit on or before: failure

weight: [pweight=weight]

------------------------------------------------------------------------------

296 total observations

0 exclusions

------------------------------------------------------------------------------

296 observations remaining, representing

123 failures in single-record/single-failure data

170,621 total analysis time at risk and under observation

at risk from t = 0

earliest observed entry t = 0

last observed exit t = 1,797

. xi: stcox i.Intervention Age i.Sex i.Residence_type JKM_score i.Initial_symptom_group i.Days_onset_to_1stcod

> e_cat i.ChestCTbefore1stcode i.Bronchoscopybefore1stcode i.Brainimagingbefore1stcode i.PETCTbefore1stcode i

> .Hist_final_groups

i.Intervention _IIntervent_0-1 (naturally coded; _IIntervent_0 omitted)

i.Sex _ISex_1-2 (_ISex_1 for Sex==female omitted)

i.Residence_t~e _IResidence_1-2 (_IResidence_1 for Resi~e==rural omitted)

i.Initia~_group _IInitial_s_1-3 (_IInitial_s_1 for Ini..==asymptomatic finding omitted)

i.Days_onset~at _IDays_onse_1-5 (_IDays_onse_1 for Da~at==(13,36] omitted)

i.ChestCTbefo~e _IChestCTbe_1-2 (_IChestCTbe_1 for ChestCT~e==no omitted)

i.Bronchosco~de _IBronchosc_1-2 (_IBronchosc_1 for Bronch~de==no omitted)

i.Brainimagin~e _IBrainimag_1-2 (_IBrainimag_1 for Brainim~e==no omitted)

i.PETCTbefore~e _IPETCTbefo_1-2 (_IPETCTbefo_1 for PETCTbe~e==no omitted)

i.Hist_final_~s _IHist_fina_1-4 (_IHist_fina_1 for His~s==NSCLC other_nos omitted)

failure _d: death

analysis time _t: fuptime

weight: [pweight=weight]

(sum of wgt is 242.5420639514923)

Iteration 0: log pseudolikelihood = -652.47329

Iteration 1: log pseudolikelihood = -632.15311

Iteration 2: log pseudolikelihood = -631.66715

Iteration 3: log pseudolikelihood = -631.65968

Iteration 4: log pseudolikelihood = -631.65967

Refining estimates:

Iteration 0: log pseudolikelihood = -631.65967

Cox regression -- Breslow method for ties

No. of subjects = 243 Number of obs = 296

No. of failures = 104

Time at risk = 133177.0343

Wald chi2(18) = 46.04

Log pseudolikelihood = -631.65967 Prob > chi2 = 0.0003

-------------------------------------------------------------------------------

| Robust

_t | Haz. Ratio Std. Err. z P>|z| [95% Conf. Interval]

--------------+----------------------------------------------------------------

_IIntervent_1 | .6343281 .1398126 -2.07 0.039 .4118132 .9770745

Age | 1.021776 .0127613 1.72 0.085 .9970675 1.047096

_ISex_2 | .9781508 .2080114 -0.10 0.917 .6447497 1.483954

_IResidence_2 | 1.355715 .2692507 1.53 0.125 .9185786 2.000878

JKM_score | 1.011873 .0159777 0.75 0.455 .9810367 1.043678

_IInitial_s_2 | 2.488784 1.497845 1.52 0.130 .7650709 8.096042

_IInitial_s_3 | 1.817659 .4512903 2.41 0.016 1.117314 2.956988

_IDays_onse_2 | .9490133 .2571432 -0.19 0.847 .5579972 1.614034

_IDays_onse_3 | .7379581 .234036 -0.96 0.338 .3963526 1.373984

_IDays_onse_4 | .983338 .3057757 -0.05 0.957 .5345839 1.808797

_IDays_onse_5 | .6634424 .2633534 -1.03 0.301 .3047325 1.4444

_IChestCTbe_2 | .57278 .1275294 -2.50 0.012 .3702269 .8861509

_IBronchosc_2 | 1.182799 .8134578 0.24 0.807 .3072598 4.553193

_IBrainimag_2 | 1.386593 .415896 1.09 0.276 .7702624 2.496084

_IPETCTbefo_2 | .3989819 .4754258 -0.77 0.441 .0386068 4.12328

_IHist_fina_2 | .9087432 .3876759 -0.22 0.823 .3938337 2.09686

_IHist_fina_3 | .7342714 .3922844 -0.58 0.563 .2576928 2.092237

_IHist_fina_4 | .9382123 .3726806 -0.16 0.872 .4307084 2.043709

-------------------------------------------------------------------------------

. xi: stcox i.Intervention

i.Intervention _IIntervent_0-1 (naturally coded; _IIntervent_0 omitted)

failure _d: death

analysis time _t: fuptime

weight: [pweight=weight]

(sum of wgt is 242.5420639514923)

Iteration 0: log pseudolikelihood = -652.47329

Iteration 1: log pseudolikelihood = -649.37479

Iteration 2: log pseudolikelihood = -649.37456

Refining estimates:

Iteration 0: log pseudolikelihood = -649.37456

Cox regression -- Breslow method for ties

No. of subjects = 243 Number of obs = 296

No. of failures = 104

Time at risk = 133177.0343

Wald chi2(1) = 4.80

Log pseudolikelihood = -649.37456 Prob > chi2 = 0.0285

-------------------------------------------------------------------------------

| Robust

_t | Haz. Ratio Std. Err. z P>|z| [95% Conf. Interval]

--------------+----------------------------------------------------------------

_IIntervent_1 | .6390191 .1306293 -2.19 0.028 .4280652 .9539327

-------------------------------------------------------------------------------

. predict basesurv, basesurv

. scalar def coef_interven=_b[_IIntervent_1]

. egen timepointindex=max(fuptime) if fuptime<=730

(102 missing values generated)

. egen timepoint=min(timepointindex)

. generate index=basesurv if fuptime==timepoint

(295 missing values generated)

. egen basesurv2=min(index)

. replace basesurv2=. if Intervention==0

(173 real changes made, 173 to missing)

. gen isurv=basesurv2^exp(coef_interven) if Intervention==1 & weight~=.

(173 missing values generated)

. gen cfsurv=basesurv2 if Intervention==1 & weight~=.

(173 missing values generated)

. gen effect=isurv-cfsurv

(173 missing values generated)

. sum isurv cfsurv effect, d

isurv

-------------------------------------------------------------

Percentiles Smallest

1% .5951481 .5951481

5% .5951481 .5951481

10% .5951481 .5951481 Obs 123

25% .5951481 .5951481 Sum of Wgt. 123

50% .5951481 Mean .5951481

Largest Std. Dev. 0

75% .5951481 .5951481

90% .5951481 .5951481 Variance 0

95% .5951481 .5951481 Skewness .

99% .5951481 .5951481 Kurtosis .

cfsurv

-------------------------------------------------------------

Percentiles Smallest

1% .4439265 .4439265

5% .4439265 .4439265

10% .4439265 .4439265 Obs 123

25% .4439265 .4439265 Sum of Wgt. 123

50% .4439265 Mean .4439265

Largest Std. Dev. 0

75% .4439265 .4439265

90% .4439265 .4439265 Variance 0

95% .4439265 .4439265 Skewness .

99% .4439265 .4439265 Kurtosis .

effect

-------------------------------------------------------------

Percentiles Smallest

1% .1512216 .1512216

5% .1512216 .1512216

10% .1512216 .1512216 Obs 123

25% .1512216 .1512216 Sum of Wgt. 123

50% .1512216 Mean .1512216

Largest Std. Dev. 0

75% .1512216 .1512216

90% .1512216 .1512216 Variance 0

95% .1512216 .1512216 Skewness .

99% .1512216 .1512216 Kurtosis .

. scalar drop coef_interven

. preserve

. use "c:\DATA\OneDrive - SYREON Kft\Safe Syreon\syreon\horizon2020\selfie\elemzes\retro_tudo_plusz201910\data

> \survival_bootstrap20200904.dta", clear

. drop _all

. save "c:\DATA\OneDrive - SYREON Kft\Safe Syreon\syreon\horizon2020\selfie\elemzes\retro_tudo_plusz201910\dat

> a\survival_bootstrap20200921_year2.dta", replace emptyok

(note: dataset contains 0 observations)

file c:\DATA\OneDrive - SYREON Kft\Safe Syreon\syreon\horizon2020\selfie\elemzes\retro_tudo_plusz201910\data\s

> urvival_bootstrap20200921_year2.dta saved

. restore

.

. *Bootstraping

. forvalues boot = 1/1000 {

2. preserve

3. quietly drop basesurv basesurv2 index timepointindex timepoint isurv cfsurv effect prob weight

4. set seed `boot'078

5. bsample

6. quietly xi: logit Intervention Age i.Sex i.Residence_type JKM_score i.Initial_symptom_group i.Days_onset_

> to_1stcode_cat i.ChestCTbefore1stcode i.Bronchoscopybefore1stcode i.Brainimagingbefore1stcode i.PETCTbefore1

> stcode i.Hist_final_groups

7. quietly predict prob , pr

8. *generating atet weight

. quietly gen weight=1 if Intervention==1 & prob~=.

9. quietly replace weight=prob/(1-prob) if Intervention==0

10. *Cox-regression

. quietly xi: stcox i.Intervention

11. quietly predict basesurv, basesurv

12. scalar def coef_interven=_b[_IIntervent_1]

13. quietly egen timepointindex=max(fuptime) if fuptime<=730

14. quietly egen timepoint=min(timepointindex)

15. quietly generate index=basesurv if fuptime==timepoint

16. quietly egen basesurv2=min(index)

17. quietly replace basesurv2=. if Intervention==0

18. quietly gen isurv=basesurv2^exp(coef_interven) if Intervention==1 & weight~=.

19. quietly gen cfsurv=basesurv2 if Intervention==1 & weight~=.

20. quietly gen effect=isurv-cfsurv

21. quietly sum isurv ,d

22. quietly gen isurv_mean = r(mean)

23. quietly sum cfsurv ,d

24. quietly gen cfsurv_mean = r(mean)

25. quietly sum effect ,d

26. quietly gen effect_mean = r(mean)

27. scalar drop coef_interven

28. quietly keep in 1

29. quietly keep isurv_mean cfsurv_mean effect_mean

30. quietly append using "c:\DATA\OneDrive - SYREON Kft\Safe Syreon\syreon\horizon2020\selfie\elemzes\retro_t

> udo_plusz201910\data\survival_bootstrap20200921_year2.dta"

31. quietly save "c:\DATA\OneDrive - SYREON Kft\Safe Syreon\syreon\horizon2020\selfie\elemzes\retro_tudo_plus

> z201910\data\survival_bootstrap20200921_year2.dta", replace

32. restore

33. }

.

. use "c:\DATA\OneDrive - SYREON Kft\Safe Syreon\syreon\horizon2020\selfie\elemzes\retro_tudo_plusz201910\data

> \survival_bootstrap20200921_year2.dta", clear

. save "c:\DATA\OneDrive - SYREON Kft\Safe Syreon\syreon\horizon2020\selfie\elemzes\retro_tudo_plusz201910\dat

> a\survival_bootstrap20200921_year2_arch.dta", replace

file c:\DATA\OneDrive - SYREON Kft\Safe Syreon\syreon\horizon2020\selfie\elemzes\retro_tudo_plusz201910\data\s

> urvival_bootstrap20200921_year2_arch.dta saved

. sum isurv_mean cfsurv_mean effect_mean, d

isurv_mean

-------------------------------------------------------------

Percentiles Smallest

1% .4683553 .4136123

5% .5133174 .4165988

10% .5271121 .4388968 Obs 975

25% .5605842 .4431646 Sum of Wgt. 975

50% .5991575 Mean .5958418

Largest Std. Dev. .0505897

75% .6305094 .7104161

90% .6585909 .7135847 Variance .0025593

95% .6784304 .7189884 Skewness -.2598763

99% .702301 .7274858 Kurtosis 2.98042

cfsurv_mean

-------------------------------------------------------------

Percentiles Smallest

1% .3059511 .2547489

5% .3459488 .255805

10% .3638516 .2688905 Obs 975

25% .3994161 .2773674 Sum of Wgt. 975

50% .4365236 Mean .440785

Largest Std. Dev. .0620446

75% .4803355 .6259434

90% .519668 .6387474 Variance .0038495

95% .5466423 .6501067 Skewness .2448557

99% .6012914 .6561868 Kurtosis 3.229064

effect_mean

-------------------------------------------------------------

Percentiles Smallest

1% -.0301611 -.1057564

5% .0309798 -.0867787

10% .0556356 -.0746424 Obs 975

25% .1065978 -.0495992 Sum of Wgt. 975

50% .1582056 Mean .1550568

Largest Std. Dev. .0748394

75% .2040964 .351326

90% .2480586 .3574198 Variance .0056009

95% .2734244 .3599841 Skewness -.1627452

99% .3289204 .3668498 Kurtosis 3.132463

. centile isurv_mean cfsurv_mean effect_mean, centile(2.5 97.5)

-- Binom. Interp. --

Variable | Obs Percentile Centile [95% Conf. Interval]

-------------+-------------------------------------------------------------

isurv_mean | 975 2.5 .4948194 .479186 .5019271

| 97.5 .6903531 .6837518 .6949455

cfsurv_mean | 975 2.5 .3258602 .3124696 .3402583

| 97.5 .5784923 .5592152 .5885897

effect_mean | 975 2.5 -.0028188 -.0192199 .0110188

| 97.5 .3040201 .2847203 .3189023

.

. *Generate effect at year 3

. use "C:\DATA\OneDrive - SYREON Kft\Safe Syreon\syreon\horizon2020\selfie\elemzes\retro_tudo_plusz201910\data

> \retro_tudo_plus20200904_surv_weights_edited.dta", clear

. stset fuptime [pweight = weight], failure(death)

failure event: death != 0 & death < .

obs. time interval: (0, fuptime]

exit on or before: failure

weight: [pweight=weight]

------------------------------------------------------------------------------

296 total observations

0 exclusions

------------------------------------------------------------------------------

296 observations remaining, representing

123 failures in single-record/single-failure data

170,621 total analysis time at risk and under observation

at risk from t = 0

earliest observed entry t = 0

last observed exit t = 1,797

. xi: stcox i.Intervention Age i.Sex i.Residence_type JKM_score i.Initial_symptom_group i.Days_onset_to_1stcod

> e_cat i.ChestCTbefore1stcode i.Bronchoscopybefore1stcode i.Brainimagingbefore1stcode i.PETCTbefore1stcode i

> .Hist_final_groups

i.Intervention _IIntervent_0-1 (naturally coded; _IIntervent_0 omitted)

i.Sex _ISex_1-2 (_ISex_1 for Sex==female omitted)

i.Residence_t~e _IResidence_1-2 (_IResidence_1 for Resi~e==rural omitted)

i.Initia~_group _IInitial_s_1-3 (_IInitial_s_1 for Ini..==asymptomatic finding omitted)

i.Days_onset~at _IDays_onse_1-5 (_IDays_onse_1 for Da~at==(13,36] omitted)

i.ChestCTbefo~e _IChestCTbe_1-2 (_IChestCTbe_1 for ChestCT~e==no omitted)

i.Bronchosco~de _IBronchosc_1-2 (_IBronchosc_1 for Bronch~de==no omitted)

i.Brainimagin~e _IBrainimag_1-2 (_IBrainimag_1 for Brainim~e==no omitted)

i.PETCTbefore~e _IPETCTbefo_1-2 (_IPETCTbefo_1 for PETCTbe~e==no omitted)

i.Hist_final_~s _IHist_fina_1-4 (_IHist_fina_1 for His~s==NSCLC other_nos omitted)

failure _d: death

analysis time _t: fuptime

weight: [pweight=weight]

(sum of wgt is 242.5420639514923)

Iteration 0: log pseudolikelihood = -652.47329

Iteration 1: log pseudolikelihood = -632.15311

Iteration 2: log pseudolikelihood = -631.66715

Iteration 3: log pseudolikelihood = -631.65968

Iteration 4: log pseudolikelihood = -631.65967

Refining estimates:

Iteration 0: log pseudolikelihood = -631.65967

Cox regression -- Breslow method for ties

No. of subjects = 243 Number of obs = 296

No. of failures = 104

Time at risk = 133177.0343

Wald chi2(18) = 46.04

Log pseudolikelihood = -631.65967 Prob > chi2 = 0.0003

-------------------------------------------------------------------------------

| Robust

_t | Haz. Ratio Std. Err. z P>|z| [95% Conf. Interval]

--------------+----------------------------------------------------------------

_IIntervent_1 | .6343281 .1398126 -2.07 0.039 .4118132 .9770745

Age | 1.021776 .0127613 1.72 0.085 .9970675 1.047096

_ISex_2 | .9781508 .2080114 -0.10 0.917 .6447497 1.483954

_IResidence_2 | 1.355715 .2692507 1.53 0.125 .9185786 2.000878

JKM_score | 1.011873 .0159777 0.75 0.455 .9810367 1.043678

_IInitial_s_2 | 2.488784 1.497845 1.52 0.130 .7650709 8.096042

_IInitial_s_3 | 1.817659 .4512903 2.41 0.016 1.117314 2.956988

_IDays_onse_2 | .9490133 .2571432 -0.19 0.847 .5579972 1.614034

_IDays_onse_3 | .7379581 .234036 -0.96 0.338 .3963526 1.373984

_IDays_onse_4 | .983338 .3057757 -0.05 0.957 .5345839 1.808797

_IDays_onse_5 | .6634424 .2633534 -1.03 0.301 .3047325 1.4444

_IChestCTbe_2 | .57278 .1275294 -2.50 0.012 .3702269 .8861509

_IBronchosc_2 | 1.182799 .8134578 0.24 0.807 .3072598 4.553193

_IBrainimag_2 | 1.386593 .415896 1.09 0.276 .7702624 2.496084

_IPETCTbefo_2 | .3989819 .4754258 -0.77 0.441 .0386068 4.12328

_IHist_fina_2 | .9087432 .3876759 -0.22 0.823 .3938337 2.09686

_IHist_fina_3 | .7342714 .3922844 -0.58 0.563 .2576928 2.092237

_IHist_fina_4 | .9382123 .3726806 -0.16 0.872 .4307084 2.043709

-------------------------------------------------------------------------------

. xi: stcox i.Intervention

i.Intervention _IIntervent_0-1 (naturally coded; _IIntervent_0 omitted)

failure _d: death

analysis time _t: fuptime

weight: [pweight=weight]

(sum of wgt is 242.5420639514923)

Iteration 0: log pseudolikelihood = -652.47329

Iteration 1: log pseudolikelihood = -649.37479

Iteration 2: log pseudolikelihood = -649.37456

Refining estimates:

Iteration 0: log pseudolikelihood = -649.37456

Cox regression -- Breslow method for ties

No. of subjects = 243 Number of obs = 296

No. of failures = 104

Time at risk = 133177.0343

Wald chi2(1) = 4.80

Log pseudolikelihood = -649.37456 Prob > chi2 = 0.0285

-------------------------------------------------------------------------------

| Robust

_t | Haz. Ratio Std. Err. z P>|z| [95% Conf. Interval]

--------------+----------------------------------------------------------------

_IIntervent_1 | .6390191 .1306293 -2.19 0.028 .4280652 .9539327

-------------------------------------------------------------------------------

. predict basesurv, basesurv

. scalar def coef_interven=_b[_IIntervent_1]

. egen timepointindex=max(fuptime) if fuptime<=1095

(65 missing values generated)

. egen timepoint=min(timepointindex)

. generate index=basesurv if fuptime==timepoint

(295 missing values generated)

. egen basesurv2=min(index)

. replace basesurv2=. if Intervention==0

(173 real changes made, 173 to missing)

. gen isurv=basesurv2^exp(coef_interven) if Intervention==1 & weight~=.

(173 missing values generated)

. gen cfsurv=basesurv2 if Intervention==1 & weight~=.

(173 missing values generated)

. gen effect=isurv-cfsurv

(173 missing values generated)

. sum isurv cfsurv effect, d

isurv

-------------------------------------------------------------

Percentiles Smallest

1% .5422474 .5422474

5% .5422474 .5422474

10% .5422474 .5422474 Obs 123

25% .5422474 .5422474 Sum of Wgt. 123

50% .5422474 Mean .5422474

Largest Std. Dev. 0

75% .5422474 .5422474

90% .5422474 .5422474 Variance 0

95% .5422474 .5422474 Skewness .

99% .5422474 .5422474 Kurtosis .

cfsurv

-------------------------------------------------------------

Percentiles Smallest

1% .3837479 .3837479

5% .3837479 .3837479

10% .3837479 .3837479 Obs 123

25% .3837479 .3837479 Sum of Wgt. 123

50% .3837479 Mean .3837479

Largest Std. Dev. 0

75% .3837479 .3837479

90% .3837479 .3837479 Variance 0

95% .3837479 .3837479 Skewness .

99% .3837479 .3837479 Kurtosis .

effect

-------------------------------------------------------------

Percentiles Smallest

1% .1584995 .1584995

5% .1584995 .1584995

10% .1584995 .1584995 Obs 123

25% .1584995 .1584995 Sum of Wgt. 123

50% .1584995 Mean .1584995

Largest Std. Dev. 0

75% .1584995 .1584995

90% .1584995 .1584995 Variance 0

95% .1584995 .1584995 Skewness .

99% .1584995 .1584995 Kurtosis .

. scalar drop coef_interven

. preserve

. use "c:\DATA\OneDrive - SYREON Kft\Safe Syreon\syreon\horizon2020\selfie\elemzes\retro_tudo_plusz201910\data

> \survival_bootstrap20200904.dta", clear

. drop _all

. save "c:\DATA\OneDrive - SYREON Kft\Safe Syreon\syreon\horizon2020\selfie\elemzes\retro_tudo_plusz201910\dat

> a\survival_bootstrap20200921_year3.dta", replace emptyok

(note: dataset contains 0 observations)

file c:\DATA\OneDrive - SYREON Kft\Safe Syreon\syreon\horizon2020\selfie\elemzes\retro_tudo_plusz201910\data\s

> urvival_bootstrap20200921_year3.dta saved

. restore

.

. *Bootstraping

. forvalues boot = 1/1000 {

2. preserve

3. quietly drop basesurv basesurv2 index timepointindex timepoint isurv cfsurv effect prob weight

4. set seed `boot'112

5. bsample

6. quietly xi: logit Intervention Age i.Sex i.Residence_type JKM_score i.Initial_symptom_group i.Days_onset_

> to_1stcode_cat i.ChestCTbefore1stcode i.Bronchoscopybefore1stcode i.Brainimagingbefore1stcode i.PETCTbefore1

> stcode i.Hist_final_groups

7. quietly predict prob , pr

8. *generating atet weight

. quietly gen weight=1 if Intervention==1 & prob~=.

9. quietly replace weight=prob/(1-prob) if Intervention==0

10. *Cox-regression

. quietly xi: stcox i.Intervention

11. quietly predict basesurv, basesurv

12. scalar def coef_interven=_b[_IIntervent_1]

13. quietly egen timepointindex=max(fuptime) if fuptime<=1095

14. quietly egen timepoint=min(timepointindex)

15. quietly generate index=basesurv if fuptime==timepoint

16. quietly egen basesurv2=min(index)

17. quietly replace basesurv2=. if Intervention==0

18. quietly gen isurv=basesurv2^exp(coef_interven) if Intervention==1 & weight~=.

19. quietly gen cfsurv=basesurv2 if Intervention==1 & weight~=.

20. quietly gen effect=isurv-cfsurv

21. quietly sum isurv ,d

22. quietly gen isurv_mean = r(mean)

23. quietly sum cfsurv ,d

24. quietly gen cfsurv_mean = r(mean)

25. quietly sum effect ,d

26. quietly gen effect_mean = r(mean)

27. scalar drop coef_interven

28. quietly keep in 1

29. quietly keep isurv_mean cfsurv_mean effect_mean

30. quietly append using "c:\DATA\OneDrive - SYREON Kft\Safe Syreon\syreon\horizon2020\selfie\elemzes\retro_t

> udo_plusz201910\data\survival_bootstrap20200921_year3.dta"

31. quietly save "c:\DATA\OneDrive - SYREON Kft\Safe Syreon\syreon\horizon2020\selfie\elemzes\retro_tudo_plus

> z201910\data\survival_bootstrap20200921_year3.dta", replace

32. restore

33. }

.

. use "c:\DATA\OneDrive - SYREON Kft\Safe Syreon\syreon\horizon2020\selfie\elemzes\retro_tudo_plusz201910\data

> \survival_bootstrap20200921_year3.dta", clear

. save "c:\DATA\OneDrive - SYREON Kft\Safe Syreon\syreon\horizon2020\selfie\elemzes\retro_tudo_plusz201910\dat

> a\survival_bootstrap20200921_year3_arch.dta", replace

file c:\DATA\OneDrive - SYREON Kft\Safe Syreon\syreon\horizon2020\selfie\elemzes\retro_tudo_plusz201910\data\s

> urvival_bootstrap20200921_year3_arch.dta saved

. sum isurv_mean cfsurv_mean effect_mean, d

isurv_mean

-------------------------------------------------------------

Percentiles Smallest

1% .4203344 .3862664

5% .4510183 .3868244

10% .472589 .3880171 Obs 989

25% .5076656 .3886546 Sum of Wgt. 989

50% .5404792 Mean .5405801

Largest Std. Dev. .0526331

75% .5762512 .6854927

90% .6084105 .6950322 Variance .0027702

95% .6259891 .707899 Skewness -.0290948

99% .657611 .7120967 Kurtosis 3.0088

cfsurv_mean

-------------------------------------------------------------

Percentiles Smallest

1% .2242226 .1516709

5% .2756248 .1703693

10% .2981551 .1855569 Obs 989

25% .3392472 .1869396 Sum of Wgt. 989

50% .3807037 Mean .3810378

Largest Std. Dev. .0656484

75% .421552 .6072062

90% .462249 .6179251 Variance .0043097

95% .4885525 .6565761 Skewness .1801485

99% .5332106 .7037793 Kurtosis 3.944263

effect_mean

-------------------------------------------------------------

Percentiles Smallest

1% -.0326158 -.0823851

5% .0306417 -.0805516

10% .0560009 -.0720243 Obs 989

25% .1074943 -.0639326 Sum of Wgt. 989

50% .157045 Mean .1595422

Largest Std. Dev. .0790861

75% .215106 .3748775

90% .262218 .3772162 Variance .0062546

95% .2888573 .3781118 Skewness -.0835204

99% .3335513 .3909602 Kurtosis 2.953467

. centile isurv_mean cfsurv_mean effect_mean, centile(2.5 97.5)

-- Binom. Interp. --

Variable | Obs Percentile Centile [95% Conf. Interval]

-------------+-------------------------------------------------------------

isurv_mean | 989 2.5 .4369249 .4303142 .4411324

| 97.5 .6451311 .6354521 .6521698

cfsurv_mean | 989 2.5 .2574997 .2385189 .2676827

| 97.5 .5161813 .5052291 .525386

effect_mean | 989 2.5 -.0000653 -.0224433 .0101376

| 97.5 .3098311 .2971114 .3235817

.

. *nulla feletti bootstrap minták

. use "c:\DATA\OneDrive - SYREON Kft\Safe Syreon\syreon\horizon2020\selfie\elemzes\retro_tudo_plusz201910\data

> \survival_bootstrap20200921_year1_arch.dta", clear

. count if effect_mean>0 & effect_mean~=.

977

. use "c:\DATA\OneDrive - SYREON Kft\Safe Syreon\syreon\horizon2020\selfie\elemzes\retro_tudo_plusz201910\data

> \survival_bootstrap20200921_year2_arch.dta", clear

. count if effect_mean>0 & effect_mean~=.

950

. use "c:\DATA\OneDrive - SYREON Kft\Safe Syreon\syreon\horizon2020\selfie\elemzes\retro_tudo_plusz201910\data

> \survival_bootstrap20200921_year3_arch.dta", clear

. count if effect_mean>0 & effect_mean~=.

964

.

. clear

. log close

name: <unnamed>

log: c:\DATA\OneDrive - SYREON Kft\Safe Syreon\syreon\horizon2020\selfie\elemzes\retro_tudo_plusz20191

> 0\selfie_retro_pulmo_plus20200924.log

log type: text

closed on: 24 Sep 2020, 15:39:05

-------------------------------------------------------------------------------------
