# Supplementary material for: Improved survival of non-small cell lung cancer patients after introducing patient navigation: A retrospective cohort study with propensity score weighted historic control
Source: PLoS One. 2022 Oct 25;17(10):e0276719. doi: 10.1371/journal.pone.0276719 (PMC9595513; doi:10.1371/journal.pone.0276719)
Supplement: S4 File — MS Word file, with page numbers and relevant text from manuscript. (DOCX) [file pone.0276719.s005.docx]

STROBE Statement—checklist of items that should be included in reports of observational studies

|  | Item No. | Recommendation | Page  No. | Relevant text from manuscript |
| --- | --- | --- | --- | --- |
| **Title and abstract** | 1 | (*a*) Indicate the study’s design with a commonly used term in the title or the abstract | 1 | Retrospective cohort study with propensity score weighted historic control |
|  |  | (*b*) Provide in the abstract an informative and balanced summary of what was done and what was found | 2 | A retrospective cohort study was conducted, enrolling new cancer suspect patients with subsequently confirmed NSCLC in two annual periods, before and after OnkoNetwork implementation (control and intervention cohorts, respectively). To control for selection bias and confounding, baseline balance was improved via propensity score weighting. Overall survival was analyzed in univariate and multivariate weighted Cox regression models and the effect was further characterized in a counterfactual analysis. Our analysis included 123 intervention and 173 control NSCLC patients from early to advanced stage, with significant between-cohort baseline differences. The propensity score-based weighting resulted in good baseline balance. A large survival benefit was observed in the intervention cohort, and intervention was an independent predictor of longer survival in a multivariate analysis when all baseline characteristics were included (HR=0.63, p=0.039). When post-baseline variables were included in the model, belonging to the intervention cohort was not an independent predictor of survival, but the survival benefit was explained by the broader use of PET-CT in the diagnostic phase, higher resectability rate, and/or slightly less advanced cases with stage IV and ECOG 3-4 status at treatment initiation. |
| Introduction | | | |  |
| Background/rationale | 2 | Explain the scientific background and rationale for the investigation being reported | Lines 73-138 | Controversial findings of previous timeliness of care – overall survival studies in lung cancer, including the description of various sources of potential methodology bias (Lines 73-107); OnkoNetwork and the H2020 SELFIE project (Lines 108-138). |
| Objectives | 3 | State specific objectives, including any prespecified hypotheses | Lines 139-142 | The purpose of this study was to evaluate the impact of OnkoNetwork implementation on overall survival of NSCLC patients, and to characterize key changes in the timeliness and completeness of NSCLC care upon OnkoNetwork implementation. |
| Methods | | | |  |
| Study design | 4 | Present key elements of study design early in the paper | Lines 145-152 | A retrospective observational cohort study involving patients with any new solid organ cancer was conducted after the study protocol went through an ethical review and approval process by the Hungarian National Ethics Committee. (TUKEB, Decision No. 12412-2/2017/EKU). |
| Setting | 5 | Describe the setting, locations, and relevant dates, including periods of recruitment, exposure, follow-up, and data collection | Lines 147-152; 162-163; 170-172. | In the present NSCLC subgroup analysis, the intervention cohort was defined as all adult patients in the catchment area of the Moritz Kaposi General Hospital with new cases of NSCLC between December 2015 – November 2016. A historic control cohort was defined as all adult patients with new cases of NSCLC between September 2014 – August 2015 in the same Hospital (before the implementation of OnkoNetwork in October-November 2015). … Study data was retrospectively extracted from the prospectively maintained electronic medical system of the Hospital. … The end of patient follow-up period was determined as the date of death or the last appearance of the patient in the medical system of the hospital. Patients lost to follow-up were censored at their last visit. |
| Participants | 6 | (*a*) *Cohort study*—Give the eligibility criteria, and the sources and methods of selection of participants. Describe methods of follow-up  *Case-control study*—Give the eligibility criteria, and the sources and methods of case ascertainment and control selection. Give the rationale for the choice of cases and controls  *Cross-sectional study*—Give the eligibility criteria, and the sources and methods of selection of participants | Lines 152-160; 170-172. | Enrollment into OnkoNetwork was not an inclusion criterion in the intervention cohort, to minimize the risk of selection bias. For both cohorts, only patients with previously undiagnosed, new NSCLC cases were enrolled, excluding patients with i) subsequently confirmed benign conditions; ii) unexpectedly short care delay (multidisciplinary tumor board meeting within 3 days and/or treatment initiation within 7 days after first cancer suspect code), since these patients probably arrived at the hospital with a suspected case of cancer that was not completely new or were very severe cases requiring immediate care; iii) terminal stage patients with <30 days survival; and iv) patients lost to hospital follow-up within 30 days. Lung cancer patients with missing histology or with a mesothelioma diagnosis were also excluded (Fig 1). … The end of patient follow-up period was determined as the date of death or the last appearance of the patient in the medical system of the hospital. Patients lost to follow-up were censored at their last visit. |
|  |  | (*b*) *Cohort study*—For matched studies, give matching criteria and number of exposed and unexposed  *Case-control study*—For matched studies, give matching criteria and the number of controls per case | - | Not applicable |
| Variables | 7 | Clearly define all outcomes, exposures, predictors, potential confounders, and effect modifiers. Give diagnostic criteria, if applicable | Lines 163-175. | Residence urbanization level (urban / rural) and socio-economic development level (0-100, the higher the more developed) were mapped from publicly available data by postal code regions. Symptom onset was recorded as the first day of the month where no specific day was reported. For asymptomatic cases, onset was the date of the first investigation that raised the suspect of lung cancer. Time to multidisciplinary tumor board recommendation and to treatment initiation were calculated from the first cancer suspect code in the medical system. Cancer staging was performed based on the 7th edition of the AJCC cancer staging manual. Overall survival was measured from the date of the first lung cancer suspect code in the hospital. The end of patient follow-up period was determined as the date of death or the last appearance of the patient in the medical system of the hospital. Patients lost to follow-up were censored at their last visit. Data on diagnostic investigations and treatment modalities were extracted both from structured data of the medical system and from free-text medical reports. The anonymized study data is available as supportive information (S1 Datafile). |
| Data sources/ measurement | 8* | For each variable of interest, give sources of data and details of methods of assessment (measurement). Describe comparability of assessment methods if there is more than one group | Lines 162-175 | Study data was retrospectively extracted from the prospectively maintained electronic medical system of the Hospital. Residence urbanization level (urban / rural) and socio-economic development level (0-100, the higher the more developed) were mapped from publicly available data by postal code regions. Symptom onset was recorded as the first day of the month where no specific day was reported. For asymptomatic cases, onset was the date of the first investigation that raised the suspect of lung cancer. Time to multidisciplinary tumor board recommendation and to treatment initiation were calculated from the first cancer suspect code in the medical system. Cancer staging was performed based on the 7th edition of the AJCC cancer staging manual. Overall survival was measured from the date of the first lung cancer suspect code in the hospital. The end of patient follow-up period was determined as the date of death or the last appearance of the patient in the medical system of the hospital. Patients lost to follow-up were censored at their last visit. Data on diagnostic investigations and treatment modalities were extracted both from structured data of the medical system and from free-text medical reports. The anonymized study data is available as supportive information (S1 Datafile). |
| Bias | 9 | Describe any efforts to address potential sources of bias | Lines 152-153; 177-182; 193-211;  Supplementary information S2 and S3 | Enrollment into OnkoNetwork was not an inclusion criterion in the intervention cohort, to minimize the risk of selection bias. … A propensity score (PS) of belonging to the intervention cohort was calculated for each patient via multivariate logistic regression with all baseline parameters included in Table 1 as predictive factors, except for prior scintigraphy which was conducted in very few patients. To estimate the average treatment effect on the treated (ATT), the inverse probability of treatment weight was set to 1 in the intervention cohort and calculated with the PS/(1-PS) formula in the control cohort, and the calculated weights were applied in all subsequent descriptive and regression analyses. … Measurable baseline characteristics, diagnostic process indicators, treatment modalities, and intermediate outcomes assessed at treatment initiation, as listed in Table 2, were tested as potential predictors of overall survival in univariate and multivariate weighted Cox regression models. In addition to the intervention indicator, the multivariate models included either all baseline or all post-baseline variables, and non-significant variables were not removed from the final models. In the next step, a counterfactual analysis was performed: individual survival status at 1, 2, and 3 years was predicted from the weighted Cox regression model using intervention as the only covariate, given that the comparability of the two study groups was ensured by using the relevant prognostic factors which were unbalanced to create the propensity score. Rubin’s B and R statistics showed good balance of these prognostic factors after weighting. For the counterfactual scenario, the survival probabilities were predicted in the intervention cohort by setting their cohort indicator parameter from “intervention” to “control” before calculating the predictions. Effect size was estimated as the mean individual difference in the predicted probabilities in the intervention versus the counterfactual cohorts. Uncertainty in the effect estimates was estimated via bootstrapping. For each outcome, 1000 bootstrapped samples were generated from the original patient sample with replacement (the same patient could be included multiple times) as if the study were repeated 1000 times. The effect estimates were calculated for these 1000 samples as they were for the original sample, and the distribution of effect size was characterized by its 95% confidence interval and by the proportion of bootstrapped samples with positive survival benefit.  S2 and S3 includes the relevant statistical codes and outputs in R and Stata, respectively. |
| Study size | 10 | Explain how the study size was arrived at | Lines 136-138. | Given that the largest subgroup of the SELFIE OnkoNetwork study consisted of lung cancer patients, the authors first conducted a detailed clinical evaluation in this patient subgroup. |

Continued on next page

| Quantitative variables | 11 | Explain how quantitative variables were handled in the analyses. If applicable, describe which groupings were chosen and why | Tables 1-2;  Table S4a | Continuous variables with non-normal distribution were characterised by median and quartiles, and analysed by their quartiles as categorical. Tables 1 and 2, Days from onset to first hospital cancer suspect code: Q1 (0-13 days), Q2 (14-36 days), Q3 (37-76 days), Q4 (77-778 days). Table S4a, Treatment delay from first code, in days: median (quartiles). |
| --- | --- | --- | --- | --- |
| Statistical methods | 12 | (*a*) Describe all statistical methods, including those used to control for confounding | Lines 176-211;  Table 2;  Supplementary information S2 and S3 | Propensity score weighting, lines 176-189; Descriptive analyses, lines 191-192; Univariate and multivariate weighted Cox regression models, lines 193-  197 and Table2; Counterfactual analysis, lines 197-206; Uncertainty in counterfactual effect estimates via bootstrapping, lines 206-211.  S2 and S3 includes the relevant statistical codes and outputs in R and Stata, respectively. |
|  |  | (*b*) Describe any methods used to examine subgroups and interactions | - | Not applicable |
|  |  | (*c*) Explain how missing data were addressed | Tables 1-2 | {Patients with missing data were not excluded but analysed as stand-alone categories of the relevant variables.} Table 1, Clinical onset – “unknown”; Tumor histology – “NSCLC other/NOS”; Days from onset to first hospital cancer suspect code (“unknown”); Table 2, Stage at treatment initiation – “unknown”; ECOG performance at treatment initiation – “unknown”. |
|  |  | (*d*) *Cohort study*—If applicable, explain how loss to follow-up was addressed  *Case-control study*—If applicable, explain how matching of cases and controls was addressed  *Cross-sectional study*—If applicable, describe analytical methods taking account of sampling strategy | Lines 154-159; 170-172. | excluding … iv) patients lost to hospital follow-up within 30 days; … The end of patient follow-up period was determined as the date of death or the last appearance of the patient in the medical system of the hospital. Patients lost to follow-up were censored at their last visit. |
|  |  | (*e*) Describe any sensitivity analyses | - | Not applicable |
| Results | | | | |
| Participants | 13* | (a) Report numbers of individuals at each stage of study—eg numbers potentially eligible, examined for eligibility, confirmed eligible, included in the study, completing follow-up, and analysed | Lines 222-223;  Figure 1 | Of the 661 patients with suspected lung cancer, 296 NSCLC patients were included in the analyses (123 and 173 patients in the intervention and control cohorts, respectively; Fig 1). |
|  |  | (b) Give reasons for non-participation at each stage | Figure 1 | Figure 1 |
|  |  | (c) Consider use of a flow diagram | Figure 1 | Figure 1 |
| Descriptive data | 14* | (a) Give characteristics of study participants (eg demographic, clinical, social) and information on exposures and potential confounders | Lines 223-231;  Table 1  Table S4a  Table S4b | Enrollment into OnkoNetwork was 0% and almost 100% in the control and the intervention cohort, respectively. The study cohorts showed similar distributions by age, sex, residence urbanization level and socioeconomic development, tumor histology, and pre-hospital delays, with similar proportions of symptom-free, accidentally identified cases at clinical onset before propensity score weighting. However, the rate of completed chest CT and bronchoscopy investigations at study baseline showed a statistically significant difference between the cohorts (Table 1). These observed between-cohort differences were eliminated by the applied propensity score-based weighting (Table 1), resulting in Rubin’s B and R values within the recommended ranges of <25 and [0.5; 2], respectively [62].  Table 1. Baseline characteristics of the study population.  Table S4a. Overview of diagnostic and treatment procedures, by study cohorts.  Table S4b. Intermediate outcomes at last assessment before treatment initiation, by study cohorts. |
|  |  | (b) Indicate number of participants with missing data for each variable of interest | All Tables | {for all study variables, missing data are reported in the corresponding tables} |
|  |  | (c) *Cohort study*—Summarise follow-up time (eg, average and total amount) | Lines 262-263 | The mean follow-up duration was 1.48 years and 1.65 years in the intervention and control cohorts … |
| Outcome data | 15* | *Cohort study*—Report numbers of outcome events or summary measures over time | Lines 262-263 | … in the intervention and in the historic control cohorts with 42 and 81 reported deaths, respectively. |
|  |  | *Case-control study—*Report numbers in each exposure category, or summary measures of exposure | *-* | *Not applicable* |
|  |  | *Cross-sectional study—*Report numbers of outcome events or summary measures | *-* | *Not applicable* |
| Main results | 16 | (*a*) Give unadjusted estimates and, if applicable, confounder-adjusted estimates and their precision (eg, 95% confidence interval). Make clear which confounders were adjusted for and why they were included | Lines 264-288;  Table 2;  Supplementary information S2 and S3 | In univariate analyses, mortality was significantly higher in patients with a higher age, showing symptoms at clinical onset (as opposed to asymptomatic patients with accidental findings that raised the suspect of tumor), in patients with disease stage III, IV or unknown, in patients with ECOG 1, 2, or 3-4 (reference: ECOG 0), and in cases with cytology confirmation before treatment initiation. On the other hand, mortality was significantly lower in patients belonging to the intervention cohort, who had a prior chest CT at baseline, completed a PET-CT before treatment initiation, underwent resection surgery, and received chemotherapy. In a multivariate analysis including all baseline patient characteristics, mortality was significantly lower in patients belonging to the intervention cohort. The multivariate hazard ratio for belonging to the intervention cohort (0.63, 95%CI 0.41 – 0.98, p=0.039) was practically identical to the hazard ratio found in the univariate analysis (0.64, 95%CI 0.43 – 0.95), indicating that the applied weighting achieved a good balance of cohorts regarding the baseline parameters. The question of whether the overall survival benefit could be explained by intermediate outcomes in the putative causal chain was further investigated in a subsequent multivariate weighted Cox regression model that was adjusted to post-baseline patient characteristics (e.g., modalities of investigations conducted before treatment initiation, stage and functional status at treatment initiation, and treatment modalities applied, including surgical resection, chemotherapy, and radiotherapy as broad categories). As expected, belonging to the intervention cohort was not significantly associated with survival benefit in this model (HR=0.81, p=0.323), indicating that the included post-baseline patient path characteristics and intermediate outcomes at least partly explained the observed survival benefits of OnkoNetwork. Mortality was significantly lower in patients undergoing resection surgery and in patients receiving chemotherapy, and significantly higher in patients with stage IV disease and ECOG 3-4 status at treatment initiation (Table 2). Conducting a PET-CT investigation before treatment initiation also tended to show some association with improved survival, without statistical significance (p=0.055). Descriptive patterns of the identified post-baseline predictors of overall mortality by study cohorts are depicted in Fig 3.  Table 2. Association between mortality and patient pathway characteristics (weighted dataset).  S2 and S3 includes the relevant statistical codes and outputs in R and Stata, respectively. |
|  |  | (*b*) Report category boundaries when continuous variables were categorized | Tables 1-2 | Days from onset to first hospital cancer suspect code: Q1 (0-13 days), Q2 (14-36 days), Q3 (37-76 days), Q4 (77-778 days). |
|  |  | (*c*) If relevant, consider translating estimates of relative risk into absolute risk for a meaningful time period | - | Not applicable |

Continued on next page

| Other analyses | 17 | Report other analyses done—eg analyses of subgroups and interactions, and sensitivity analyses | - | Not applicable |
| --- | --- | --- | --- | --- |
| Discussion | | | | |
| Key results | 18 | Summarise key results with reference to study objectives | Lines 343-360. | Accordingly, our key finding is that the implementation of OnkoNetwork was associated with improved overall survival of NSCLC patients in a multivariate Cox regression model adjusted for the baseline characteristics of study participants. The multivariate hazard ratio for belonging to the intervention cohort was 0.63 (95%CI 0.41 – 0.98). The point estimate 0.63 indicates a large decrease in overall mortality hazard (37% decrease) which is consistent with the presented Kaplan-Meier plots in Fig 2. To further investigate whether this improvement could be explained by intermediate outcomes in the putative causal chain, a subsequent multivariate weighted Cox regression model was adjusted to post-baseline patient characteristics. As expected, intervention effect was not statistically significant in the latter model, whereas mortality was significantly lower in patients undergoing resection surgery and in patients receiving chemotherapy, and significantly higher in patients with stage IV disease and/or ECOG 3-4 status at treatment initiation. The use of PET/CT before treatment initiation also tended to be associated with longer overall survival (HR 0.62, 95%CI 0.38-1.01, not significant). Accordingly, the beneficial effect of OnkoNetwork implementation on lung cancer overall survival could be – at least partly – explained by a complex interplay of multiple post-baseline factors including broader exploitation of PET-CT imaging, higher proportion of patients with resectable disease, and lower proportion of stage IV patients at treatment initiation. Indeed, corresponding beneficial trends after OnkoNetwork implementation could be observed in descriptive analyses (Figure 3, not significant). |
| Limitations | 19 | Discuss limitations of the study, taking into account sources of potential bias or imprecision. Discuss both direction and magnitude of any potential bias | Lines 380-415. | Our study has important limitations. First, for ethical reasons, the implementation of OnkoNetwork did not include a randomization step on patient enrollment and the adopted observational study design is subject to selection bias. Studies like these frequently face this limitation when evaluating integrated care programs, consequently, the SELFIE consortium adopted a sophisticated methodology to minimize the risk of selection bias with propensity score weighting and counterfactual analysis as core elements, which was the methodology our analysis carefully followed.  Further limitations of our study include the moderate study size, which did not allow for more refined analyses of patient paths. The relatively broad categories of the measured patient pathway variables allowed for the definition of a manageable number of pathway events, but could hide important differences in the fine details. For more refined analyses, the use of more graded patient path indicator variables would be warranted in larger study populations, e.g., to investigate the type of chemotherapy and the number of chemotherapy episodes – instead of a high-level binary parameter whether chemotherapy was received by the patient or not. Moreover, larger study populations would allow for a lower probability of Type 1 error in statistical tests (e.g., alpha = 0.001).  Furthermore, the care delay intervals measured in our study were based on the OnkoNetwork interval definitions, with an atypical clock start (first cancer suspect code appearing in the medical system of the study Hospital). Hence, these periods cannot easily be mapped to the more and more widely used international care delay terminology established e.g. by the Aarhus statement [65]. The time from first cancer suspect code to the multidisciplinary tumor board recommendation interval in our study mostly resembles the secondary care interval minus the treatment interval as defined in the Aarhus statement.  As an additional limitation, we emphasize that our findings are linked to the Hungarian healthcare context that surrounds the OnkoNetwork model. Patient delay, length of diagnostic and treatment waitlists, and adherence of real-world clinical practice to national or institutional guidelines can be very different across countries or regions. In principle, the more room for improvement that exists in a health system in timely and quality assured care, the larger the clinical advantages that may be expected upon patient navigation system implementation, assuming that the implementation is feasible and is not blocked by legal, cultural or economic barriers. The authors highlight the importance of qualitative and quantitative assessment of model transferability to other countries, instead of merely extrapolating the study findings to other patient populations across dissimilar healthcare settings.  Finally, our detailed analysis in lung cancer patients was limited to the evaluation of clinical outcomes. For evidence-based decision making on the implementation of new patient navigation programs, additional research is warranted on health economic aspects and on patient experience, besides confirmatory findings on improved clinical outcomes. |
| Interpretation | 20 | Give a cautious overall interpretation of results considering objectives, limitations, multiplicity of analyses, results from similar studies, and other relevant evidence | Lines 417-425. | Our study showed a large improvement in the overall survival of NSCLC patients associated with the implementation of the OnkoNetwork patient navigation model in the Moritz Kaposi General Hospital in Kaposvár, Hungary. The positive impact of OnkoNetwork could be explained by multiple, statistically not significant effects (broader use of PET-CT during diagnostic workup, more patients diagnosed in surgically resectable stage, and slightly less advanced cases with stage IV and ECOG 3-4 status at diagnosis). Patient navigation is a valuable tool to improve lung cancer outcomes by facilitating timely and complete cancer diagnostics. Contradictory evidence in literature may be explained by various sources of bias, including the wait time paradox and model adjustment to intermediate outcomes conferring the survival benefits. |
| Generalisability | 21 | Discuss the generalisability (external validity) of the study results | Lines 402-411. | As an additional limitation, we emphasize that our findings are linked to the Hungarian healthcare context that surrounds the OnkoNetwork model. Patient delay, length of diagnostic and treatment waitlists, and adherence of real-world clinical practice to national or institutional guidelines can be very different across countries or regions. In principle, the more room for improvement that exists in a health system in timely and quality assured care, the larger the clinical advantages that may be expected upon patient navigation system implementation, assuming that the implementation is feasible and is not blocked by legal, cultural or economic barriers. The authors highlight the importance of qualitative and quantitative assessment of model transferability to other countries, instead of merely extrapolating the study findings to other patient populations across dissimilar healthcare settings. |
| Other information | |  | | |
| Funding | 22 | Give the source of funding and the role of the funders for the present study and, if applicable, for the original study on which the present article is based | - | Provided directly via the submission system, as requested by the Journal. |

*Give information separately for cases and controls in case-control studies and, if applicable, for exposed and unexposed groups in cohort and cross-sectional studies.

**Note:** An Explanation and Elaboration article discusses each checklist item and gives methodological background and published examples of transparent reporting. The STROBE checklist is best used in conjunction with this article (freely available on the Web sites of PLoS Medicine at http://www.plosmedicine.org/, Annals of Internal Medicine at http://www.annals.org/, and Epidemiology at http://www.epidem.com/). Information on the STROBE Initiative is available at www.strobe-statement.org.
