# Supplementary material for: Improved survival of non-small cell lung cancer patients after introducing patient navigation: A retrospective cohort study with propensity score weighted historic control
Source: PLoS One. 2022 Oct 25;17(10):e0276719. doi: 10.1371/journal.pone.0276719 (PMC9595513; doi:10.1371/journal.pone.0276719)
Supplement: Attachment — Submitted filename: Response to Reviewers.docx [file pone.0276719.s006.docx]

**Rebuttal Letter**

We thank the Editor and the three Reviewers for their helpful comments on our manuscript. Below is our response to each point raised by the Academic Editor and by the Reviewers. We hope that the corresponding revisions and the provided justifications are satisfactory and that the manuscript will be now suited for publication in PLOS ONE.

Sincerely, on behalf of all co-authors,

Prof. Zoltán Vokó

**Academic Editor:**

**The manuscript has been reviewed by three reviewers. Their overall comments have been positive. However, some issues and concerns have been underlined for improving the manuscript. The comments from reviewers are appended below the message for addressal. Additionally, elaborate on statistical (counterfactual) analysis, and why alpha = 0.05 for HR and PETCT analysis would be sufficient for decision making, given the potentially severe consequences of such a decision in the current case.**

*Response: Thank you very much for organizing the peer review process and for your comments. Although we do not see prominent negative consequences of cancer patient navigation in general, we agree with your caveats. The presented evidence is obviously not sufficient and complete for evidence-based decision making on the implementation of new patient navigation programs for non-small cell lung cancer patients. To support evidence-based decision making,* *additional research is warranted on health economic aspects and on the patient experience domains, besides confirmatory findings on clinical outcomes. Furthermore, regional differences in characteristics and performance of the hosting health systems also need to be duly considered.*

*Importantly, our manuscript did not claim that a complete evidence package is delivered for decision making on the implementation of new patient navigation programs. In contrast, our aim was to report on the improved clinical outcomes in association with the implementation of patient navigation in a single investigated program. The related study limitations were already declared in the Discussion section, and to better reflect the above considerations, the wording of these limitations has been adjusted as shown below:*

*“As an additional limitation, we emphasize that our findings are linked to the Hungarian healthcare context that surrounds the OnkoNetwork model. Patient delay, length of diagnostic and treatment waitlists, and adherence of real-world clinical practice to national or institutional guidelines can be very different across countries or regions. In principle, the more room for improvement that exists in a health system in timely and quality assured care, the larger the clinical advantages that may be expected upon patient ~~pathway management~~navigation system implementation, assuming that the implementation is feasible and is not blocked by legal, cultural or economic barriers. The authors highlight the importance of qualitative and quantitative assessment of model transferability to other countries, instead of merely extrapolating the study findings to other patient populations across dissimilar healthcare settings.*

*Finally, our detailed analysis in lung cancer patients was limited to the evaluation of clinical outcomes. For evidence-based decision making on the implementation of new patient navigation programs, additional research is warranted on health economic aspects and on patient experience, besides confirmatory findings on improved clinical outcomes.*

*~~Further research is warranted on the health economic implications of patient pathway monitoring programmes, subject to data availability on resource utilisation and healthcare costs at the healthcare system level, beyond the geographical scope of our hospital-based data collection in this study.”~~*

*We argue that the selected significance level of alpha = 0.05 is adequate for the study purpose, i.e., for exploratory hypothesis testing. We acknowledge that in confirmatory studies, a higher level of significance level could be warranted or multiple positive studies with alpha = 0.05 could be requested, subject to the specific decision context. To discuss the issue of the selected alpha level in our analyses, a corresponding statement is integrated into the Discussion section, as detailed at the responses to Reviewer 3.*

*Results of the counterfactual analyses have been elaborated, adding a short descriptive summary on the results:*

*“In other words, assuming that our study was repeated 1000 times on the population of the intervention cohort against the same hypothetical control subjects without the intervention, 977 studies would report a survival benefit in the intervention cohort at one year of follow-up. The average 1-year survival in the 1000 studies would be 71.6% in the intervention cohorts as compared to 59.3% in the control cohorts (difference 12.3%, 95%CI 0.2% - 24.9%).”*

*Thanks again for your constructive support in the peer review period, and please find below our detailed responses to the specific points.*

**1. Journal Requirements: When submitting your revision, we need you to address these additional requirements.. Please ensure that your manuscript meets PLOS ONE's style requirements, including those for file naming. The PLOS ONE style templates can be found at**

**https://journals.plos.org/plosone/s/file?id=wjVg/PLOSOne_formatting_sample_main_body.pdf and** [**https://journals.plos.org/plosone/s/file?id=ba62/PLOSOne_formatting_sample_title_authors_affiliations.pdf**](https://journals.plos.org/plosone/s/file?id=ba62/PLOSOne_formatting_sample_title_authors_affiliations.pdf).

*Response: Thank you, the manuscript has been checked and adjusted to the provided style requirements.*

**2. In your Methods section, please ensure that all variables included in the analyses have been adequately defined.**

*Response: Thank you for pointing out that some variables were not fully defined. The Methods section has been supplemented with descriptions for urban/rural status and socio-economic development of patient residence, together with the determination of onset date in asymptomatic cases, and with the applied cancer staging approach (AJCC TNM7). In addition, additional explanation of variables and their values is integrated into S1_datafile in the form of comments to column headings.*

**3. Thank you for stating the following in the Competing Interests section: "I have read the journal's policy and the authors of this manuscript have the following competing interests: the described research was conducted as part of the H2020 SELFIE project that has received funding from the European Union’s Horizon 2020 research and innovation programme under grant agreement No 634288. JGP, MCs, MRM, KI, ZK, and ZV are employees of SELFIE beneficiaries. The employer of JGP, MCs, ZK, and ZV received additional EU research grants related to the evaluation of smoking cessation interventions and national cancer screening programs. MM, ÉSE, GL, and ASz are employees of Móritz Kaposi General Hospital, this Hospital initiated the OnkoNetwork program and received study funding from the SELFIE grant." Please confirm that this does not alter your adherence to all PLOS ONE policies on sharing data and materials, by including the following statement: "This does not alter our adherence to PLOS ONE policies on sharing data and materials.” (as detailed online in our guide for authors http://journals.plos.org/plosone/s/competing-interests). If there are restrictions on sharing of data and/or materials, please state these. Please note that we cannot proceed with consideration of your article until this information has been declared.**

**Please include your updated Competing Interests statement in your cover letter; we will change the online submission form on your behalf.**

*Response: Thank you, we confirm that our potential conflicting interests declared in the above statement do not alter our adherence to PLOS ONE policies on sharing data and materials. Our adherence to data sharing policies is also clear from the provision of the study dataset as supplementary data file attached to the manuscript. It has already submitted as part of the original submission and is also part of the revised submission package. Accordingly, we fully agree with the requested additional statement and include it in our cover letter.*

**4. Please note that in order to use the direct billing option the corresponding author must be affiliated with the chosen institute. Please either amend your manuscript to change the affiliation or corresponding author, or email us at plosone@plos.org with a request to remove this option.**

*Response: Thank you for letting us know about this issue. We confirm that Semmelweis University is the primary affiliation of the corresponding author of this manuscript, and also of the co-author Prof. Zoltan Kalo. We adjusted the indicated affiliations on the title page accordingly (changing the sequence of the two affiliations for the corresponding author, Prof. Zoltan Voko and also for prof. Zoltan Kalo).*

**5. We note that you have included the phrase “data not shown” in your manuscript. Unfortunately, this does not meet our data sharing requirements. PLOS does not permit references to inaccessible data. We require that authors provide all relevant data within the paper, Supporting Information files, or in an acceptable, public repository. Please add a citation to support this phrase or upload the data that corresponds with these findings to a stable repository (such as Figshare or Dryad) and provide and URLs, DOIs, or accession numbers that may be used to access these data. Or, if the data are not a core part of the research being presented in your study, we ask that you remove the phrase that refers to these data.**

*Response: Thank you pointing out this incongruity. We confirm that all relevant data necessary for the reproduction of the presented analyses are made available for the readers as Supplementary Information (S1_Datafile). The “data not shown” phrase was referring to a single comparative sentence in the Discussion section, stating that unlike in the presented study population, the 44-day treatment initiation threshold could be safely met in patients with other solid organ cancers. Given that this comparison is not a key issue in the presented analyses of non-small cell lung cancer patients, both the comparative statement and the “data not shown” phrase have been removed from the manuscript.*

**6. While revising your submission, please upload your figure files to the Preflight Analysis and Conversion Engine (PACE) digital diagnostic tool, https://pacev2.apexcovantage.com/. PACE helps ensure that figures meet PLOS requirements. To use PACE, you must first register as a user. Registration is free. Then, login and navigate to the UPLOAD tab, where you will find detailed instructions on how to use the tool. If you encounter any issues or have any questions when using PACE, please email PLOS at figures@plos.org. Please note that Supporting Information files do not need this step.**

*Response: Thank you very much for this suggestion, PACE is really a great tool. The figures have been uploaded and automatically converted by PACE into the requested format (resolution changed to 600 PPI; dimensions adjusted to 7.5in W x 4.22in H; TIF files converted to valid TIF files; and renamed to Fig1, Fig2, Fig3). The converted figure files are included as stand-alone TIF files in the revision submission package.*

**Reviewer #1:**

**The authors use a retrospective cohort study to evaluate the implementation of the OnkoNetwork on overall survival for patients with non-small cell lung cancer. This is a single institution study conducted at Moritz Kaposi General Hospital. The authors should be commended for rigorously evaluating a care delivery implementation that has the potential to improve care for oncology patients. I have the following recommendations for strengthening this manuscript.**

*Response: Thank you very much for your review and comments! Please find our responses to the recommendations below.*

**1) The introduction should define in specific terms the "patient pathway management." After reading the manuscript, I was uncertain about how the authors define patient pathway management. For example, the American Society of Clinical Oncology defines a pathway as "detailed, evidence-based treatment protocols for delivering cancer care to patients with specific disease types and stages." Are the authors referring to the same type of treatment pathways? The main thrust of the introduction is around timeliness of care and biases. I think this could be shortened and further detail could be added around clinical pathways and how this study seeks to add to the pathways literature in oncology. The authors could cite various studies that have evaluated pathways in oncology and lung cancer such as DM Jackman, Journal of Oncology Practice, 2017 along with Neubauer MA, Journal of Oncology Practice, 2010.**

*Response: Thank you for identifying this conceptual opaqueness in our paper. Understanding that patient pathway management is not exactly the right term to describe the activities conducted in the OnkoNetwork program, we revised the manuscript as follows:*

- *Manuscript title has been adjusted: “Improved survival of non-small cell lung cancer patients after introducing patient ~~pathway management~~navigation: a retrospective cohort study with propensity score weighted historic control”; similarly, the short title has also been adjusted, replacing patient pathway management by patient navigation.*
- *The introduction section has been extended with the explanation of the term “patient navigation” and its relation to “patient pathway management”, referring to the proposed key papers and also to a published overview of the principles of patient navigation programs from the US perspective. Some European references are also provided, to better explain the broader context of the OnkoNetwork model. Efforts were made to shorten other sections of the Introduction to save space.*
- *Occurrences of the term “pathway management” throughout the manuscript were carefully checked and adjusted to more appropriate terms, for better conceptual clarity.*

**2) The components of the OnkoNetwork are not well described. I was uncertain of what the OnkoNetwork intervention was in practice. I think the manuscript would benefit from revising lines 85 - 88 to more specifically list the components of the intervention. How is the Onkonetwork different from standard of care. Perhaps the authors could include a diagram or figure as well.**

*Response: Thank you for this suggestion, the description of OnkoNetwork has been supplemented with the missing details, as follows:*

*“OnkoNetwork is an integrated care model offering ~~personalized~~ patient ~~pathway management~~navigation in inpatient and secondary outpatient care to adults with a new suspected solid organ cancer in the catchment area of the Moritz Kaposi General Hospital in Somogy county, Hungary. The planning, implementation and operation of OnkoNetwork have been described elsewhere [refs]. In a nutshell, the principles of patient navigation [1] were operationalized in OnkoNetwork via the 1) development and approval of cancer-specific investigational protocols; 2) education and technical training for physician and non-physician contributors; 3) monitoring and management of individual patient paths from initial cancer suspect through complete diagnostics to treatment decision by a multidisciplinary team (target: Tumour Board decision within 30 days), and then to treatment initiation (target: within 14 days after Tumour Board decision); 4) supportive IT platform integrated into the medical system of the hospital; and 5) nurse navigators and supervisory physicians with distinguished navigating responsibilities.* *Patient navigation starts at first cancer code in the medical system, without stop criteria – however, patient path monitoring is most intensive until treatment initiation [refs]. Importantly, treating physicians are free to defer from investigation protocols, subject to recorded justification in any case. ~~The primary aim of OnkoNetwork is to improve clinical outcomes in cancer patients via timely access to defragmented and quality assured diagnostics and treatment. In the OnkoNetwork model, personalized justification is requested for all patients without completed per protocol investigations plus multidisciplinary tumour board meeting within 30 days, and/or without treatment initiation within 44 days after first within-hospital cancer suspect ICD-10 code.”~~*

**3) In lines 115 - 177, the authors write the intervention cohort was defined as patients with a "new solid tumor" diagnosis. However, wasn't it actually patients with a new non-small cell lung cancer diagnosis? I would revise to make more clear.**

*Response: Thank you for raising this apparent discrepancy. The divergency of the methods description came from the fact that the source study was targeting all patients with solid organ cancers – whereas the subgroup analysis presented in this manuscript is restricted to patients with new non-small cell lung cancer. To facilitate the reading, the methods section has been adjusted with a consistent focus on NSCLC patients, making it clear at the beginning that this subgroup analysis is based on a study of all solid-organ cancers:*

*“A retrospective observational cohort study involving patients with any new solid organ cancer was conducted after ethical review and approval of the study protocol~~The quantitative evaluation of OnkoNetwork in the SELFIE project involved a retrospective study of clinical outcomes and timeliness of care parameters before and after OnkoNetwork implementation, with study protocol reviewed and approved by the Hungarian National Ethics Committee~~ (TUKEB, Decision No. 12412-2/2017/EKU) ~~before study commence~~. In the present NSCLC subgroup analysis ~~study~~, the intervention cohort was defined as all adult patients in the catchment area of the Moritz Kaposi General Hospital with a ~~suspected~~ new ~~solid tumour in the period of~~NSCLC between December 2015 – November 2016. A historic control cohort was defined as all adult patients with ~~suspected~~ new ~~solid tumours~~NSCLC between~~in the period of~~ September 2014 – August 2015 in the same Hospital (before the implementation of OnkoNetwork in October-November 2015). ~~Note that OnkoNetwork was launched in October-November 2015., without coincident introduction of other main reforms in Hungary.~~ …”*

**4) The data for the 2 cohorts are from 2014 - 2015 and 2015 - 2016. It is now 5-years old. The authors should provide some detail on why they are using data from several years ago to conduct their analysis and how this could impact their findings.**

*Response: The reason behind selecting the indicated time ranges for cohort enrolment criteria was adjusted to the timing of OnkoNetwork implementation: the two cohort enrolment periods covered 12-month ranges before and after OnkoNetwork implementation. Keeping the enrolment periods narrow (i.e., not continuing patient enrolment in the intervention cohort after November 2016, and not enrolling control subjects from previous years) made the study less prone to potential calendar effects. The few years elapsed from November 2016 allowed a reasonably long follow-up of overall survival in the enrolled patient cohorts, which is a strength of this study. To complete the picture, we regret to say that this manuscript was submitted to PLOS ONE in mid-January 2021 for peer review, and the last 9 months also added to the time lag between patient enrolment and study publication.*

**5) In the results section (line 190 - 191), the authors write that enrollment into the OnkoNetwork was 0% in the control and almost 100% in the intervention. I had assumed that to be expected given the design of the study. I was confused why the intervention group wasn't 100% enrolled in OnkoNetwork. Furthermore, do the authors need this statement given the methodology of the study design.**

*Response: Thank you for your comment on this important distinction. The reason behind the described design was to compare full annual cohorts, in order to minimize selection bias. Without this approach, one could assume that the intervention cohort was enriched in patients with better prognosis, e.g., by not inviting terminal-phase patients into the OnkoNetwork model. As a means to control for this type of selection bias, we intentionally compared full 12-month patient cohorts of the Hospital, without any inclusion criteria on OnkoNetwork enrollment in the intervention cohort. (Note also that propensity score based weighting and counterfactual analysis were applied to further minimize the risk of selection bias). Hence, we argue that this distinction is important, and added a corresponding sentence to the Methods section to better explain our approach, as follows: “Enrolment into OnkoNetwork was not an inclusion criterion in the intervention cohort, to minimize the risk of selection bias.”*

**6) The description of the overall survival in the results could benefit from further clarification. Specifically, did the implementation of the Onkonetwork lead to the improvement in survival or did the intervention group include a greater proportion of earlier stage patients and that is what led to the improvement in survival. I would ask the authors to review lines 248 - 249 where this is discussed and add further clarity. This is also discussed in the conclusion, lines 313 - 315.**

*Response: Thank you for highlighting this question regarding the interpretation of study results. The Results section includes the corresponding Hazard Ratio estimates with 95% confidence intervals for all univariate and multivariate analyses in Table2. Between-cohort differences in post-baseline parameters are described in the Descriptive analyses section and tabulated in Tables S4A and S4B. Description of the multivariate Cox regression analysis results adjusted to post-baseline factors is modified as follows:*

*“The question of whether the overall survival benefit could be explained by intermediate outcomes in the putative causal chain was further investigated in a subsequent multivariate weighted Cox regression model that was adjusted to post-baseline patient characteristics (e.g., modalities of investigations conducted before treatment initiation, stage and functional status at treatment initiation, and treatment modalities applied, including surgical resection, chemotherapy, and radiotherapy as broad categories). As expected, ~~Consequently, baseline parameters were not included in the next multivariate model that investigated the impacts of post-baseline patient pathway characteristics and intermediate outcomes. In this model,~~belonging to the intervention cohort was not significantly associated with survival benefit (HR=0.81, p=0.323), indicating that the included post-baseline patient path characteristics and intermediate outcomes at least partly explained the observed survival benefits of OnkoNetwork. Mortality was significantly lower in patients undergoing resection surgery and in patients receiving chemotherapy, and significantly higher in patients with stage IV disease and ECOG 3-4 status at treatment initiation (Table 2). Conducting a PETCT investigation before treatment initiation also tended to show some association with improved survival, without statistical significance~~had a protective effect in this analysis, with borderline statistical significance~~ (p=0.055). Descriptive patterns of the identified post-baseline predictors of overall mortality by study cohorts are depicted in Fig 3.”*

*Our interpretation of the results is that the implementation of Onkonetwork was associated with an improvement in NSCLC overall survival. This observation was statistically significant in multivariate Cox regression analysis of the weighted samples with excellent baseline comparability, adjusted to all baseline patient factors.* *The question whether this improvement could be mediated by intermediate outcomes in the putative causal chain was further investigated in a subsequent multivariate weighted Cox regression model, adjusted to post-baseline patient characteristics. As expected, intervention effect was not statistically significant in the latter model, whereas mortality was significantly lower in patients undergoing resection surgery and in patients receiving chemotherapy, and significantly higher in patients with stage IV disease and/or ECOG 3-4 status at treatment initiation. The use of PET/CT before treatment initiation also tended to be associated with longer overall survival (HR 0.62, 95%CI 0.38-1.01, not significant). Accordingly, the beneficial effect of OnkoNetwork implementation on lung cancer overall survival could be – at least partly – explained by slightly higher resectability rate and slightly lower proportions of patients with stage IV disease and/or ECOG 3-4 status at treatment initiation in the intervention cohort. Indeed, corresponding beneficial trends after OnkoNetwork implementation could be observed in descriptive analyses (Figure 3) of our limited sample of 123 intervention and 173 control subjects.*

*We made strong efforts to exclude the role of selection bias when comparing the intervention and control cohorts: we compared full annual cohorts, and achieved very good baseline balance via propensity score-based weighting. Furthermore, counterfactual analysis was also conducted, where intervention cohort patients were compared to a hypothetical counterfactual cohort consisting of hypothetical patients with identical baseline characteristics, except for assuming that they did not receive the intervention. In this counterfactual analysis, overall survival was consistently higher in the intervention cohort, with survival benefits in more than 97% of the generated bootstrap samples.*

*Hence, we argue that our conclusions can be held, and propose the below adjustment to make the wording of the conclusions clearer:*

*“Our study showed a large improvement in the overall survival of NSCLC patients associated with the implementation of the OnkoNetwork patient navigation model in the Moritz Kaposi General Hospital in Kaposvár, Hungary. The positive impact of OnkoNetwork could be ~~mediated~~ explained by multiple, statistically not significant effects (broader use of PET-CT imaging ~~in the~~during diagnostic workup ~~phase~~, more patients diagnosed in ~~the~~ surgically resectable stage~~phase~~ ~~of the disease~~, and by slightly less advanced cases with stage IV and ECOG 3-4 status at diagnosis). Patient navigation is a valuable tool to improve lung cancer outcomes by facilitating timely and complete cancer diagnostics and treatment initiation. Contradictory evidence in literature may be explained by various sources of bias, including the wait time paradox and model adjustment to intermediate outcomes conferring the survival benefits.”*

**7) I think the conclusion would benefit from further discussion of how this manuscript adds to our knowledge of oncology clinical pathways and pathway management.**

*Response: Thank you for this suggestion. The conclusion has been adjusted to better reflect patient navigation, and we made a clear distinction from the concept of patient pathway management already in the Introduction section. Scientific literature on the clinical impacts of patient navigation programs in Central- and Eastern European countries is sparse, hence, reporting on the clinical impacts of OnkoNetwork implementation adds to our knowledge on real-world performance of patient navigation programs in a less developed EU Member State and in an important patient segment with high incidence and mortality – lung cancer. The generalizability of our findings has important limitations as detailed in the Discussion section (in brief: inherent limitation of non-randomized studies; moderate study population size and broad categories for patient path description; care delays terminology of the OnkoNetwork model not fully in line with the Aarhus statement; and the study context was embedded into the Hungarian healthcare system, requiring thorough qualitative and quantitative assessment of model transferability to other countries, instead of merely extrapolating the study findings to other patient populations across dissimilar healthcare settings). Besides the brief summary of specific findings of our study, a caveat against multivariate model adjustment to intermediate outcomes of patient navigation programs is also incorporated into the conclusion section.*

**Reviewer #2:**

**Overall the manuscript is well written and have addressed importance of health care management in patient outcome. However, there are few points need to be taken care of before accepting the manuscript for publication.**

*Response: Thank you very much for your review and comments! Please find our responses to the specific points below.*

**This is an observational study where the authors have compared the overall survival of patients with NSCLC either have treatment delay or not. The exclusion and inclusion criteria used for the study patients are clear; however, the recruitment of patients to control and intervention cohorts were non-randomised. This process could have introduced biased in the cohort selection. The authors have used propensity score to reduce bias, yet it is important to highlight the impact of randomisation in this study.**

*Response: We agree with the Reviewer that randomization of patients into the OnkoNetwork model would have allowed a more robust analysis. However, the OnkoNetwork model was implemented to cover all patients with suspected new solid organ cancer, without a randomization step; and randomization to longer delays and/or to less quality assurance in oncology care would be an ethically disputable approach. Hence, the observational study design has important advantages in this research area. Inherent limitations of the study design are discussed in the Discussion section, as follows:*

*“Our study has important limitations. First, for ethical reasons, the implementation of OnkoNetwork did not include a randomization step on patient enrolment and the adopted observational study design is subject to selection bias. Studies like these frequently face this limitation when evaluating integrated care programs, consequently, the SELFIE consortium adopted a sophisticated methodology to minimize the risk of selection bias with propensity score weighting and counterfactual analysis as core elements, which was the methodology our analysis carefully followed.”*

**Was the treatment regimen across the cohort uniform? If not, then it is important to discuss that how could different regiment impact on survival. In the intervention cohort, more patients underwent surgical resection, this could have contributed to the overall survival of the patients compared to those only have received chemotherapy. While comparing overall survival, uniformity in treatment regimen should be considered amongst the groups.**

*Response: Thank you for raising this important consideration. Indeed, our results also confirmed the findings of several prior studies that NSCLC overall survival is positively associated with resection surgery, and also with chemotherapy. When evaluating the clinical impacts of a patient navigation program for NSCLC patients, baseline comparability and balance of study cohorts with and without the navigation program should be guaranteed. On the other hand, a key principle of patient navigation programs is to achieve changes in the care process: timeliness and quality assurance of the diagnostic workup and treatment initiation. For lung cancer resectability, timely diagnosis is crucial. Hence, adjustment to resectability rate in a multivariate Cox regression analysis of overall survival of NSCLC patients would measure only part of the impact of the patient navigation program – cleaned from any beneficial effect on stage distribution at the end of the (sometimes frustratingly slow) diagnostic workup.*

*To handle this complexity, our research followed a two-step approach. First, we made strong efforts to achieve good baseline balance of cohorts and to minimize selection bias, by i) comparing full annual cohorts before and after OnkoNetwork implementation; ii) narrow enrollment periods (not enrolling control patients before September 2014 and intervention patients after November 2016) to control for gradual changes in lung cancer care practice / available newer treatment options over study years; iii) propensity score based weighting to achieve excellent baseline balance of cohorts; and iv) counterfactual analysis, investigating the intervention effectiveness versus a hypothetical population with perfectly matched baseline characteristics to the intervention cohort population. Second, we conducted two separate multivariate regression models: one adjusted to all baseline patient characteristics, and another one adjusted to post-baseline patient characteristics (potential intermediate outcomes conferring the longer overall survival). Interpretation of the two model results is different: the first model investigated the overall effect of OnkoNetwork on overall survival; while the second model investigated whether the observed benefit could be explained by the included post-baseline characteristics. Our conclusion is that OnkoNetwork implementation was associated with a large improvement in the overall survival of NSCLC patients. The positive impact of OnkoNetwork could be explained by multiple, statistically not significant effects on the broader use of PET-CT imaging during diagnostic workup, more patients diagnosed in surgically resectable stage, and slightly less advanced cases with stage IV and ECOG 3-4 status at diagnosis.*

**The quality (resolution) of the graphs need to be improved, it is difficult to read and interpret.**

*Response: Thank you for pointing this out. The figures have been quality-checked and converted to appropriate format using the PACE tool, in compliance with the guidance of the Academic Editor.*

**Reviewer #3:**

**Overall the paper is well presented although the language should be improved to increase readability.**

*Response: Thank you very much for your review and suggestions! To improve the language of our manuscript, a native speaker review was arranged. Please find our responses to the specific issues below.*

**Major:**

**Line 240: The p-value of the HR is given as p=0.039. What test has been used? I assume alpha = 0.05 which then it is significant. However, in a medical context a significance level of alpha = 0.001 should be used in which case it is not significant. Discuss!**

*Response: Thank you for your comment. For all Cox regression analysis results, the statistical significance of individual regression coefficients was estimated in Wald tests with alpha = 0.05. The null hypothesis was that the Hazard Ratio = 1 (i.e., there is no association between the investigated regressor and the model outcome). The selected significance level of alpha = 0.05 is considered to be adequate for the study purpose, i.e., for exploratory hypothesis testing. We acknowledge that in pivotal / confirmatory studies, a higher level of significance level could be warranted or multiple positive studies with alpha = 0.05 could be requested, subject to the specific decision context. However, given that both the p values and the point estimate confidence intervals are numerically reported, the Readers are sufficiently informed to make their custom interpretation on the statistical strength of our observations. To discuss the issue of the selected alpha level in our analyses, a corresponding statement is integrated into the Discussion section:*

*“Further limitations of our study include the moderate study size, not allowing for more refined analyses of patient paths. The relatively broad categories of the measured patient pathway variables allowed the definition of a manageable number of pathway events, but could hide important differences in the fine details. For more refined analyses, the use of more graded patient path indicator variables would be warranted in larger study populations, e.g., to investigate the type of chemotherapy and the number of chemotherapy episodes – instead of a high-level binary parameter whether chemotherapy was received by the patient or not. Moreover, larger study populations would allow for lower probability of Type 1 error in statistical tests (e.g., alpha = 0.001).”*

**Line 250: P-value = 0.055 for PETCT. This is even not significant for alpha = 0.05 and not at all significant for alpha = 0.001.**

*Response: We agree with the observation that the hazard rate for conducting a PETCT investigation before treatment initiation did not reach the level of statistical significance for alpha = 0.05. The original manuscript referred to this association as having borderline significance: “Conducting a PETCT investigation before treatment initiation also had a protective effect in this analysis, with borderline statistical significance (p=0.055).” Now this sentence has been rephrased as follows: “Conducting a PETCT investigation before treatment initiation also tended to show some association with improved survival, without statistical significance (HR 0.62, 95%CI 0.38-1.01, p=0.055).”*

**It seems the key results of the paper show no statistical significance. Discuss!**

*Response: The key result of our paper is that the implementation of OnkoNetwork, a complex patient navigation program was associated with improved overall survival of non-small cell lung cancer patients in a multivariate Cox regression model adjusted for the baseline characteristics of study participants. The multivariate hazard ratio for belonging to the intervention cohort was 0.63, with 95%CI of 0.41 – 0.98. The 95% confidence interval excluded 1, hence, statistical significance is claimed (at p < 0.05). The point estimate of 0.63 indicates a large decrease in overall mortality hazard (37% decrease) which is consistent with the presented Kaplan-Meier plots in Fig 2. This model was adjusted for age, sex, socioeconomic surrogates (residence type and micro-regional development status), lung cancer histology, clinical onset (accidental finding/symptomatic/unknown), patient delay before first hospital visit quartiles, and completed investigations before entering the Hospital with the first lung cancer code (prior chest CT; prior bronchoscopy; prior PETCT; prior brain imaging). This model was intentionally not adjusted for post-baseline events and characteristics which could be in the causal chain between the patient navigation intervention and overall survival.*

*In another multivariate model adjusted for post-baseline events and characteristics (completed investigations before treatment initiation; treatment modalities selected; cancer stage and patient ECOG status at treatment initiation), the multivariate hazard ratio for belonging to the intervention cohort was 0.81, with 95%CI of 0.54 – 1.23 (not significant). Notably, many of the included post-baseline events are recognized elements of the causal chain between patient navigation and improved overall survival. The key ambition of OnkoNetwork is to deliver more timely and complete diagnostics and multidisciplinary treatment to cancer patients, and the timelier and more complete the diagnostic workup is, the more patients are expected to be diagnosed in a surgically resectable stage and to receive their most appropriate treatment in general. Hence, the non-significant hazard rate of the group effect in the latter multivariate model shall not be interpreted as evidence against the effectiveness of the intervention, but rather as a finding that the beneficial effects of the intervention are at least partly mediated by some of the included post-baseline factors / intermediate outcomes (e.g., higher rate of patients who were still in resectable stage at the end of the diagnostic investigations), keeping in mind that the study cohorts were full annual patient samples (to avoid cherry-picking of less severe patients in the intervention arm) and were well balanced for all measured baseline parameters using propensity score based weights. The counterfactual analysis was an additional effort to control for selection bias – in this analysis, the impact of the intervention on overall survival was modelled on the very same patients, with exact match of baseline parameters between the intervention cohort and their hypothetical clones in the counterfactual cohort.*

*We strongly believe that our paper with the above distinctions on methodology and clinical interpretation will meaningfully contribute to the ongoing scientific discussion on the clinical impacts of patient navigation programs in lung cancer. To make the discussion clearer on the key results of our paper, the corresponding paragraph of the discussion section has been revised as follows:*

*“Accordingly, our key finding is that the implementation of OnkoNetwork was associated with improved overall survival of NSCLC patients in a multivariate Cox regression model adjusted for the baseline characteristics of study participants. The multivariate hazard ratio for belonging to the intervention cohort was 0.63 (95%CI 0.41 – 0.98). The point estimate 0.63 indicates a large decrease in overall mortality hazard (37% decrease) which is consistent with the presented Kaplan-Meier plots in Fig 2. To further investigate whether this improvement could be explained by intermediate outcomes in the putative causal chain, a subsequent multivariate weighted Cox regression model was adjusted to post-baseline patient characteristics. As expected, intervention effect was not statistically significant in the latter model, whereas mortality was significantly lower in patients undergoing resection surgery and in patients receiving chemotherapy, and significantly higher in patients with stage IV disease and/or ECOG 3-4 status at treatment initiation. The use of PET/CT before treatment initiation also tended to be associated with longer overall survival (HR 0.62, 95%CI 0.38-1.01, not significant). Accordingly, the beneficial effect of OnkoNetwork implementation on lung cancer overall survival could be – at least partly – explained by a complex interplay of multiple post-baseline factors including broader exploitation of PET-CT imaging, higher proportion of patients with resectable disease, and lower proportion of stage IV patients at treatment initiation. Indeed, corresponding beneficial trends after OnkoNetwork implementation could be observed in descriptive analyses (Figure 3, not significant).*

*~~To understand the mechanisms by which OnkoNetwork could contribute to the observed improvement in overall survival, post-baseline patterns in the diagnostic procedures, treatment modalities and in intermediate outcomes were also investigated. No statistically significant difference was found in these parameters across the study cohorts in descriptive analyses (Tables S4a and S4b). In the multivariate Cox regression analysis including all post-baseline parameters, stage IV disease and ECOG 3-4 status were associated with shorter survival, whereas performing resection surgery and receiving chemotherapy were statistically significant predictors of longer survival, and conducting a PET-CT scan before treatment initiation was also a protective factor with borderline significance (Table 2). Four of these factors showed non-significant improvement in the intervention cohort (Figure 3). Intervention was a statistically non-significant protective factor when the post-baseline parameters were included in the model, suggesting that the survival benefits associated with the implementation of OnkoNetwork could be mediated by a complex interplay of multiple post-baseline factors including broader exploitation of PET-CT imaging, higher proportion of patients with resectable disease, and lower proportion of stage IV patients at treatment initiation.~~”*

**Minor:**

**The language of the paper should be improved.**

*Response:* *Thank you for letting us know about this weakness - a native speaker review and editing was arranged to improve the language of our manuscript.*

**Section title 'Descriptive analysis'. In this section a survival analysis is discussed which is not a descriptive analysis. I suggest to change the section title.**

*Response: Thank you for your suggestion on the subsection title. Accordingly, the “Overall survival” subsection has been renamed to “Cox regression analysis of overall survival”, whereas the “Descriptive analyses” subsection has been renamed to “Descriptive and Kaplan-Meier analysis”.*

We thank again for all the constructive and helpful comments of the Editor and the Reviewers to increase the scientific merit of our manuscript, and strongly hope that the revised manuscript will meet the high standards of PLOS ONE for publication.
