# Supplementary material for: Improved survival of non-small cell lung cancer patients after introducing patient navigation: A retrospective cohort study with propensity score weighted historic control
Source: PLoS One. 2022 Oct 25;17(10):e0276719. doi: 10.1371/journal.pone.0276719 (PMC9595513; doi:10.1371/journal.pone.0276719)
Supplement: Attachment — Submitted filename: Response to Reviewers.docx [file pone.0276719.s007.docx]

**Rebuttal Letter**

We thank the Editor and the Reviewers for their helpful comments on our manuscript. Below is our response to each additional point raised by the Academic Editor and Reviewer 3. We hope that the corresponding revisions and the provided justifications are satisfactory and that the manuscript will be now suited for publication in PLOS ONE.

Sincerely, on behalf of all co-authors,

Prof. Zoltán Vokó

**Academic Editor:**

**The study finding would be an excellent addition to the existing knowledge base. However, appropriate statistical treatment of the data remains desirable. For this reason alone, I am inclined to place it as a major revision.**

*Response: Thank you very much for organizing the peer review process and for your helpful comments to further improve our manuscript. We carefully considered all received comments and submit the revised version attached.*

**The authors would like to suitably address the concerns of Reviewer number 3.**

*Response: Thank you for highlighting the corresponding weaknesses. Please, see our responses to Reviewer 3 below. We hope that the corresponding adjustments will be satisfactory and our study will be accepted for publication.*

**Additionally, the statistical methods/methodology be sufficiently elaborated, and the discussion enriched accordingly to make the point.**

*Response: Thank you, we agree that the criticism that the statistical methodology need to be better elaborated. For this reason, the statistical methods description has been supplemented with the description of propensity score-based weight calculation method, with the description of Kaplan-Meier analysis and the applied statistical test, and with the explicit identification of study populations for each analysis. In addition, Table 2 and Rubin’s B and R findings have been moved to the Results section, and the rationale for the second multivariate model has been clarified.*

*“To estimate the average treatment effect on the treated (ATT), the inverse probability of treatment weight was set to 1 in the intervention cohort and calculated with the PS/(1-PS) formula in the control cohort, and the calculated weights were applied in all subsequent descriptive and regression analyses. This propensity score weight calculation method is also known as “standardized mortality ratio weighting” {Desai & Franklin, BMJ, 2019}. It is a well-established method of estimating treatment effect in observational studies to ensure comparability of the treatment groups as much as possible based on observed potential prognostic covariates which might have been associated with treatment selection. Baseline balance before and after weighting was assessed by Rubin’s B and R statistics as explained in the Discussion section.” … “Kaplan-Meier analysis of overall survival was conducted both in the unweighted and weighted study population, with log-rank test of statistical significance.” … “Measurable baseline characteristics, diagnostic process indicators, treatment modalities, and intermediate outcomes assessed at treatment initiation~~, as listed in Table 2,~~ were tested as potential predictors of overall survival in univariate and multivariate weighted Cox regression models in the weighted sample. In addition to the intervention indicator, the multivariate models included either all baseline or all post-baseline variables, in the latter model to test whether the association of intervention with overall survival was explained by improvements in intermediate outcomes.”*

*Furthermore, the Discussion section has been enriched with three short paragraphs discussing the available options and justifying the selected methods for propensity score adjustment; interpreting Rubin’s B and R parameters with explanation and reference; and better explaining the counterfactual analysis, respectively. To avoid redundance in this rebuttal letter, corresponding changes in the Discussion section are detailed below, at our responses to Reviewer 3.*

**The authors would like to be conservative while drawing the conclusion from their data.**

*Response: Indeed, our conclusion has been reshaped, putting less emphasis on the observed overall survival benefit in the specific program evaluated, since our observations were based on a moderate study population size and the statistical significance of improved overall survival was modest – although still significant at p < 0.05 in the multivariate analysis. Instead, a more generalizable conclusion of our study is derived, as shown below:*

*Conclusions*

*Our study showed a ~~large~~ promising improvement in the overall survival of NSCLC patients associated with the implementation of the OnkoNetwork patient navigation model in the Moritz Kaposi General Hospital in Kaposvár, Hungary. The positive impact of OnkoNetwork could be explained by multiple, statistically not significant ~~effects~~ beneficial trends (broader use of PET-CT during diagnostic workup, more patients diagnosed in surgically resectable stage, and slightly less advanced cases with stage IV and ECOG 3-4 status at diagnosis). Patient navigation is a valuable tool to improve lung cancer outcomes by facilitating timely and complete cancer diagnostics. ~~Contradictory evidence in literature may be explained by various sources of bias, including the wait time paradox and model adjustment to intermediate outcomes conferring the survival benefits.~~An important generalizable conclusion of our study is that the selection of regressors in multivariate survival analysis should be conscious and the model results should be interpreted in accordance with the selected model structure. When including putative effect mediators as explanatory variables in the analysis of association between patient navigation and overall survival, only the remaining association independent from the included explanatory variables will be estimated. For the overall assessment of the association, regression analysis shall not be adjusted to intermediate outcomes or putative effect mediators.*

**Please note that funding information should not appear in the Acknowledgments section or other areas of your manuscript. We will only publish funding information present in the Funding Statement section of the online submission form. Please remove any funding-related text from the manuscript.**

*Response: Funding information has been removed from the manuscript as requested, except for the Funding Statement.*

**Please upload a new copy of Figure 2 as the detail is not clear. Please follow the link for more information: https://blogs.plos.org/plos/2019/06/looking-good-tips-for-creating-your-plos-figures-graphics/**

*Response: Figure 2 has been redrawn at higher resolution, checked and adjusted using PACE and uploaded together with the revised version.*

**In your Methods section, please ensure that all variables included in the analyses have been adequately defined.**

*Response: We regret to acknowledge that despite our previous intention and efforts, some variables were still not properly defined in the Methods section. Now we carefully checked the manuscript, standardized the names of variables where it was not fully consistent across the various chapters, and provided detailed definitions for all study variables in the Methods section, as shown below:*

*“Data collection*

*Study data was retrospectively extracted from the prospectively maintained electronic medical system of the Hospital. Residence urbanization level (urban / rural) and socio-economic development score (0-100, the higher the more developed) were mapped from publicly available data by postal code regions. ~~Symptom~~ Clinical onset was recorded as the* *earliest recorded date of clinical symptoms of subsequently diagnosed lung cancer, and as the first day of the month where* *only the month was recorded without data on the ~~no~~ specific day ~~was reported~~. For asymptomatic cases, clinical onset was the date of the first investigation that raised the suspect of lung cancer. Time to multidisciplinary tumor board recommendation, and time to treatment initiation (treatment delay) were calculated from the first cancer suspect code in the medical system as day 0. Cancer staging including TNM was performed based on the 7th edition of the AJCC cancer staging manual. Overall survival was measured from the date of the first lung cancer suspect code in the hospital as day 0. The end of patient follow-up period was determined as the date of death or the last appearance of the patient in the medical system of the hospital. Patients lost to follow-up were censored at their last visit. Data on the completed diagnostic investigations (bronchoscopy, chest CT, PET-CT, scintigraphy, cytology confirmation, brain imaging (MR or CT) and initiated active anticancer treatment modalities (chemotherapy, radiotherapy, and surgery) were extracted both from structured data of the medical system and from free-text medical reports.* *Resection surgery included typical and atypical segment resection, lobectomy, and pneumonectomy,* *whereas metastasis resection and explorative surgery were not classified as resection surgery. Investigations completed before first suspected cancer code at the hospital (i.e., before study baseline) are referred to as “prior investigations” (e.g., prior chest CT) in manuscript text and tables. Variables on completed investigations until treatment initiation included either prior investigations or post-baseline investigations that were completed before treatment initiation. Presence or absence of investigations were evaluated as binary factors. Functional performance of patients was assessed based on routinely collected data before cancer treatment initiation using the Eastern Cooperative Oncology Group (ECOG) Performance Status scale.* *Largest tumor diameter measured in millimeters was extracted from pre-treatment medical records. Enrolment into OnkoNetwork was assessed based on the signed informed consent forms; enrolment was not possible for the historic control and was optional for the intervention cohort. The anonymized study data is available as supportive information (S1 Datafile).“*

*In addition, variable names have been adjusted in Table 1, and the term “functional status” was replaced by “ECOG status” throughout the text.*

*We are grateful for the careful editorial review and comments.*

**Reviewers #1 and #2:**

**All comments have been addressed. No further reviewer comments to the authors.**

*Response: Thank you very much for your review and previous comments that helped a lot to improve our manuscript.*

**Reviewer #3:**

**The following review relates to the statistical analysis. Overall, the paper uses advanced methodology to address an interesting problem. The main problem of the paper is that the methodology is not motivated nor alternative approaches are discussed. Furthermore, the statistical methods are not presented in a way as needed for such an article.**

*Response: Thank you very much for your review and comments to further improve our manuscript. Please find our detailed responses to your specific comments below.*

**Propensity score: A discussion of the propensity score needs to be presented explaining why it is needed and why there are no alternatives. Add citations to methodological papers discussing this in detail.**

*Response: Thank you very much for pointing out this weakness of our manuscript. The Discussion section has been extended with the corresponding details, as shown below:*

*“Propensity scores are frequently applied for confounding adjustment in observational studies via matching, stratification, adjustment as a regressor, or weighting, especially in studies estimating treatment effect. An important limitation of the propensity score matching approach is that unmatched observations, even if falling within the matching caliper, are discarded from the analysis {Desai & Franklin, BMJ, 2019}. Weighting based on propensity scores has the advantages of keeping most observations in the analysis, and unlike the inclusion of propensity scores in the regression model, it allows transparent reporting of baseline balance before and after adjustment {Desai & Franklin, BMJ, 2019}. Our propensity score weighting approach (“standardized mortality ratio weighting”) was selected for the target inference (average treatment effect on the treated, ATT) as harmonized across all model evaluations of the SELFIE consortium, and in compliance with current literature recommendations {Desai & Franklin, BMJ, 2019} and practice {Schröder et al, 2021; Sarayani et al, 2021}. It is a limitation of our study that no sensitivity analyses were performed using alternative propensity score adjustment methods, but the method we followed is considered to be the best performing method among propensity score adjustment and importantly, the ~~The inverse probability treatment~~adopted weighting ~~using the propensity score approach~~method eliminated the statistically significant baseline differences in pre-study chest CT and bronchoscopy rates.”*

**Counterfactual analysis: The same needs to be provided for the counterfactual information.**

*Response: Thank you very much for this additional point. The Discussion section has been extended with the corresponding details, as shown below:*

*Counterfactual impact evaluation is a valuable tool for comparing the outcomes of interest in subjects exposed to a program to outcomes that would have been achieved if the intervention had not been implemented {Joint Research Centre of the European Commission, https://ec.europa.eu/jrc/en/research-topic/counterfactual-impact-evaluation}. In studies without randomization, the counterfactual analysis is frequently focusing on simulated outcomes that could have occurred under different conditions, e.g., without exposure to the investigated program {Trends Psychiatry Psychother. 2020. 42(4):318-328; Nature Medicine, 2021, 27:239-243.}. In our study, ~~The~~the estimated hazard rate of intervention was applied in the counterfactual analysis where all baseline characteristics of the study patients showed identical patterns in the two compared cohorts, except for the assumption that counterfactual cohort patients did not receive the intervention. The counterfactual analysis results (Table 3) ~~indicated strong evidence for~~was in good agreement with the observed survival benefit associated with the introduction of OnkoNetwork, with point estimates ~~that were~~ above 10 percent point improvement at year 1, and above 15 percent point improvements in ~~the~~ 2-year and 3-year overall survival. Furthermore, the applied bootstrapping exercise, i.e., generation of multiple hypothetical study populations via sampling from the program cohort with replacement allowed the investigation of uncertainty in the counterfactual analysis point estimates. The results presented in Table 3 indicate that the probability of positive effect on survival was above 97% at all three timepoints. This finding means that if the same study was repeated 1000 times in similar populations, more than 970 of these hypothetical studies would report at least some survival benefit upon OnkoNetwork implementation. However, this analysis does not inform us about the expected statistical significance of survival benefits in those hypothetical studies.*

**Rubin's B and R statistics: Same as above.**

*Response: Thank you for your comment, the revised manuscript has been extended with the below short paragraph on Rubin’s B and R parameters with explanation and reference to key methodology papers.*

~~It also resulted in~~ Rubin’s B and R values ~~that i~~ndicate~~d a high baseline similarity between the intervention and control cohorts after weighting (Table 1).~~ the standardized mean difference and the variance ratio of propensity scores between study cohorts, respectively. Rubin suggested that B <25 and R between 0.5 and 2 indicate a sufficient overall balance {Rubin, 2001, Health Services & Outcomes Research Methodology 2:169–188.}. In our study, baseline balance was not in the acceptable range before weighting (Rubin’s B = 66.526), but a good baseline balance was achieved after propensity score weighting (Rubin’s B = 9.226, Rubin’s R = 1.294, and no significant difference in any of the observed baseline covariates; Table 1). Hence, the confounding effect of baseline covariates was eliminated in the weighted dataset and the effect estimates can approximate the causal effect {Rosenbaum P.R., Rubin D.B., Biometrika. 1983; 70: 41-55; }. Elimination of baseline imbalance in the weighted dataset was also confirmed by the lack of effect of baseline parameters on the mortality estimate of intervention in the multivariate analysis in comparison to the univariate model.

**Results section:**

**What are the p-values for the Kaplan Meier analysis?**

*Response: The methods section has been supplemented with the description of statistical test in KM analyses (log-rank test), and the corresponding p values have been added to the results section:*

*“Kaplan-Meier analysis*

*Kaplan-Meier analysis of both the unweighted and the weighted data ~~show a large improvement~~ indicated higher overall survival in the intervention cohort, with apparent survival benefits from the first 6 months of the study (Fig 2). The difference in the unweighted analysis was not statistically significant using the log-rank test (p=0.18), but it was statistically significant in the weighted analysis (p=0.049).*

*Consistency of statistical significance in the KM analysis and Cox regression is also briefly added to the Discussion in the current revision:*

*“The statistical hypothesis testing in the weighted KM and Cox regression were consistent (p=0.049 in the former and p=0.039 in the latter). One cannot expect exactly the same results as these tests are not identical, the first being a nonparametric test based on the comparison of the expected and observed numbers of the outcome, the second being a Wald test of a regression coefficient.”*

**For each analysis it needs to be clear what samples have been used.**

*Response: Thank you for this comment, we made the analyzed study populations fully explicit in the revised version, at the end of the methods section:*

*“All analyses were conducted on the weighted dataset, while the unweighted dataset was also analyzed in parallel with the weighted dataset at baseline balance description and in Kaplan-Meier analysis.“*

*To facilitate reading, the legend of Table 1 has also been supplemented with a corresponding clarification:*

*“Table 1. Baseline characteristics of the study population. Control and intervention cohorts refer to 12-month patient samples before and after OnkoNetwork implementation, respectively. For the Intervention cohort, unweighted and weighted data are identical.”*

**A statistical hypothesis test is not a descriptive analysis! (page 10, line 191) This is wrong and needs to be corrected.**

*Response: Thank you, we fully agree with your point, it was a misnomer. In the revised manuscript, Kaplan-Meier analysis is reported under a dedicated section, not mixed with descriptive analyses, and together with statistical hypothesis test results:*

*Kaplan-Meier analysis*

*Kaplan-Meier analys~~e~~is of both the unweighted and the weighted data ~~show a large improvement in 3-year~~indicated higher overall survival in the intervention cohort, with apparent survival benefits from the first 6 months of the study (Fig 2).* The difference in the unweighted analysis was not statistically significant using the log-rank test (p=0.18), but it was statistically significant in the weighted analysis (p=0.049).

*Consistency of statistical significance in the KM analysis and Cox regression is also briefly added to the Discussion in the current revision:*

*“The statistical hypothesis testing in the weighted KM and Cox regression were consistent (p=0.049 in the former and p=0.039 in the latter). One cannot expect exactly the same results as these tests are not identical, the first being a nonparametric test based on the comparison of the expected and observed numbers of the outcome, the second being a Wald test of a regression coefficient.”*

**Additional refinements of the manuscript based on co-author comments**

*Based on co-author feedback, we suggest a few additional clarifications in the manuscript, as summarized below:*

***Introduction, patient navigation in cancer: more clear wording is suggested***

*“Many European countries which recognize the importance of timely diagnosis and treatment in cancer patients have introduced maximum care delay thresholds for cancer patients~~waiting time targets in cancer care~~, including the UK [5] and Sweden [6]. Nevertheless, the evidence basis for introducing delay thresholds in cancer patient pathway intervals is frequently challenged in the scientific literature and the clinical consequence of care delays is not necessarily the same in ~~may be different according to~~ different cancer types [7-10]. To better understand the apparently contradicting evidence ~~Hence, it is important to understand potential sources of methodology bias~~ in lung cancer studies, potential sources of methodology bias are ~~as explained~~briefly overviewed in the next section. “*

***Introduction, Controversial findings of previous timeliness of care… studies: more clear description of potential RCT designs is suggested***

*“Randomized controlled studies on the clinical implications of reducing or increasing diagnostic and treatment delays in cancer care are scarce in the scientific literature, and intentional postponement of cancer treatment – that would be required in a randomized study – may be ethically disputable.”*

**Consistent use of robust standard errors in Cox regression models**

*During the revision of the manuscript, we noticed that robust standard errors were used in all except one Cox regression model. The only exception was the second multivariate Cox regression model adjusting for post-baseline patient characteristics (e.g., modalities of investigations conducted before treatment initiation, stage and functional status at treatment initiation, and treatment modalities applied, including surgical resection, chemotherapy, and radiotherapy as broad categories). In the current revised version, this inconsistency has been resolved and all Cox regression coefficient confidence intervals are calculated using robust standard errors. Consequently, confidence intervals of regression coefficients from the second multivariate Cox regression model have been slightly changed as indicated in Table 2, and these changes could also result in changes of significance for some of the investigated covariates, reflected by the below listed changes in the Results and Discussion sections of the manuscript. These corrections have no impact on the point estimates of regression coefficients, and* do not affect the lack of *significance of the tested intervention in the second multivariate Cox regression model adjusted to post-baseline patient characteristics.*

*Corresponding changes in the Results section:*

*“As expected, belonging to the intervention cohort was not significantly associated with survival benefit in this model (HR=0.81, ~~p=0.323~~ p=0.354), indicating that the included post-baseline patient path characteristics and intermediate outcomes at least partly explained the observed survival benefits of OnkoNetwork. Mortality was significantly lower ~~in patients undergoing resection surgery~~ in patients with documented AJCC TNM stage before treatment initiation (p=0.024) and in patients receiving chemotherapy (p=0.024), and significantly higher in patients with stage IV disease (p<0.001) and ECOG 1 (p=0.046) and 3-4 status (p=0.006) at treatment initiation (Table 2). Conducting a PET-CT investigation before treatment initiation, and having a resection surgery also tended to show some association with improved survival, without statistical significance (~~p=0.0550~~ p=0.098 and p = 0.079, respectively).”*

Table2: confidence intervals have been updated in the last column (second multivariate model)

*Corresponding changes in the Discussion section:*

*“To further investigate whether ~~this improvement~~ the observed association in the Cox regression model could be explained by intermediate outcomes in the putative causal chain, a subsequent multivariate weighted Cox regression model was adjusted to post-baseline patient characteristics. As expected, intervention effect was not statistically significant in the latter model, whereas mortality was significantly lower in patients with documented AJCC TNM stage before treatment initiation ~~in patients undergoing resection surgery~~ and in patients receiving chemotherapy, and significantly higher in patients with stage IV disease and/or ECOG 1 and 3-4 status at treatment initiation. The use of PET/CT before treatment initiation, and undergoing resection surgery also tended to be associated with longer overall survival (~~HR 0.62, 95%CI 0.38-1.01,~~* *HR 0.62, 95%CI 0.36-1.09 and HR 0.34, 95%CI 0.10-1.13, respectively*; *not significant). Accordingly, the beneficial effect of OnkoNetwork implementation on lung cancer overall survival could be – at least partly – explained by a complex interplay of multiple post-baseline factors including broader exploitation of PET-CT imaging, higher proportion of patients with resectable disease, and lower proportion of advanced stage ~~IV~~ patients at treatment initiation. Indeed, some corresponding ~~beneficial~~ non-significant trends after OnkoNetwork implementation could be observed in descriptive analyses (Figure 3~~, not significant~~).”*

*Figure3: This figure has been supplemented with descriptive charts of patient characteristics described above.*

*Finally, a few remaining typos were also identified and corrected in the current revision.*

We thank again for all the constructive and helpful comments of the Editor and the Reviewers to increase the scientific merit of our manuscript, and strongly hope that the revised manuscript will meet the high standards of PLOS ONE for publication.
