# Supplementary material for: Improved survival of non-small cell lung cancer patients after introducing patient navigation: A retrospective cohort study with propensity score weighted historic control
Source: PLoS One. 2022 Oct 25;17(10):e0276719. doi: 10.1371/journal.pone.0276719 (PMC9595513; doi:10.1371/journal.pone.0276719)
Supplement: Attachment — Submitted filename: Response to Reviewers.docx [file pone.0276719.s008.docx]

**Rebuttal Letter**

We thank the Editor and the Reviewers for their helpful comments on our manuscript. Below is our response to each additional point raised by Reviewer #2 and Reviewer #4. We hope that the corresponding revisions and the provided justifications are satisfactory and that the manuscript will be now suited for publication in PLOS ONE.

Our responses to Reviewers are typed in *Italic,* and *changes in manuscript text are typed in red with citation marks below.*

Sincerely, on behalf of all co-authors,

Prof. Zoltán Vokó

**Reviewer #2:**

**For counterfactual analysis, please mention if the baseline characteristics were selected from control cohort or intervention cohort. If selected from control cohort, is it possible that the survival benefit difference may increase or reduce between intervention and hypothetical cohort? If yes, then one of the possible explanation may be that owing to the propensity score matching using control group baselines characteristics may not make a difference for the analysis. Is there any other possible explanation? This should be discussed in the discussion.**

*Response: Thank you for your considerations on this interesting aspect of the presented counterfactual analyses. We agree that difference between the intervention and a hypothetical intervention cohort would be worth discussing, in case the baseline characteristics for the counterfactual analyses were selected from the control cohort. However, in the presented counterfactual analyses, the survival probabilities were predicted in the intervention cohort by setting their cohort indicator parameter from “intervention” to “control” before calculating the predictions (as already mentioned in lines 228-231 of the manuscript). Accordingly, the hypothetical cohort is a pseudo-control cohort in this study. This is why the difference between the intervention cohort and the counterfactual cohort was interpreted as an effect estimate of the investigated intervention in Table 3.*

*Nevertheless, following the logic of your suggestion, comparing the counterfactual, pseudo-control cohort to the true control cohort might also be interesting for the readers. Mean survival probability in the counterfactual cohort at 1, 2, and 3 years was consistent with the Kaplan-Meier plot of the control cohort. Accordingly, we added a corresponding statement to the discussion section, as follows (typed in red):*

*“The predicted survival in this counterfactual, hypothetical control cohort was consistent with the observed survival in the control cohort (data in Table 3 and Figure 2, respectively).”*

**Reviewer #4:**

**The authors present results from a study on the impact of patient navigation for non-small cell lung cancer patients in Hungary on survival. Authors use propensity score weighting to address imbalance in the group that had access to patient navigation and the historical control cohort that did not have access to patient navigation. Authors demonstrate improved survival in the group that had patient navigation, though this difference did not remain after accounting for post-baseline clinical measures (which showed some potential imbalance (though not statistically significant) even after weighting). The manuscript will be strengthened if authors consider the following points.**

*Response: Thank you for your review, and please find below our response to the specific points raised.*

**1. To avoid confusion between Table 1 sample sizes and the at risk numbers given for the K-M curves in Figure 2 for the weighted sample, authors may want to clarify that the weighted sample size is 120.**

*Response: Thank you for your suggestion, the legend of Figure 2 has been revised accordingly:*

*“Fig 2. Kaplan-Meier plots of overall survival of NSCLC patients. A, unweighted data; B, weighted data. In panel B, the sample size represents the rounded sum of weights.”*

*In addition, control cohort sample size and total weight are also clarified and made explicit in the supplementary tables. Accordingly, a revised “S4 Supplementary tables” file is attached to the revised manuscript version.*

**2. It is not clear to me why authors included all of the baseline variables in the Cox model, since the propensity score weighting was done to approximately balance these variables between groups.**

*Response: Indeed, the propensity score weighting was done to approximately balance the baseline variables between the study cohorts. Hence, it was assumed that a univariate Cox regression analysis of intervention effectiveness in the weighted dataset could be justified. A corresponding univariate analysis is reported in Table 2.*

*Our main rationale to present also a multivariate analysis adjusted for all baseline variables was that from this model, hazard ratios for other baseline characteristics could also be evaluated, supporting the reader to put our findings into context regarding other independent risk factors of mortality in the NSCLC population. Furthermore, not presenting the multivariate model could be interpreted as a methodology limitation of our study and could result in exclusion of our findings from future systematic reviews and meta-analyses. Hence, the Authors felt that including both the univariate and the multivariate Cox model on baseline predictors have additional value, although we acknowledge that these models may be considered redundant when solely the intervention effectiveness is considered.*

**3. Throughout the manuscript, authors state that groups are "similar" or distributions are "similar". A non-significant p-value does not prove a lack of difference between groups. This is a minor point, since readers can refer to the tables to evaluate how "similar" they feel groups are on certain characteristics, but authors should keep this in mind when reporting results.**

*Response: Thank you for your observation, we fully concur with your comment. The manuscript has been checked and all corresponding sentences are refined in the current revision, as follows:*

*“Study population characteristics*

*Of the 661 patients with suspected lung cancer, 296 NSCLC patients were included in the analyses (123 and 173 patients in the intervention and control cohorts, respectively; Fig 1). Enrollment into OnkoNetwork was 0% and almost 100% in the control and the intervention cohort, respectively. ~~The study cohorts showed similar distributions by~~ There was no statistically significant difference across study cohorts in age, sex, residence urbanization level and socioeconomic development, tumor histology, and pre-hospital delay (time from onset to first hospital cancer suspect code), with ~~similar~~comparable proportions of symptom-free, accidentally identified cases at clinical onset before propensity score weighting. However, the rate of completed prior chest CT and bronchoscopy investigations at study baseline showed a statistically significant difference between the cohorts (Table 1). These observed between-cohort differences were eliminated by the applied propensity score-based weighting (Table 1), resulting in Rubin’s B and R values within the recommended ranges of <25 and [0.5; 2], respectively [63].”*

*…*

*“In the two cohorts, ~~similar~~comparable proportions of patients received radiotherapy or active anticancer treatment in general. None of the above between-cohort differences reached statistical significance.”*

*…*

*“This finding means that if the same study was repeated 1000 times in ~~similar~~ the same population~~s~~, more than 970 of these hypothetical studies would report at least some survival benefit upon OnkoNetwork implementation.”*

**4. In my first read of the manuscript, I was a bit confused by Figure 3, since it wasn't clear to me what percentages were being shown. I thought initially it had to do with survival in the groups among those who had particular characteristics, since this was being shown after the Cox results. I realized that these are just the observed/weighted percentages in the two groups (as is presented in Supplementary Table S4a). Authors may want to refer readers to this table when mentioning Figure 3 in the text, to clarify.**

*Response: Thank you for your point, the axis label for Figure 3 has been supplemented to facilitate the proper interpretation of the figure:*

*“Fig 3. Between-cohort differences in post-baseline predictors of overall survival.* *The vertical axis indicates proportions of study subjects with the corresponding characteristics in the weighted dataset. For numeric data, please see Table S4a. “*

*Cross-checking the numeric data in supplementary tables with the revised statistical output file submitted in March 2022, a few typos were identified in the supplementary tables and corrected as well. Specifically: the control sample size was mistyped in Table S4b; proportions receiving any oncology treatment within 44 days after first cancer code (Table S4a), and largest tumor diameter at last assessment before treatment initiation (Table S4b) have also been corrected. These minor typo corrections did not affect the observed trends, neither the significance of between-group comparisons. The corrected supplementary tables file is part of the revised manuscript package.*

**Minor edits:**

**1. line 82: authors need to add a ")" after "groups" to close the parentheses from earlier in the sentence.**

**2. line 162: "Study data was" should be "Study data were"**

**3. line 190: "study data is" should be "study data are"**

**4. lines 238, 240: STATA should be Stata: https://www.statalist.org/forums/help#spelling**

**5. line 239: "using a" should just be "using"**

**6. Table 2: There are a couple of significant results that are not bolded, and a non-significant result that is bolded. Authors should be consistent in their use of bolded results. Also authors are missing a "]" for the CI for Urban residence in the multivariate baseline characteristics model.**

**7. line 397: "(Table 3) was" should be "(Table 3) were"**

*Response: Thank you for the careful review of our manuscript, the corresponding changes are implemented in the manuscript files.*

*Regarding bolded results in Table 2, we made the proposed corrections, with a single exception which requires additional justification: The 95% confidence interval of HR for age in the multivariate baseline model included 1, therefore, it was not considered significant and is not typed in bold. This is apparently in conflict with the significance of age in the univariate analysis, due to rounding of confidence interval limits to 2 digits in the table. Accordingly, the table legend has also been supplemented as follows:*

*“Table 2. Association between mortality and patient characteristics (weighted dataset).* *Bolded values indicate statistical significance (95%CI not including 1; note that numbers are rounded to 2 digits).”*

We thank again for all the constructive and helpful comments of the Editor and the Reviewers to increase the scientific merit of our manuscript, and strongly hope that the revised manuscript will meet the high standards of PLOS ONE for publication.
